# Mechanochemical Activation of a Bridged‐Exciplex Optical Force Probe

**DOI:** 10.1002/advs.77144

**Published:** 2026-08-19

**Authors:** Ronja Schumann, Andreas Bock, Felix Majer, Michael Baumann, Michael Olney‐Fraser, Lev Kazak, Fedor Jelezko, Alexander J. C. Kuehne, Robert Göstl

**Affiliations:** ^1^ Department of Chemistry and Biology University of Wuppertal Wuppertal Germany; ^2^ Institute of Macromolecular and Organic Chemistry Ulm University Ulm Germany; ^3^ Institute of Quantum Optics Ulm University Ulm Germany; ^4^ DWI – Leibniz Institute for Interactive Materials Aachen Germany

**Keywords:** exciplex, fluorescence, mechanochemistry, optical force probes, polymers

## Abstract

Strong demands are placed on optical force probes (OFPs) to detect bond scission on different timescales and in different microenvironments necessitating the development of detection strategies beyond simple on/off reporters. We here introduce OFPs based on bridged‐exciplex Diels‐Alder adducts. These exhibit fluorescence in both their latent (non‐activated) and activated states, distinguishing them from conventional “turn‐on” probes. The pronounced solvatochromic character of the latent form additionally enables its use as an environmental polarity sensor, with emission wavelength and intensity responding to the local environment. We incorporate these OFPs into linear chains and polymer networks. Mechanochemical activation is demonstrated both in solution and in the solid state, culminating in spatially resolved imaging of damage zones. Crucially, the activated and latent states differ not only in fluorescence intensity but also in quantum yield and fluorescence lifetime, enabling multimodal contrast. This multi‐feature OFP thus represents a promising technology for advanced imaging techniques including high‐resolution and lifetime‐resolved microscopy methods, substantially expanding the analytical toolkit available for mechanochemistry beyond the limitations of existing OFPs.

## Graphical abstract

A bridged‐exciplex optical force probe is synthesized, switching from solvatochromic charge‐transfer emission in the latent state to intense locally excited emission upon activation. This dual photophysical response with additional changes in quantum yield and fluorescence lifetime enables precise environmental sensing and distinct optical readouts. Incorporated into polymers, it enables multimodal, spatially resolved imaging of mechanochemical damage across diverse conditions.

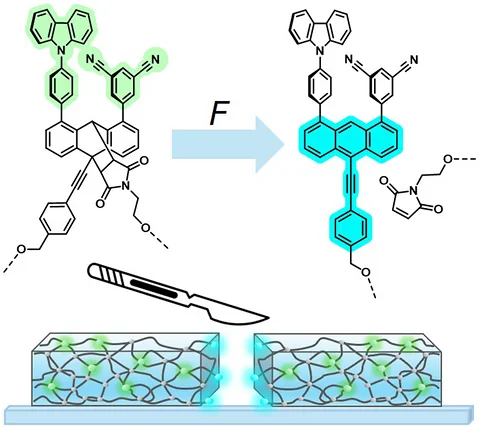

## 1 Introduction

The mechanical behavior of polymeric materials is governed across multiple length scales, from individual covalent bond stretching and scission to segmental rearrangements, network topology changes, and macroscopic failure [1, 2]. Understanding and detecting these processes with high fidelity is a central challenge in materials science, as polymer deformation and fracture are multiscale phenomena: macroscopic failure originates in the rupture of individual covalent bonds, yet the damage zone extends over hundreds of µm from the fracture plane [3, 4]. The quantitative relationship between molecular bond scission and macroscopic mechanical responses has only recently become experimentally accessible through the use of fluorogenic mechanophores, called optical force probes (OFPs) [5, 6, 7, 8, 9, 10]. Thus, damage detection at the molecular level has moved from an analytical convenience to a prerequisite for developing physically accurate models of mechanical behavior and rational material design [4, 11].

The ever‐increasing demands placed on OFPs, including detection at material‐relevant timescales, multi‐parameter readout, and operation under challenging conditions, necessitate the development of multifaceted detection strategies beyond simple on/off reporter behavior. Adapting ground‐state absorption or steady‐state emission spectra of chromophores is insufficient to meet these demands. Instead, advanced OFP design requires engineering of the excited‐state energy landscape to unlock access to more complex and informative photophysical phenomena.

Several strategies exploiting this paradigm of excited‐state engineering through mechanochemistry have emerged in recent years. Mechanochemical activation of triplet‐triplet annihilation photon upconversion (TTA UC) enables anti‐Stokes sensing with high signal‐to‐noise contrast, providing a spectral window entirely distinct from the excitation source and largely free from autofluorescence background [12, 13]. Excited‐state intramolecular proton transfer (ESIPT) OFPs offer highly sensitive ratiometric responses through force‐tuned tautomeric equilibria [14, 15, 16, 17, 18, 19, 20, 21]. Fluorescence lifetime imaging microscopy combined with Förster resonance energy transfer (FLIM‐FRET) using fluorescent‐protein‐based OFPs adds a lifetime dimension to force sensing [22]. Loop‐forming excimers exploit the distance‐dependence of excited‐state complex formation to deliver concentration‐independent, ratiometric monomer‐to‐excimer color changes upon mechanical unfolding, providing a continuous and reversible optical strain readout [23, 24, 25, 26, 27, 28]. Collectively, these methods illustrate that exploiting non‐trivial excited‐state pathways represents a decisive step toward next‐generation OFP performance.

Among these photophysical phenomena, bridged exciplexes represent a compelling and underexplored approach for OFP design. An exciplex, an excited‐state complex between an electron donor and acceptor in their electronically excited and ground states, respectively, exhibits emission characteristics that are inherently sensitive to the nanoscale environment, including polarity and geometry. Bridging such exciplexes with anthracene gives access to emission from a locally excited (LE) state in the activated OFP. In the latent state, the anthracene conjugation is broken and charge transfer (CT) emission occurs from the exciplex state [29, 30, 31]. This OFP concept with bimodal emission in the on and off state combines several advantages uniquely suited to mechanosensing: it produces fast emission with short photoluminescence lifetimes in the (sub)nanosecond regime, high color purity, and precise spectral discernability of the two states of which one (the CT state) is susceptible to environmental conditions [32, 33]. The force‐induced modulation of donor‐acceptor interactions therefore would offer a multiparameter optical readout that neither classical fluorophore activation nor simple Stokes‐shifted emission can deliver.

**SCHEME 1:**
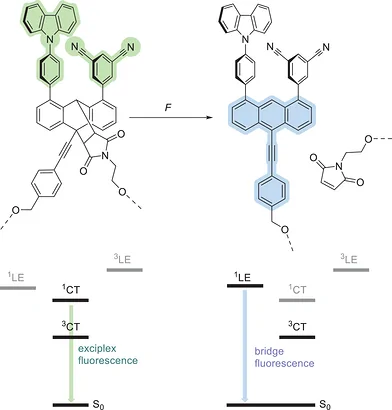
Force‐induced retro Diels‐Alder reaction of bridged‐exciplex OFP. In the Diels‐Alder adduct (off‐state), the OFP exhibits emission from the CT state of donor and acceptor. After mechanochemically induced bond‐scission, the bridge‐fluorescence of the LE state of the π‐extended anthracene is restored.

Here, we demonstrate the mechanochemical activation of a bridged‐exciplex emitter (Scheme 1). In the deactivated Diels‐Alder state, the emission of the exciplex originates from the charge transfer (CT) state of the aligned donor and acceptor moieties of the exciplex. Therefore, the emission is solvatochromic and shifts bathochromically with increasing polarity. By contrast, the LE state of the π‐extended anthracene bridge is not solvatochromic. This renders this OFP also suitable as an environmental probe, where the emission shifts hypsochromically during activation in a polar solvent and bathochromically in a nonpolar solvent.

## 2 Results and Discussion

### 2.1 Syntheses of Emitters and Polymers

**SCHEME 2:**
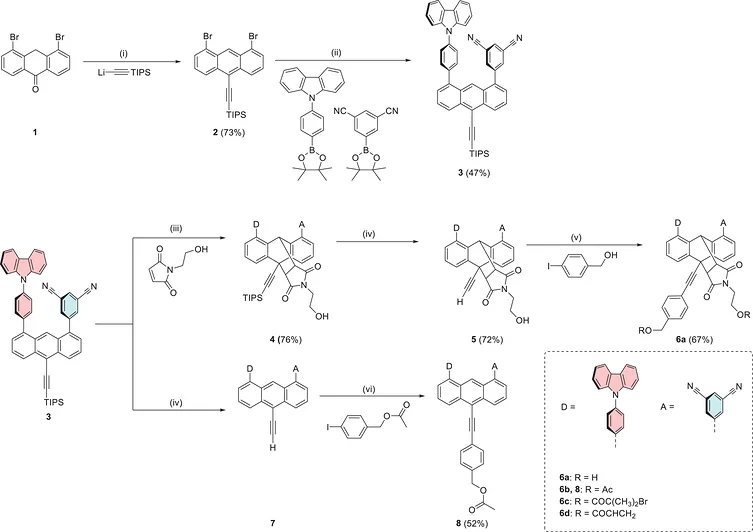
Synthesis scheme of the bridged‐exciplex mechanophore **6a** and the respective active small molecule model **8a**. (i) Toluene, 4 h, 60°C. (ii) K_3_PO_4_, Pd_2_(dba)_3_, P(*o*‐tol)_3_, 1,4‐dioxane:toluene:H_2_O (5.5:1.8:1.0), o.n., 80°C. (iii) Toluene, 12 d, 120°C. (iv) TBAF, THF, o.n., r.t. (v) NEt_3_, Pd(PPh_3_)_4_, DMF, 3 d, 100°C. (vi) NEt_3_, Pd(PPh_3_)_2_Cl_2_, CuI, THF, o.n., 60°C. The syntheses of **6b‐d** are specified in the Supporting Information.

For the bridged‐exciplex system, phenylcarbazole (PhCz) was selected as the electron donor and isophthalonitrile (IPN) as the acceptor. Density functional theory (DFT) calculations previously determined the frontier orbital energies as HOMO(PhCz) = −6.00 eV and LUMO(IPN) = −2.59 eV [31]. This combination of a relatively weak donor and a strong acceptor promotes intense blue emission originating from the LE state of the anthracene bridge in the activated OFP. At the same time, the deliberate choice of a weak donor ensures a comparatively low‐lying CT state, enabling emission in the inactive form of the OFP [31]. Different from previous published bridged‐exciplexes that were used as emitters beyond the spin‐statistical limit, we chose a π‐extended anthracene as the bridge moiety, to increase the photoluminescence quantum yield *Φ*_PL_ and shift the absorption and emission of the OFP bathochromically from the UV to the visible region [34]. The syntheses of OFP **6** and its corresponding activated small molecule model compound **8** are depicted in Scheme 2 and molecular characterization data are shown in Figures S9–S38. Notably, the synthesis features a one‐pot approach, in which anthracene is coupled to the electron donor and acceptor moieties in one step. Consequently, a yield of 47% (close to the theoretical limit of 50%) can be attained, due to the statistical nature of the coupling, as opposed to the theoretical limit of 33% when choosing a sequential coupling pathway.

**FIGURE 1:**
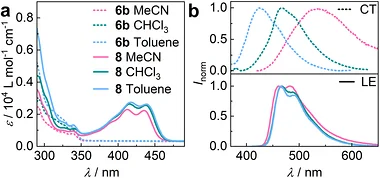
(a) UV–Vis absorption and (b) emission spectra of latent mechanophore **6b** and activated small molecule **8** model compounds in solvents with different polarity (MeCN, CHCl_3_, toluene). While CT emission of **6** is strongly solvatochromic, LE emission of **8** is barely affected by solvent choice.

It has been previously demonstrated that the LE emission of the anthracene bridge is insensitive to solvent polarity, whereas the CT emission of the bridged exciplex exhibits pronounced solvatochromism, undergoing a bathochromic shift with increasing solvent polarity. Upon formation of the Diels‐Alder adduct, the LE state becomes inaccessible, and emission instead arises from the CT state. This transition enables the observation of solvatochromic behavior, which we demonstrate by fluorescence spectroscopy in solvents of varying polarity, namely MeCN, CHCl_3_, and toluene (Figure 1). To mimic the incorporation of the mechanophore into the polymer, the small molecules were acetylated, providing compound **6b** and **8**.

The efficient masking of anthracene absorption and associated LE emission in the latent Diels‐Alder adduct **6b** is confirmed by UV‐Vis and fluorescence spectroscopy: The characteristic anthracene absorption band (350‐475 nm) is absent in **6b**, yet prominent in the active form **8**. Fluorescence spectra of **6b** exhibit the expected solvatochromism of CT emission, with the emission maximum *λ*_PL_ shifting by ≈110 nm from nonpolar toluene to polar MeCN (Figure 1 and Table 1). By contrast, LE emission of **8** remains insensitive to solvent polarity. Consequently, OFP activation induces a hypsochromic shift in polar solvents or a bathochromic shift in nonpolar solvents. Notably, the CT state in the latent form exhibits low *Φ*_PL_ between ca. 6 and 12% with fluorescence lifetimes (*τ*) between 82 and 31 ns. The LE state of the activated form has higher *Φ*_PL_ between 74 and 84% and *τ* that decrease significantly (Table 1 and Figure S4). Hence, especially at higher polarity of the environment, *τ* differs significantly between the latent and the activated OFP, highlighting the analytical potential for this modality.

**TABLE 1:** Photophysical properties, photoluminescence maximum *λ*_PL_ (excited at *λ*_exc_) for **6b** and **8** together with the fluorescence lifetimes *τ* and quantum yields *Φ*_PL_ of the small molecule model compounds **6c** and **8** in different solvents. *τ* and *Φ*_PL_ were measured in degassed solution.

molecule	solvent	*λ*_exc_ / nm	*λ*_PL_ / nm	*τ* / ns	*Φ*_PL_ / %
**6**	MeCN	330	538 (**6b**)	82 (**6c**)	5.8 (**6c**)
**6**	CHCl_3_	330	466 (**6b**)	44 (**6c**)	13.7 (**6c**)
**6**	Toluene	330	424 (**6b**)	31 (**6c**)	11.7 (**6c**)
**8**	MeCN	400	461, 483	5.6	80.6
**8**	CHCl_3_	400	467, 488	3.9	74.3
**8**	Toluene	400	466, 488	4.0	83.6

**FIGURE 2:**
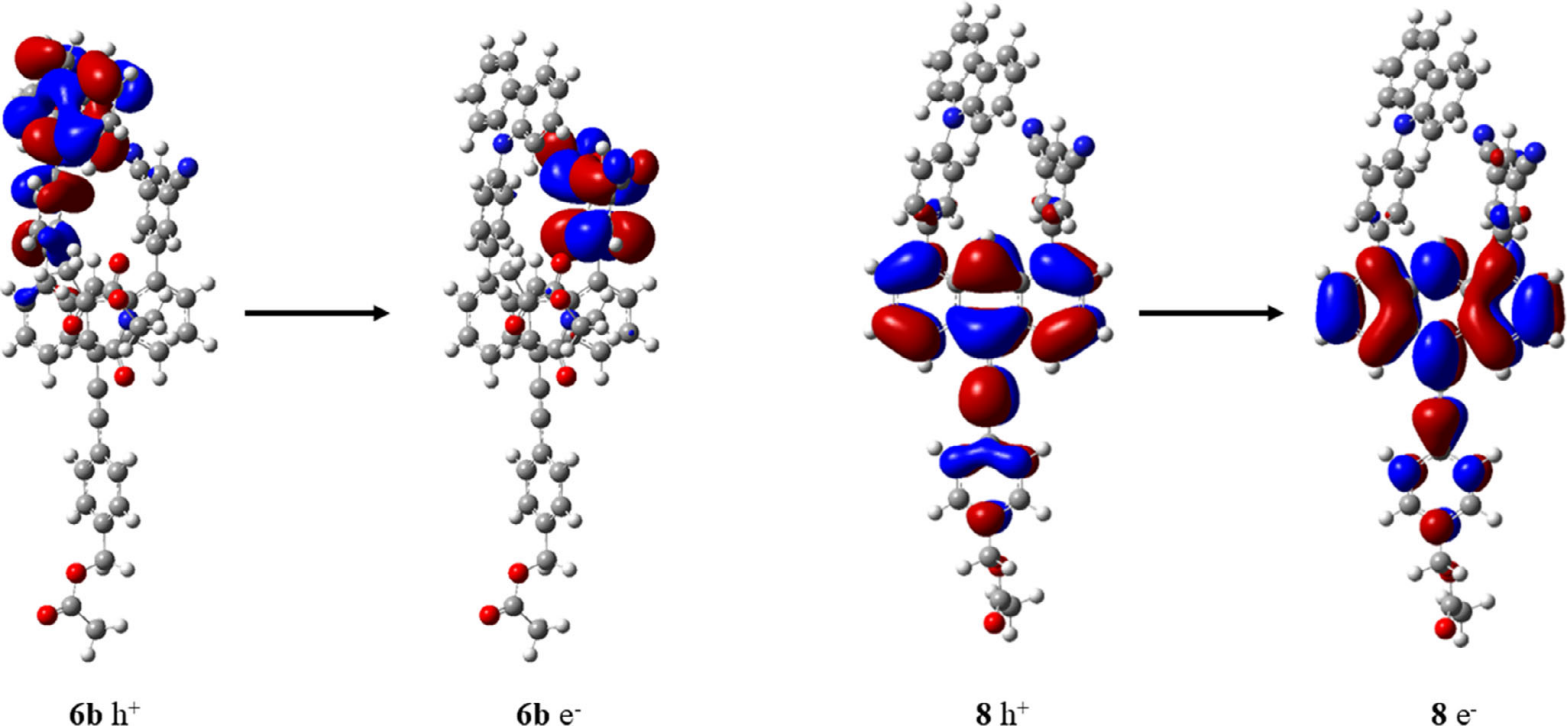
NTOs with hole (h^+^) and electron (e^−^) of compounds **6b** and **8** in the ground state geometry, calculated by TD‐DFT at the LC‐ωPBE/6‐31G(d) level of theory.

We computed the natural transition orbitals (NTOs) of **6b** and **8** using time‐dependent density functional theory (TD‐DFT) at the LC‐ωPBE/6‐31G(d) level of theory to support the experimental results (Figure 2). For the Diels‐Alder adduct **6b**, the NTOs show a clear CT state from the PhCz donor to the IPN acceptor. For the active compound **8**, we observe an LE state, where the electron density is mainly located on the anthracene bridge. The calculated NTOs correspond well with the experimentally determined absorption bands and the observed solvatochromism (Table S1).

For incorporation of the OFP into linear poly methacrylate (PMA), diol **6a** was first functionalized with α‐bromoisobutyryl bromide to introduce polymerizable end groups. The resulting bifunctional initiator **6c** was used in a Cu‐mediated supplemental activator and reducing agent atom transfer radical polymerization (SARA ATRP) [6], ensuring precise placement of the OFP at the center of the polymer chain. To incorporate the OFP into polymer networks, **6a** was additionally acryloylated to afford the corresponding diacrylate crosslinker **6d**. This compound was then used as a co‐crosslinker together with non‐functional tetraethylene glycol diacrylate (TEGDA) for oligo(ethylene glycol) methyl ether acrylate (OEGMEA, *M_n_* ≈ 480 Da) as well as hexyl acrylate (HA) to generate the corresponding POEGMEA and PHA networks.

Because mechanophore **6** showed signs of partial degradation under the conditions required for conventional thermal radical initiation at 60°C, we performed thermal stability tests by dynamic scanning calorimetry (DSC) and variable‐temperature (VT) NMR spectroscopy. We found that **6** showed no signs of unusual degradation, e.g., from retro Diels‐Alder reaction, by heating alone (Figures S1 and S2). Also, aforementioned SARA ATRP underscored reasonable inertness of **6** against radical presence. However, in a heating experiment in the presence of AIBN we observed an unidentifiable fluorescent byproduct (Figure S3) underscoring that heating in the presence of radicals led to partial unwanted degradation of **6**. Therefore, bulk polymerization was carried out at room temperature using the photoinitiator 2‐hydroxy‐4‘‐(2‐hydroxyethoxy)‐2‐methylpropiophenone (I2959).

### 2.2 Mechanochemical Activation

**FIGURE 3:**
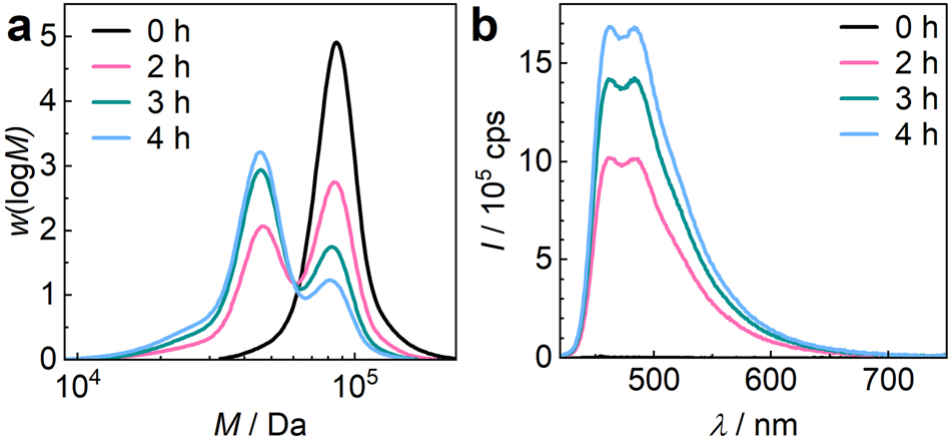
PMA‐functionalized OFP **6** over the course of its sonication. (a) GPC MMDs (refractive index detector). (b) Emission spectra of LE state (*λ*_exc_ = 400 nm).

To investigate the mechanochemical behavior of the bridged‐exciplex, the PMA‐functionalized OFP was dissolved in MeCN and sonicated using an immersion probe sonicator (20 kHz, 30% of the max. amplitude, 4 h, 1 s on, 1 s off pulses). After 2, 3, and 4 h, samples were withdrawn and analyzed by UV‐Vis and fluorescence spectroscopy as well as gel permeation chromatography (GPC). The results are depicted in Figure 3. Both, GPC and fluorescence spectroscopy confirm the mechanochemical activation of the OFP. Already after 2 h the bridge emission of the activated OFP is clearly visible. GPC molar mass distributions (MMDs) confirm the reasonably selective scission at the chain center, with a new peak at ≈40 kDa corresponding to ca. half of the initial 83 kDa. The mechanochemical selectivity was determined to 71% (Figures S7 and S8). A negative control sonication of the small molecule **6b** under the same conditions led to no conceivable activation (Figure S5).

**FIGURE 4:**
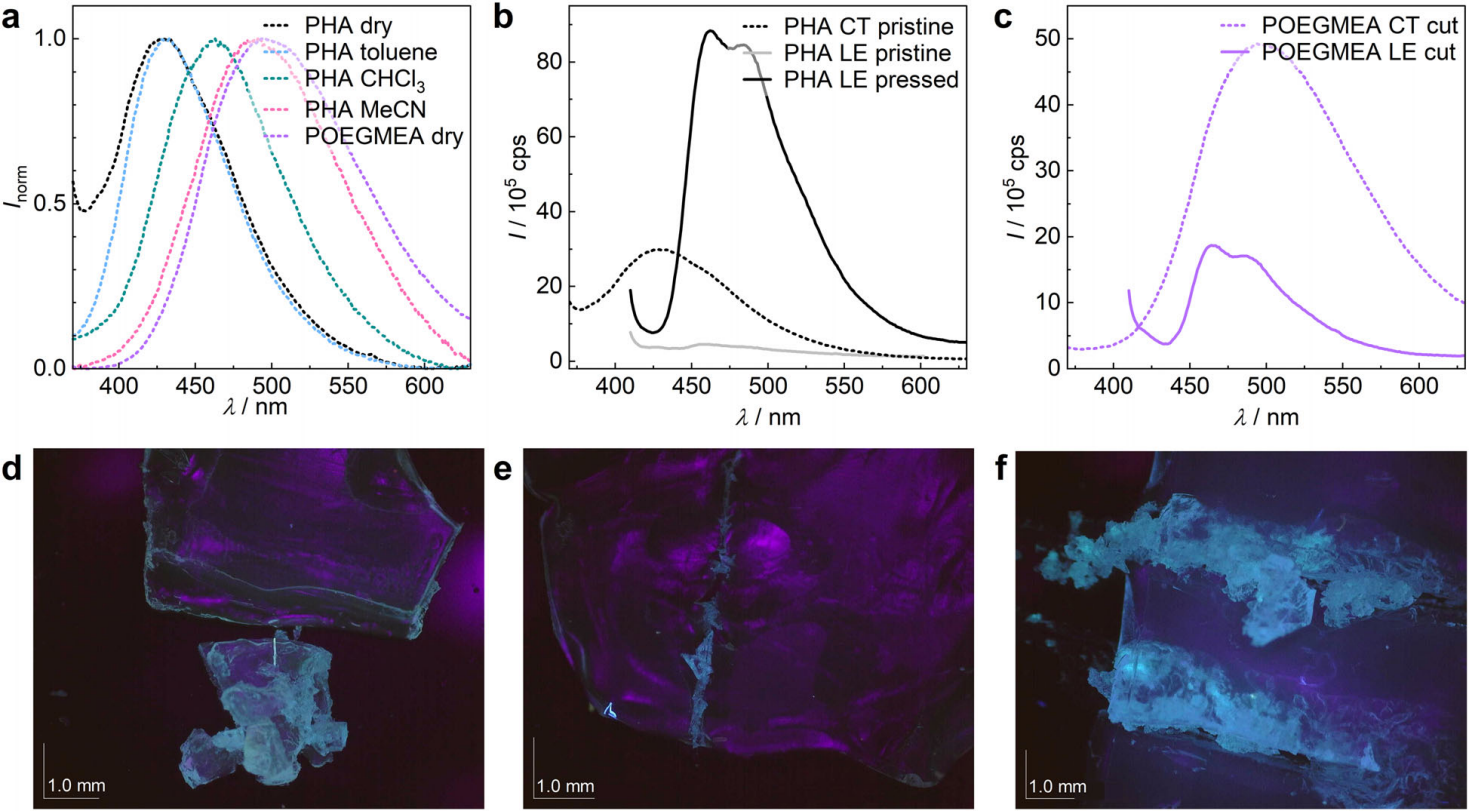
(a) CT emission spectra of pristine PHA networks with 0.1 mol% OFP co‐crosslinker **6d**, dry and swollen in different solvents as well as dry POEGMEA network with 0.1 mol% **6d**. (b) Emission spectra of pristine and pressed PHA (*λ*_exc, CT_ = 340 nm, *λ*_exc, LE_ = 400 nm). (c) Emission spectra of POEGMEA network activated by cutting (*λ*_exc, CT_ = 340 nm, *λ*_exc, LE_ = 400 nm). (d) Fluorescence micrograph (*λ*_exc_ = 400 nm) of pristine (top) and bulk activated (bottom) PHA. (e) Fluorescence micrograph (*λ*_exc_ = 400 nm) of PHA activated by cutting. (f) Fluorescence micrograph (*λ*_exc_ = 400 nm) of POEGMEA activated by cutting.

To investigate the OFP properties in the solid state, PHA and POEGMEA networks were analyzed. The PHA network provided a nonpolar microenvironment, while the POEGMEA network offered a polar one. Solid‐state fluorescence spectroscopy of the dry networks revealed emission profiles consistent with the small molecule reference: the PHA network exhibited an emission range comparable to that observed in toluene, whereas the POEGMEA network matched the emission profile recorded in MeCN (Figure 4a). Swelling of the PHA network in toluene, CHCl_3_, or MeCN induced a bathochromic shift of the CT emission analogous to that observed for the small molecular bridged‐exciplexes in solution.

Bulk mechanical activation of the dry networks was performed using a hydraulic press (10 t, 30 s) or cutting, after which both networks exhibited LE emission (Figure 4b,c), confirming that solid‐state OFP activation is achievable. These results further highlight a key advantage of this OFP design: the emission can be switched both hypsochromically upon activation in a polar environment and bathochromically upon activation in a nonpolar environment, providing a versatile, environment‐dependent optical response.

To demonstrate spatially resolved activation, a portable DinoLite fluorescence microscope was employed. Compression (Figure 4d) and cutting (Figure 4e,f and Video S1) of the networks revealed localized LE emission exclusively at the damaged site, confirming targeted OFP activation. Control experiments employing networks crosslinked exclusively with non‐functional TEGDA confirmed that the observed emission originates solely from OFP activation (Figure S6). Cutting the network samples revealed another feature of this OFP: if the sample is just partially activated, both CT and LE can still be separately excited and observed using the same detector settings (Figure 4c). The distinctly higher *Φ*_PL_ of the LE emission compensates for the expectedly low activated fraction of OFPs inherent to polymer networks, where typically only ≈0.1 to 1% of all bonds are broken (Table 1) [35]. As a result, the CT and LE emission intensities remain comparable despite the large disparity in their relative population, constituting a clear advantage over previous multicolor OFPs [6].

## 3 Conclusion

In summary, we have introduced a new excited‐state‐tailored OFP with a unique combination of features enabling observation of bond scission across multiple analytical dimensions. The OFP was successfully incorporated into both linear PMA and crosslinked networks of varying polarity, including PHA and POEGMEA. Mechanochemical activation was demonstrated in solution by ultrasonication, as well as in the solid state through bulk compression and cutting.

In its latent state, the OFP exhibits solvatochromic CT emission with a low *Φ*_PL_, rendering it simultaneously useful as an environmental polarity sensor. Upon activation, the retro Diels‐Alder reaction liberates the anthracene bridge, which displays intense LE fluorescence with a markedly higher *Φ*_PL_. The large difference in *Φ*_PL_ between the two states means that, upon targeted activation, for example by cutting, both CT and LE emissions can be observed within a comparable intensity range in the same sample.

Collectively, this OFP provides a multi‐parameter readout encompassing distinctive excitation and emission wavelengths, fluorescence intensities, and *Φ*_PL_ for both the off and on states. Furthermore, the difference in fluorescence lifetimes between the latent and activated forms presents a particularly promising avenue for future investigation, positioning this probe as a compelling candidate for FLIM and other time‐resolved imaging modalities.

## Author Contributions

**Felix Majer**: Writing – review and editing, investigation, methodology, validation, formal analysis. **Andreas Bock**: investigation, methodology, formal analysis, visualization, writing – review and editing, validation, writing – original draft. **Fedor Jelezko**: supervision, resources, formal analysis, writing – review and editing, methodology. **Robert Göstl**: writing – review and editing, writing – original draft, funding acquisition, supervision, resources, data curation, project administration, visualization, methodology, conceptualization. **Ronja Schumann**: writing – original draft, writing – review and editing, data curation, visualization, validation, methodology, investigation, formal analysis. **Michael Baumann**: methodology, investigation, visualization, writing – review and editing, software. **Michael Olney‐fraser**: investigation, writing – review and editing, methodology, validation. **Alexander J. C. Kuehne**: funding acquisition, writing – review and editing, writing – original draft, validation, resources, supervision, project administration, methodology, conceptualization. **Lev Kazak**: writing – review and editing, methodology, investigation, validation, formal analysis.

## Funding

The authors acknowledge support by the state of Baden‐Württemberg through bwHPC and the Deutsche Forschungsgemeinschaft (DFG, German Research Foundation) – Project numbers INST 40/575‐1 FUGG (JUSTUS 2 cluster), 445471845, 445471097, 445470598 (A.J.C.K.), 527252926 (R.S., A.B., R.G., and A.J.C.K.,), and 503981124 (R.G.).

## Conflicts of Interest

The authors declare no conflicts of interest.

## Supporting information




**Supporting File 1**: advs77144‐sup‐0001‐SuppMat.pdf.

**Supporting File 2**: advs77144‐sup‐0002‐VideoS1.mp4.

## Data Availability

All data of this study are documented within the manuscript and its Supporting Information and are publicly available in Zenodo at https://doi.org/10.5281/zenodo.20340753.
