# Supplementary material for: Mechanochemical Activation of a Bridged‐Exciplex Optical Force Probe
Source: Adv Sci (Weinh). 2026 Aug 19:e77144. Online ahead of print. doi: 10.1002/advs.77144 (PMC13488482; doi:10.1002/advs.77144)
Supplement: Supplementary file 1 [file ADVS-9999-e77144-s001.pdf]

Supporting Information

for

Mechanochemical Activation of a Bridged-Exciplex Optical Force Probe

Ronja Schumann, Andreas Bock, Felix Majer, Michael Baumann, Michael Olney-Fraser, Lev Kazak, Fedor Jelezko, Alexander J. C. Kuehne, and Robert Göstl**

R. Schumann, R. Göstl

Department of Chemistry and Biology, University of Wuppertal, Wuppertal, Germany

E-mail: goestl@uni-wuppertal.de

A. Bock, F. Majer, M. Baumann, A. J. C. Kuehne

Institute of Macromolecular and Organic Chemistry, Ulm University, Ulm, Germany

E-mail: alexander.kuehne@uni-ulm.de

M. Olney-Fraser, L. Kazak, F. Jelezko

Institute of Quantum Optics, Ulm University, Ulm, Germany

R. Göstl

DWI – Leibniz Institute for Interactive Materials, Aachen, Germany

1 Table of Contents

1 Table of Contents 2
2 Materials and Methods 4
2.1 Materials 4
2.2 Methods 4
3 Syntheses 6
3.1 1,8-Dibromo-9,10-anthraquinone 6
3.2 1,8-Dibromo-10-anthrone **1** 6
3.3 ((4,5-Dibromoanthracen-9-yl)ethynyl)triisopropylsilane **2** 7
3.4 5-(8-(4-(9H-Carbazol-9-yl)phenyl)-10-((triisopropylsilyl)ethynyl)anthracen-1-yl)isophthalonitrile **3** 7
3.5 5-(8-(4-(9H-Carbazol-9-yl)phenyl)-10-((triisopropyl-silyl)ethynyl)anthracen-1-yl)isophthalonitrile *N*-(2-hydroxyethyl)-maleimide 9,10-Diels-Alder adduct **4** 8
3.6 5-(8-(4-(9H-Carbazol-9-yl)phenyl)-10-ethynylanthracen-1-yl)isophthalonitrile *N*-(2-hydroxyethyl)maleimide 9,10-Diels-Alder adduct **5** 9
3.7 5-(8-(4-(9H-Carbazol-9-yl)phenyl)-10-((4-(hydroxymethyl)phenyl)ethynyl)anthracen-1-yl)isophthalonitrile *N*-(2-hydroxyethyl)maleimide 9,10-Diels-Alder adduct **6a** 10
3.8 5-(8-(4-(9H-Carbazol-9-yl)phenyl)-10-((4-(acetoxymethyl)phenyl)ethynyl)anthracen-1-yl)isophthalonitrile *N*-(2-acetoxyethyl)maleimide 9,10-Diels-Alder adduct **6b** 11
3.9 4-((4-(4-(9H-Carbazol-9-yl)phenyl)-5-(3,5-dicyanophenyl)-anthracen-9-yl)ethynyl)benzyl 2-bromo-2-methylpropa-noate *N*-[2-(*α*-isobutyryloxy)ethyl)maleimide 9,10-Diels-Alder adduct **6c** 12
3.10 5-(8-(4-(9H-Carbazol-9-yl)phenyl)-10-((4-(acryloyloxymethyl)phenyl)ethynyl)anthracen-1-yl)isophthalonitrile *N*-(2-acryloyloxyethyl)maleimide 9,10-Diels-Alder adduct **6d** 13
3.11 5-(8-(4-(9H-Carbazol-9-yl)phenyl)-10-ethynylanthracen-1-yl)isophthalonitrile **7** 14
3.12 4-((4-(4-(9H-Carbazol-9-yl)phenyl)-5-(3,5-dicyanophenyl)-anthracen-9-yl)ethynyl)benzyl acetate **8** 14
4 Polymerizations 16
4.1 Linear Polymers 16
4.2 Polymer Networks 16
4.2.1 PHA Networks 16
4.2.2 POEGMEA Networks 16
5 Supporting Analytical Data 17
5.1 Dynamic Scanning Calorimetry 17
5.2 Variable-Temperature Nuclear Magnetic Resonance Spectroscopy 17
5.3 Heating Experiment with AIBN 18
5.4 Calculated Ground State Energies 18
5.5 Lifetime Measurements 19

5.6 Control Sonication of **6a** 19
5.7 Control Polymer Networks 20
5.8 Mechanochemical Selectivity 20
5.9 Nuclear Magnetic Resonance Spectroscopy and Mass Spectrometry 23
6 References 43

2 Materials and Methods

2.1 Materials

All commercially available chemicals were purchased from Sigma Aldrich, TCI Deutschland GmbH, VWR International, Fischer scientific, Carl Roth GmbH & Co., Acros Organics and Alfa Aesar and were used without further purification. Anhydrous solvents were dried prior to use in a MBraun SPS-800 instrument.

1,8-Diamino-9,10-anthraquinone was synthesized according to a procedure from Greussing *et al.*[1] 4-Iodobenzyl acetate was synthesized according to Wang *et al.*[2] 5-(4,4,5,5-Tetramethyl-1,3,2-dioxaborolan-2-yl)iso-phthalonitrile was synthesized according to Lee *et al.*[3]

2.2 Methods

Column chromatography was performed with a Reveleris Grace X2 automated column as well as a Puriflash Interchim 450 with silica columns F0025 and F0040, particle size 30 µm.

Photopolymerization was performed using a UV lamp (365 nm, 36 W, 50 Hz).

NMRs were recorded at room temperature with a 400 MHz Bruker Avance 400 NMR spectrometer and a Bruker Ascend 400 spectrometer (^{1}H: 400 MHz, ^{13}C: 101 MHz) at 293 K or at variable temperature, as indicated.

Field desorption (FD) mass spectrometry was performed on a JEOL AccuTOF-GCX high-resolution time-of-flight mass spectrometer.

HR-MS (MALDI FTICR) spectra were measured with a Bruker SolariX containing a DCTB matrix.

Analytical gel permeation chromatography (GPC) was performed using an Agilent 1260 Infinity system equipped with a diode array detector (DAD, G7115A) and a refractive index detector (RID, G7162A). Tetrahydrofuran (THF, Fisher Scientific) was used as eluent at a flow rate of 1 mL min^{-1} through a precolumn (8×50 mm, 5 µm particle size) and two analytical columns (8×300 mm, 5 µm particle size) in series. Samples were prepared at a concentration of 1 g L^{-1} and injected at a volume of 100 µL. Molar mass distributions were determined relative to poly(methyl methacrylate) (PMMA) standards in the range of 800 Da to 2200 kDa. Data acquisition and evaluation were performed using PSS WinGPC UniChrom software.

Differential scanning calorimetry (DSC) was conducted using a DSC1 STARe System (Mettler Toledo). Samples of approximately 10 mg were sealed in 40 µL aluminum crucibles. Measurements were performed under air or Ar atmosphere over a temperature range of 25-150 °C, comprising one heating and one cooling cycle at a rate of 1 K min^{-1}.

Sonication experiments were performed in a VibraCell ultrasonic processor VCX500 with a 13 mm solid full-wave probe, 254 mm long, frequency f = 20 kHz, and 30% of the maximum amplitude of 125 µm purchased from Sonics & Materials in a Suslick vessel purchased from Sonics & Materials under inert atmosphere while cooling with an ice bath.

UV-Vis absorption spectroscopy was performed using a Jasco V-670 double-beam spectrophotometer. Measurements were conducted in quartz cuvettes with a 1 cm path length at a sample concentration of 10 µM.

Photoluminescence quantum yields were determined on a HAMAMATSU Quantaurus-QY with a concentration of ~10^{-5} M.

The molecular lifetimes were measured using a tunable Ti:Sa fs pulsed laser emitting at 680 nm with a repetition rate of 80 MHz. The pulses were sent into a second harmonic generator which produced photons in the absorption band of the molecules at 430 nm. The pulses were guided into the sample and the fluorescence from the molecules in solution was focused using a condenser lens into an Excelitas avalanche-photo-diode (APD) which counted single photons. The APD was filtered using an OD5 neutral density filter as well as with a 450 nm long pass filter. The signals from the APD were sent to the stop channel of a FastComTech MSC8A fast counting card and their arrival times relative to the synch output from the Ti:Sa laser pulses were binned into histograms with a 100 ps bin-width. The fundamental repetition rate of the laser could not be altered and as a result, molecules with lifetimes longer than 6 ns could not be accurately measured using the setup as the full exponential decay curve could not be observed within the 12.5 ns inter-pulse spacing. However, shorter lifetimes could be accurately measured at this repetition rate.

Time-resolved emission was recorded under periodic pulsed excitation with a pulse separation of 12.5 ns. To analyze the pulse train of decays, we employed a model that explicitly accounts for residual emission from all preceding excitation pulses. The fourth decay curve in the measurement window, with intensity $I(t)$, was fitted using

$$I(t) = y_0 + \sum_{n=0}^{5} A \exp\left(-\frac{t + n \cdot 12.5\,ns}{\tau}\right),$$

where y_0 is a constant offset identical to the determined background median, A is a common amplitude, and τ is the fundamental lifetime of the molecule. By constructing the fit as a sum of identical exponential decays shifted by integer multiples of the laser period, overlapping fluorescence contributions from earlier pulses that extend into the time window of the fourth decay are taken into account. However, due to the repetition rate, longer lifetimes cannot be fit with a low uncertainty.

Fluorescence spectroscopy was conducted using a Horiba FluoroMax-4 spectrofluorometer. Fluorescence measurements were performed at a sample concentration of 1 µM in quartz cuvettes with a 1 cm path length. Spectral data were evaluated using the FluorEssence software. All solvents used for spectroscopic measurements were of spectroscopy grade and purchased from Fisher Scientific.

Fluorescence microscopy was performed using a DinoLite Edge digital microscope (AM4115T-CFVW) with an excitation wavelength of 400 nm and emission collected through a 430 nm long-pass filter. Images were acquired and processed using DinoCapture 3.0 software.

The theoretical calculations were performed with Gaussian 16, Revision C.01.[4] Ground-state geometries were optimized at the DFT level using the B3LYP functional and then read from the corresponding checkpoint files for the TD-DFT calculations (Geom=AllCheck). Vertical excitation energies and oscillator strengths were obtained using TD-DFT with the long-range corrected LC-ωPBE functional and the 6-31G(d) basis set (LC-ωPBE/6-31G(d)). Solvent effects were treated with the polarizable continuum model (PCM) using MeCN, $CHCl_3$, and toluene in a non-equilibrium solvation formalism for the excited states. The ground-state SCF calculations employed a restricted closed-shell formalism (RLC-ωPBE) with an RMS density convergence threshold of 1×10^{-8} and a maximum of 3000 SCF cycles, and vertical excitations were computed with TD(50-50, NStates = 6), yielding the six lowest singlet and six lowest triplet states.

3 Syntheses

3.1 1,8-Dibromo-9,10-anthraquinone

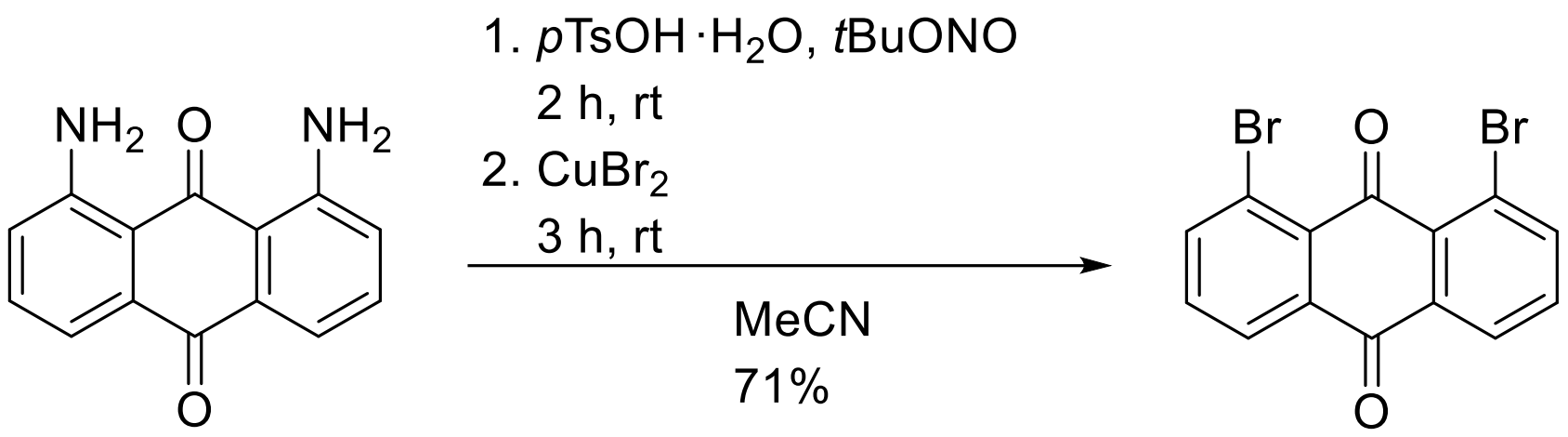

Under inert atmosphere, 1,8-diamino-9,10-anthraquinone (37.6 mmol, 8.97 g, 1.00 equiv.) and *p*-toluenesulfonic acid monohydrate (113 mmol, 21.5 g, 3.00 equiv.) were dissolved in dry MeCN (200 mL). To this, *tert*-butyl nitrite (84.7 mmol, 8.73 g, 10.1 mL, 2.25 equiv.) was added and the solution was stirred for 2 h. Afterwards, $CuBr_2$ (84.7 mmol, 18.9 g, 2.25 equiv.) was added and the reaction was continued for an additional 3 h. The mixture was then poured into HCl (2.4 M, 1000 mL) and the precipitate was filtered. Subsequent column chromatography (SiO_2, petroleum ether:CH_2Cl_2 = 1:4) afforded pure 1,8-Dibromo-9,10-anthraquinone (26.8 mmol, 9.82 g, 71%) as an orange solid. **^{1}H NMR (400 MHz, $CDCl_3$):** δ/ppm = 8.25 (dd, $^3J_{H\text{-}H}$ = 7.75 Hz, $^4J_{H\text{-}H}$ = 1.25 Hz, 2H, Ph-*H*), 8.03 (dd, $^3J_{H\text{-}H}$ = 8.00 Hz, $^4J_{H\text{-}H}$ = 1.25 Hz, 2H, Ph-*H*), 7.55 (dd, $^3J_{H\text{-}H}$ = 8.00 Hz, $^3J_{H\text{-}H}$ = 7.75 Hz, 2H, Ph-*H*). See Figure S9.

Further analytics are in accordance with the literature.[5]

3.2 1,8-Dibromo-10-anthrone **1**

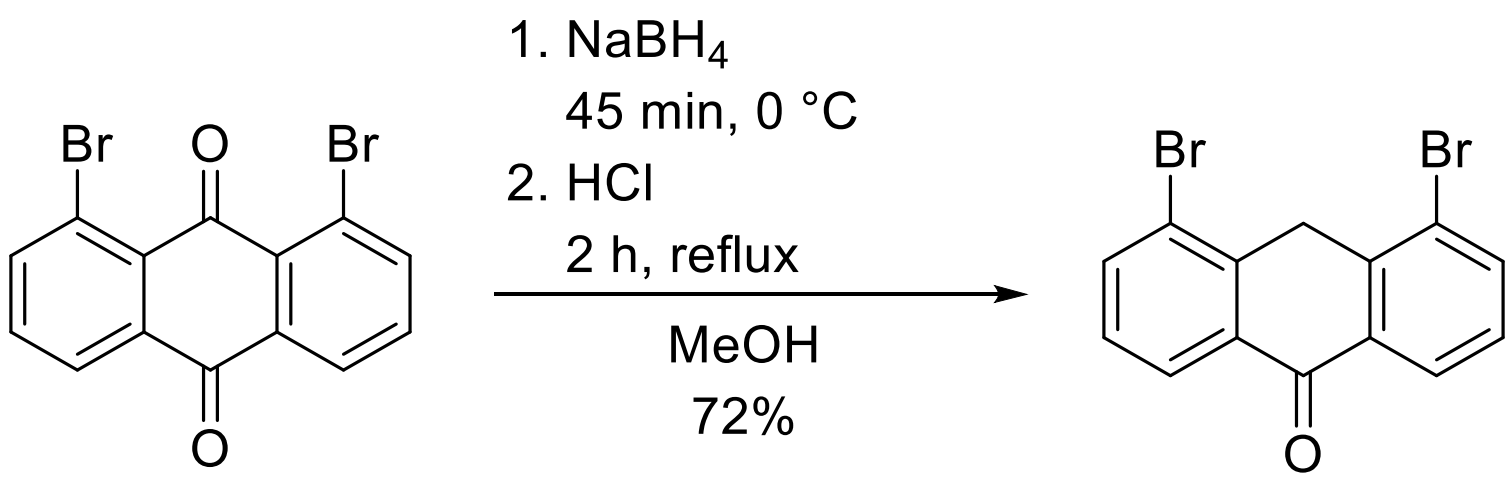

1

1,8-Dibromo-9-anthraquinone (26.6 mmol, 9.73 g, 1.00 equiv.) was dissolved in MeOH (750 mL) and cooled with an ice bath. $NaBH_4$ (133 mmol, 5.03 g, 5.00 equiv.) was added in small portions over the course of 25 min and the reaction was then stirred for additional 30 min. Afterwards, HCl (12 M, 88 mL) was added, and the solution was refluxed for 2 h. The solution was then concentrated *in vacuo* and poured into H_2O (1000 mL). Subsequent filtration of the precipitate and column chromatography (SiO_2, petroleum ether:CH_2Cl_2 = 6:4) provided **1** (19.0 mmol, 6.70 g, 72%) as a pale-yellow solid. **^{1}H NMR (400 MHz, $CDCl_3$):** δ/ppm = 8.36 (dd, $^3J_{H\text{-}H}$ = 7.88 Hz, $^4J_{H\text{-}H}$ = 1.31 Hz, 2H, Ph-*H*), 7.92 (dd, $^3J_{H\text{-}H}$ = 7.85 Hz, $^4J_{H\text{-}H}$ = 1.31 Hz, 2H, Ph-*H*), 7.55 (dd, $^3J_{H\text{-}H}$ = 7.88 Hz, $^3J_{H\text{-}H}$ = 7.85 Hz, 2H, Ph-*H*), 4.22 (s, 2H, C*H_2*). See Figure S10. Further analysis is in accordance with Pérez-Trujillo *et al.*[6]

3.3 ((4,5-Dibromoanthracen-9-yl)ethynyl)triisopropylsilane **2**

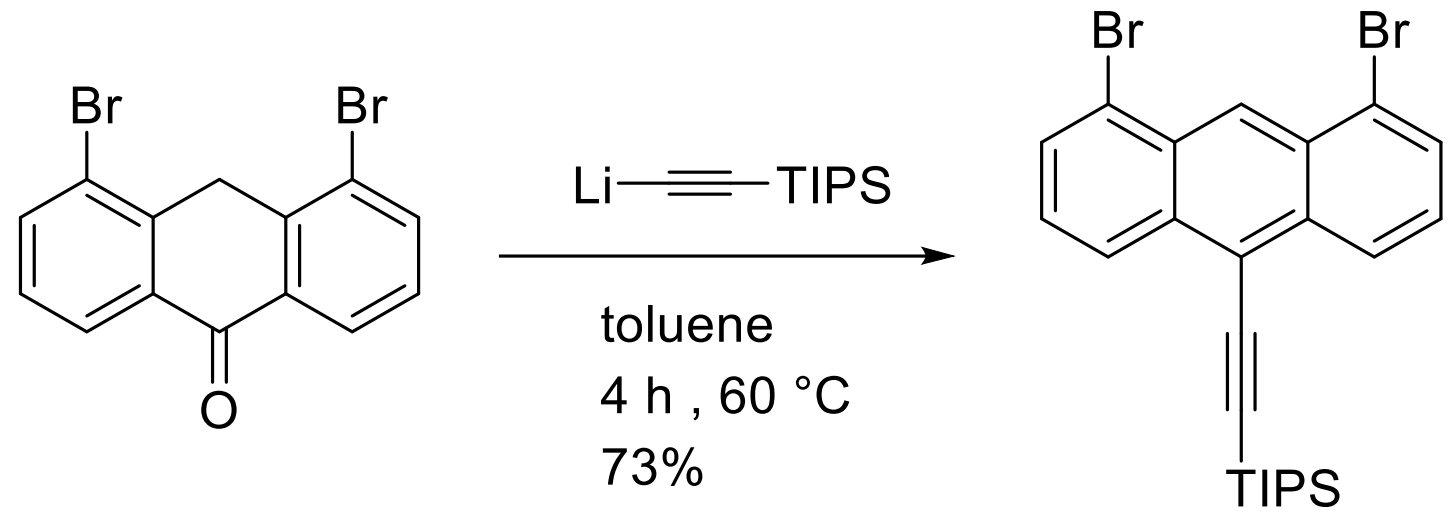

1 **2**

Under inert atmosphere, triisopropylsilylacetylene (27.4 mmol, 5.00 g, 6.15 mL 2.60 equiv.) was dissolved in toluene (125 mL). To this, a solution of *n*-butyllithium (2.5 M, 26.4 mmol, 10.5 mL, 2.50 equiv.) in Et_2O was added dropwise, and the solution was allowed to stir for 30 min. Afterwards, 1,8-dibromo-10-anthrone (10.5 mmol, 3.80 g, 1.00 equiv.) was added and the mixture was stirred for 4 h at 60 °C. The reaction was quenched with a saturated solution of NH_4Cl (10 mL) and extracted with $CHCl_3$ (3×100 mL). Column chromatography (SiO_2, petroleum ether), followed by two consecutive recrystallizations from petroleum ether and *i*PrOH yielded **2** (7.68 mmol, 3.97 g, 73%) as transparent, bright yellow crystals. **^{1}H NMR (400 MHz, $CDCl_3$):** δ/ppm = 9.25 (s, 1H, Ph-*H*), 8.61 (dt, $^3J_{H\text{-}H}$ = 8.66 Hz, $^4J_{H\text{-}H}$ = 1.03 Hz, 2H, Ph-*H*), 7.92 (dd, $^3J_{H\text{-}H}$ = 7.17 Hz, $^4J_{H\text{-}H}$ = 1.03 Hz, 2H, Ph-*H*), 7.55 (dd, $^3J_{H\text{-}H}$ = 8.66 Hz, $^3J_{H\text{-}H}$ = 7.17 Hz, 2H, Ph-*H*), 1.33 – 1.28 (m, 3H, TIPS-C*H*), 1.26 (d, $^3J_{H\text{-}H}$ = 5.81 Hz, 18H, TIPS-CH_3). See Figure S11. **^{13}C NMR (100 MHz, $CDCl_3$):** δ/ppm = 133.9, 130.1, 130.0, 127.4, 127.1, 126.6, 123.8, 119.4, 18.7, 11.3. See Figure S12. **HR MS (MALDI, DCTB, pos.):** *m/z* for $C_{25}H_{28}Br_2Si$ = 516.03027 ([M$^+$] found), 516.03066 (calculated). See Figure S13.

3.4 5-(8-(4-(9H-Carbazol-9-yl)phenyl)-10-((triisopropylsilyl)ethynyl)anthracen-1-yl)isophthalonitrile **3**

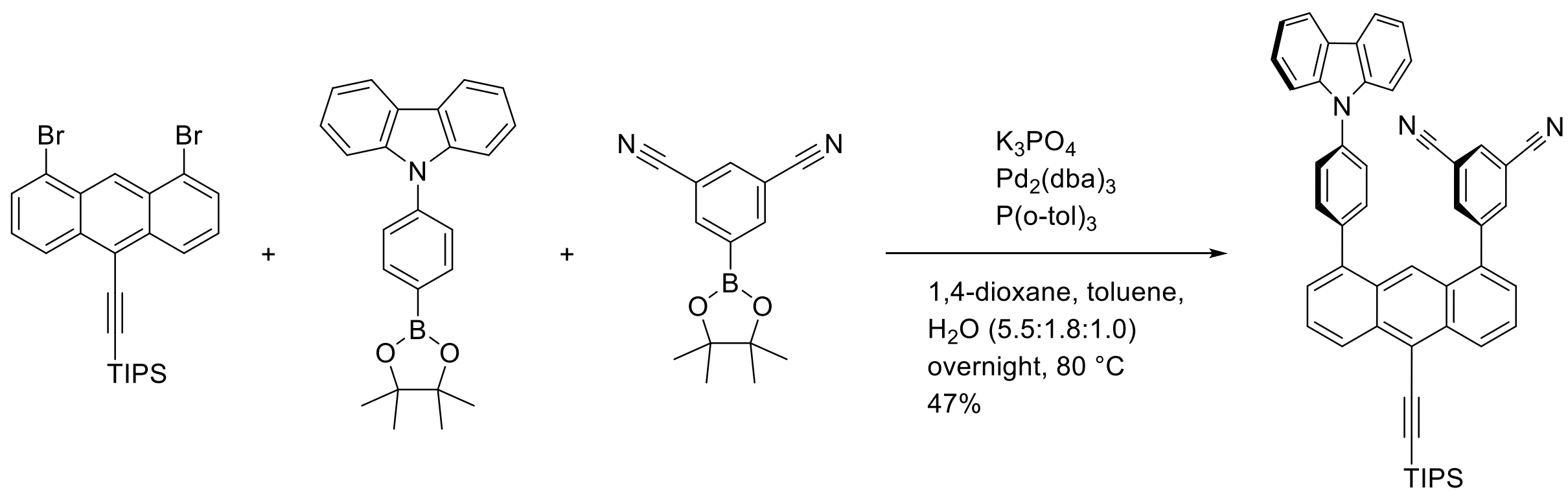

2 **3**

Under inert atmosphere, **2** (4.84 mmol, 2.50 g, 1.00 equiv.), 5-(4,4,5,5-Tetramethyl-1,3,2-dioxaborolan-2-yl)iso-phthalonitrile (5.33 mmol, 1.35 g, 1.10 equiv.), 9-(4-(4,4,5,5-tetramethyl-1,3,2-dioxaborolan-2-yl)phenyl)-9H-carbazole (5.33 mmol, 1.97 g, 1.10 equiv.), K_3PO_4 (9.68 mmol, 2.06 g, 2.00 equiv.), tris(*o*-tolyl)-phosphine (0.581 mmol, 177 mg, 0.12 equiv.) and $Pd_2(dba)_3$ (0.0726 mmol, 66.5 mg, 0.0150 equiv.) were dispersed in a mixture of 1,4-dioxane, toluene and H_2O (5.5:1.8:1.0, 250 mL) and the mixture was degassed. It was then heated to 80 °C and allowed to stir overnight. The reaction mixture was extracted with CH_2Cl_2 (3×100 mL), and column chromatography (SiO_2, petroleum ether:CH_2Cl_2 = 1:2) was performed to purify the product. Subsequent recrystallization from *i*PrOH provided **3** (2.29 mmol, 1,662 mg, 47%) as a bright yellow solid. **^{1}H NMR (400 MHz, $CDCl_3$):** δ/ppm = 8.78 (m, 2H, Ph-*H*), 8.27 (t, $^4J_{H\text{-}H}$ = 1.05 Hz, 1H, Ph-*H*), 8.21 (dt,

$^{3}J_{H\text{-}H}$ = 7.74 Hz, $^{4}J_{H\text{-}H}$ = 0.99 Hz, 2H, Ph-*H*), 8.01 (d, $^{4}J_{H\text{-}H}$ = 1.59 Hz, 2H, Ph-*H*), 7.80 (t, $^{4}J_{H\text{-}H}$ = 1.52 Hz, 1H, Ph-*H*), 7.78 – 7.61 (m, 8H, Ph-*H*), 7.52 (ddd, $^{3}J_{H\text{-}H}$ = 8.26 Hz, $^{3}J_{H\text{-}H}$ = 7.13 Hz, $^{4}J_{H\text{-}H}$ = 1.23 Hz, 2H, Ph-*H*), 7.43 (dd, $^{3}J_{H\text{-}H}$ = 6.73 Hz, $^{4}J_{H\text{-}H}$ = 1.16 Hz, 1H, Ph-*H*), 7.36 (m, 4H, Ph-*H*), 1.41 – 1.25 (m, 21H, TIPS-*H*). See Figure S14. **^{13}C NMR (100 MHz, CDCl$_3$):** δ/ppm = 143.6, 141.1, 140.1, 139.3, 137.6, 137.5, 136.1, 133.9, 133.5, 133.0, 131.5, 130.4, 128.8, 128.7, 127.6, 127.4, 127.3, 127.0, 126.4, 126.1, 123.7, 123.3, 120.7, 120.4, 119.4, 116.6, 114.3, 109.7, 104.7, 103.1, 19.1, 11.7. See Figure S15. **HR-MS (MALDI, DCTB, pos.):** *m/z* for $C_{51}H_{43}N_3Si$ = 725.32154 ([M$^+$] found), 725.32262 (calculated). See Figure S16.

3.5 5-(8-(4-(9H-Carbazol-9-yl)phenyl)-10-((triisopropylsilyl)ethynyl)anthracen-1-yl)isophthalonitrile *N*-(2-hydroxyethyl)-maleimide 9,10-Diels-Alder adduct **4**

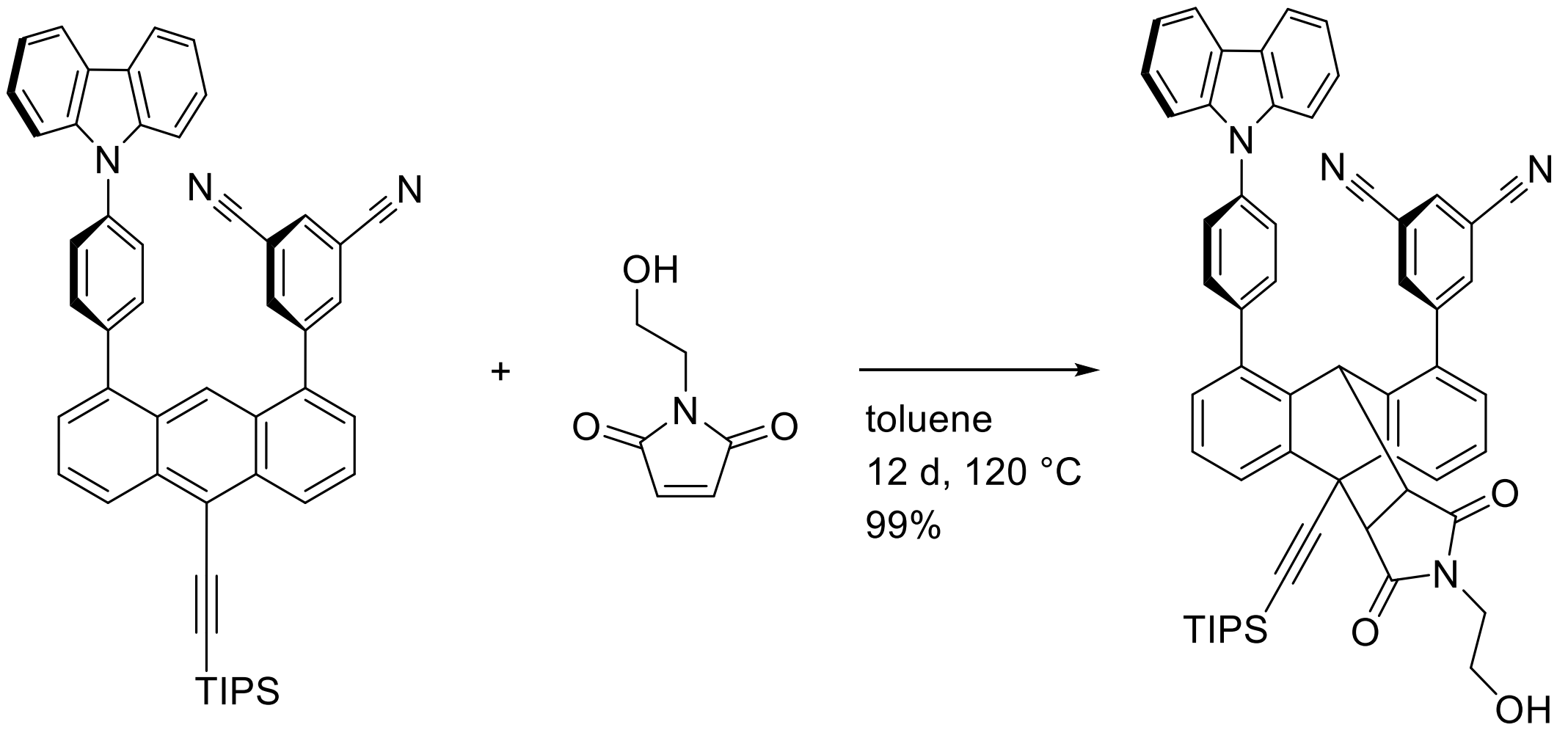

3 **4**

N-(2-hydroxyethyl)maleimide (3.89 mmol, 549 mg, 1.50 equiv.) and **3** (2.59 mmol, 1883 mg, 1.00 equiv.) were dissolved in toluene (125 mL), and the mixture was stirred for 7 d at 120 °C. The solvent was removed *in vacuo*, followed by column chromatography (SiO_2, petroleum ether:EtOAc = 2:1) to yield **4** (2.56 mmol, 2219 mg, 99%) as a white solid. **^{1}H NMR (400 MHz, CDCl$_3$):** δ/ppm = 8.20 (dt, $^{3}J_{H\text{-}H}$ = 7.76 Hz, $^{4}J_{H\text{-}H}$ = 0.99 Hz, 2H, Ph-*H*), 7.97 (m, 4H, Ph-*H*), 7.76 (t, $^{4}J_{H\text{-}H}$ = 1.54 Hz, 1H, Ph-*H*), 7.57 (ddd, , $^{3}J_{H\text{-}H}$ = 8.26 Hz, $^{3}J_{H\text{-}H}$ = 7.06 Hz, $^{4}J_{H\text{-}H}$ = 1.22 Hz, 2H, Ph-*H*), 7.47 – 7.34 (m, 9H, Ph-*H*), 7.32 (dd, $^{3}J_{H\text{-}H}$ = 7.75 Hz, $^{4}J_{H\text{-}H}$ = 1.26 Hz, 1H, Ph-*H*), 7.22 (d, $^{3}J_{H\text{-}H}$ = 7.86 Hz, 2H, Ph-*H*), 7.12 (dd, $^{3}J_{H\text{-}H}$ = 7.74 Hz, $^{4}J_{H\text{-}H}$ = 1.23 Hz, 1H, Ph-*H*), 5.15 (d, $^{3}J_{H\text{-}H}$ = 3.22 Hz, 1H, C*H*), 3.54 – 3.37 (m, 4H, CH_2, C*H*), 3.28 – 3.17 (m, 2H, CH_2), 1.37 – 1.28 (m, 21H, TIPS-*H*). See Figure S17. **^{13}C NMR (100 MHz, CDCl$_3$):** δ/ppm = 176.7, 174.0, 142.5, 141.8, 140.8, 139.9, 137.9, 137.5, 137.4, 137.0, 136.7, 135.8, 134.7, 134.2, 130.2, 129.2, 128.7, 127.6, 127.3, 127.2, 126.3, 125.7, 123.9, 123.8, 120.7, 120.5, 116.5, 114.2, 109.8, 101.2, 94.3, 60.4, 50.6, 48.2, 47.3, 41.5, 37.9, 19.0, 11.6. See Figure S18. **HR-MS (MALDI, DCTB, pos.):** *m/z* for $C_{57}H_{50}N_4O_3Si$ = 866.36476 ([M$^+$] found), 866.36522 (calculated). See Figure S19.

3.6 5-(8-(4-(9H-Carbazol-9-yl)phenyl)-10-ethynylanthracen-1-yl)isophthalonitrile *N*-(2-hydroxyethyl)maleimide 9,10-Diels-Alder adduct **5**

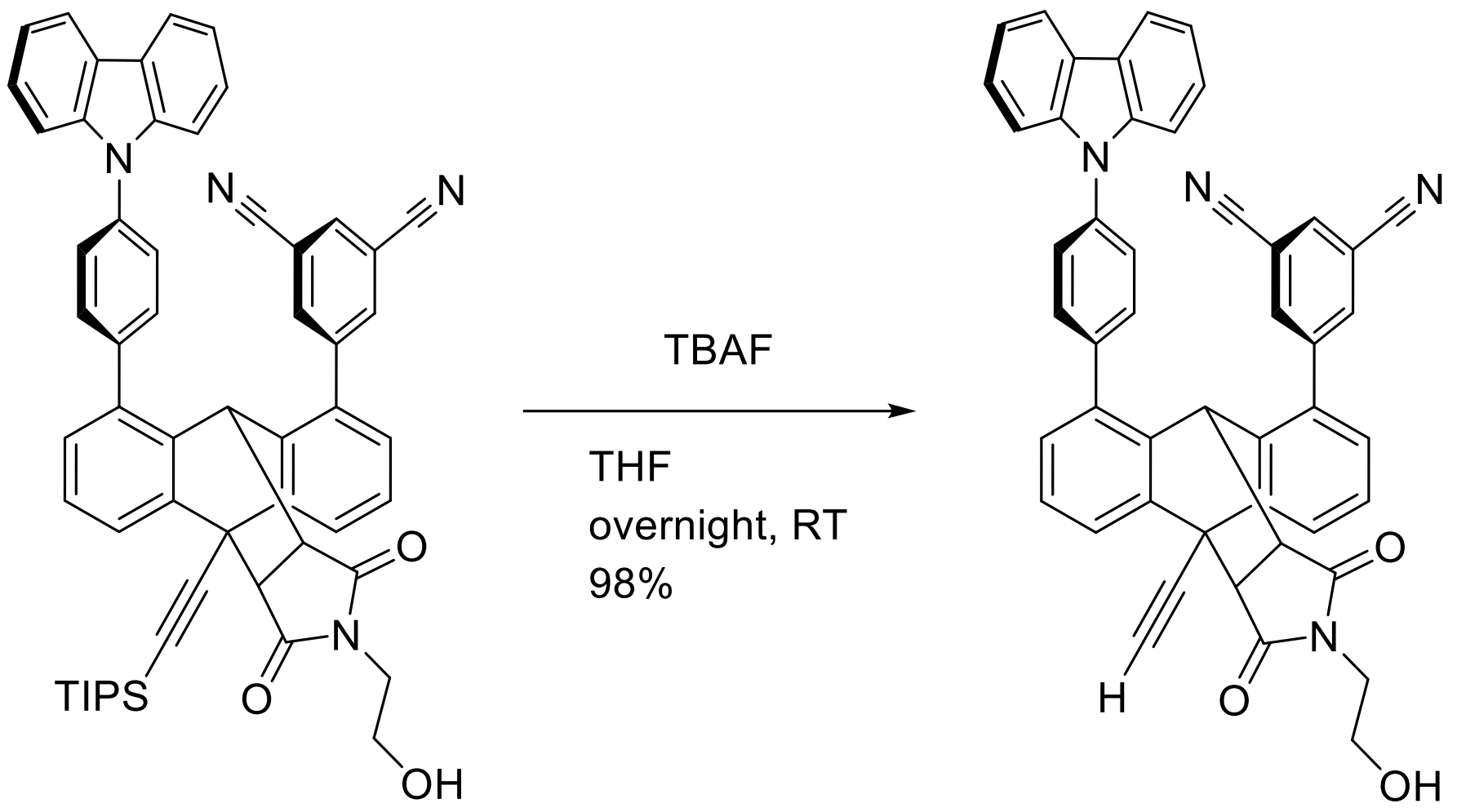

4 **5**

Diels-Alder adduct **4** (2.46 mmol, 2,130 mg, 1.00 equiv.) was dissolved in THF (70 mL), and a solution of TBAF (1 M, 5.00 mL) in THF was added. The reaction was stirred at room temperature overnight and subsequently quenched with a saturated solution of NH_4Cl (20 mL). The reaction was extracted with CH_2Cl_2 (3×25 mL), and concentrated under reduced pressure. Column chromatography (petroleum ether:THF = 1:1) afforded **5** (2.40 mmol, 1,707 mg, 98%) as a white powder. **^{1}H NMR (400 MHz, THF-d$_8$):** δ/ppm = 8.18 (dt, $^3J_{H\text{-}H}$ = 7.79 Hz, $^4J_{H\text{-}H}$ = 1.01 Hz, 2H, Ph-*H*), 8.10 (t, $^4J_{H\text{-}H}$ = 1.50 Hz, 1H, Ph-*H*), 7.93 (dd, $^3J_{H\text{-}H}$ = 7.53 Hz, $^4J_{H\text{-}H}$ = 1.27 Hz, 1H, Ph-*H*), 7.86 (dd, $^3J_{H\text{-}H}$ = 7.65 Hz, $^4J_{H\text{-}H}$ = 1.16 Hz, 1H, Ph-*H*), 7.55 (ddd, $^3J_{H\text{-}H}$ = 8.27 Hz, $^3J_{H\text{-}H}$ = 7.10 Hz, $^4J_{H\text{-}H}$ = 1.23 Hz, 2H, Ph-*H*), 7.49 (dd, $^3J_{H\text{-}H}$ = 7.21 Hz, $^4J_{H\text{-}H}$ = 1.29 Hz 2H, Ph-*H*), 7.43 (dt, $^3J_{H\text{-}H}$ = 8.27 Hz, $^4J_{H\text{-}H}$ = 0.87 Hz, 2H, Ph-*H*), 7.38 – 7.28 (m, 8H, Ph-*H*), 7.20 (dd, $^3J_{H\text{-}H}$ = 7.20 Hz, $^4J_{H\text{-}H}$ = 1.2 Hz, 1H, Ph-*H*), 5.18 (d, $^3J_{H\text{-}H}$ = 3.46 Hz, 1H, C*H*), 3.72 (s, 1H, acetylene-*H*), 3.50 – 3.43 (m, 2H, CH_2), 3.40 – 3.21 (m, 3H, CH_2, O*H*), 3.00 – 2.93 (m, 1H, C*H*), 2.88 – 2.79 (m, 1H, C*H*). See Figure S20. **^{13}C NMR (100 MHz, $CDCl_3$):** δ/ppm = 177.2, 174.2, 143.5, 143.1, 141.8, 140.4, 139.4, 138.6, 138.5, 138.3, 138.2, 136.9, 136.2, 135.6, 131.4, 129.9, 129.7, 128.0, 127.9, 127.6, 127.1, 125.9, 124.8, 124.1, 121.2, 121.1, 117.6, 115.1, 110.7, 81.9, 79.9, 59.3, 51.4, 48.2, 48.0, 41.5, 39.0. Figure S21. **HR-MS (MALDI, DCTB, pos.):** *m*/*z* for $C_{48}H_{30}N_4O_3$ = 710.23124 ([M$^+$] found), 710.23179 (calculated). See Figure S22.

3.7 5-(8-(4-(9H-Carbazol-9-yl)phenyl)-10-((4-(hydroxymethyl)phenyl)ethynyl)anthracen-1-yl)isophthalonitrile *N*-(2-hydroxyethyl)maleimide 9,10-Diels-Alder adduct **6a**

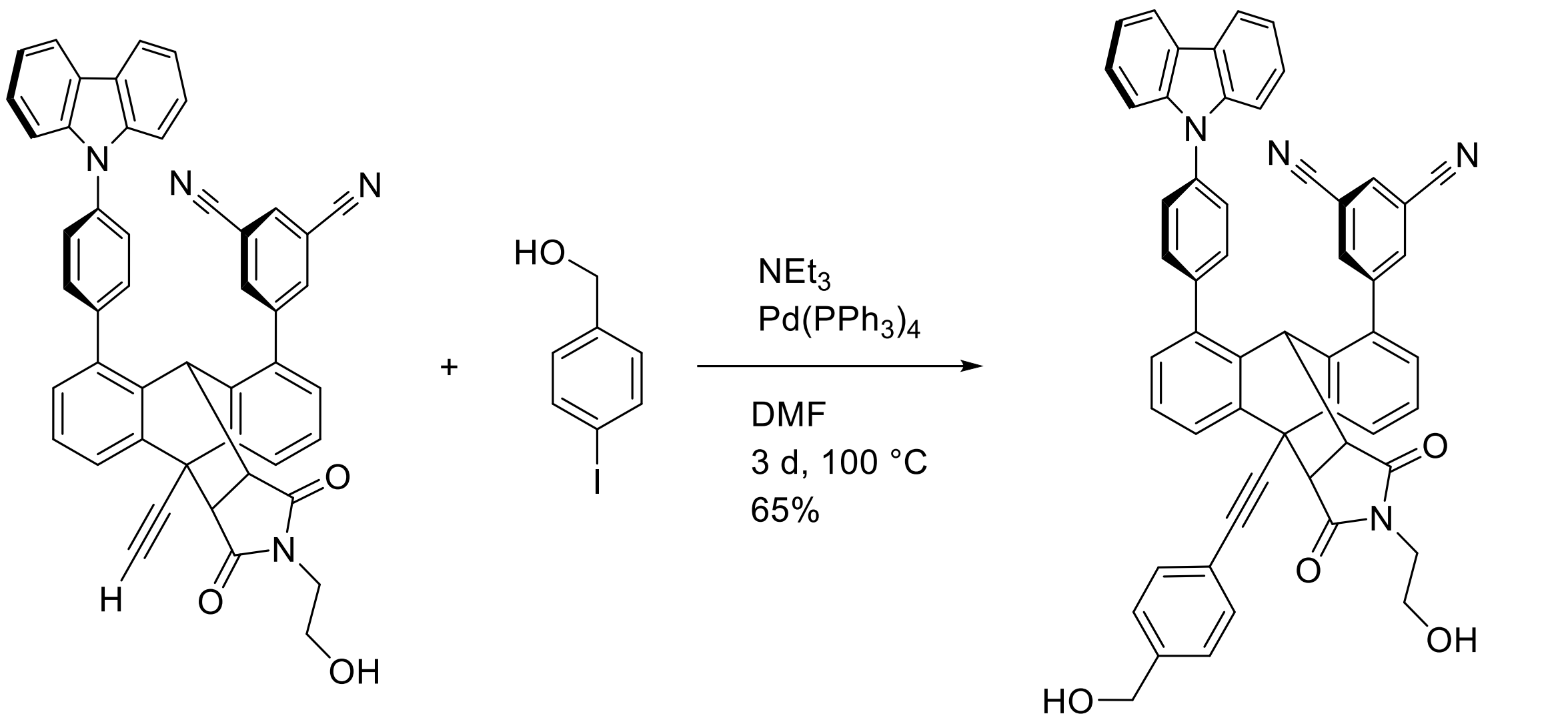

Under inert atmosphere, **5** (1.20 mmol, 850 mg, 1.00 equiv.), 4-iodobenzyl alcohol (1.79 mmol, 419 mg, 1.50 equiv.) and $Pd(PPh_3)_4$ (7.77 µmol, 8.98 mg, 0.0065 equiv.) were dispersed in dry DMF (40 mL). After degassing the mixture, anhydrous triethylamine (20 mL) was added, and the solution was stirred at 100 °C overnight. The mixture was extracted with CH_2Cl_2 (3×50 mL) and concentrated *in vacuo*. Column chromatography (SiO_2, petroleum ether:THF = 2:5) provided **6a** (0.777 mmol, 635 mg, 65%) as a white solid. **^{1}H NMR (400 MHz, THF-d$_8$):** δ/ppm = 8.19 (dt, $^3J_{H\text{-}H}$ = 7.84 Hz, $^4J_{H\text{-}H}$ = 0.97 Hz, 2H, Ph-*H*), 8.10 (t, $^4J_{H\text{-}H}$ = 1.50 Hz, 1H, Ph-*H*), 8.01 (dd, $^3J_{H\text{-}H}$ = 7.48 Hz, $^4J_{H\text{-}H}$ = 1.26 Hz, 1H, Ph-*H*), 7.92 (dd, $^3J_{H\text{-}H}$ = 7.58 Hz, $^4J_{H\text{-}H}$ = 1.11 Hz, 1H, Ph-*H*), 7.79 (m, 2H, Ph-*H*), 7.56 (ddd, $^3J_{H\text{-}H}$ = 8.22 Hz, $^3J_{H\text{-}H}$ = 6.97 Hz, $^4J_{H\text{-}H}$ = 1.21 Hz, 2H, Ph-*H*), 7.51 – 7.28 (m, 12H, Ph-*H*), 7.21 (dd, $^3J_{H\text{-}H}$ = 7.66 Hz, $^4J_{H\text{-}H}$ = 1.19 Hz, 1H, Ph-*H*), 5.22 (d, $^3J_{H\text{-}H}$ = 3.42 Hz, 1H, C*H*), 4.67 (d, $^3J_{H\text{-}H}$ = 5.83 Hz, 2H, C*H*$_2$), 4.35 (t, $^3J_{H\text{-}H}$ = 5.83 Hz, 1H, O*H*), 3.55 – 3.47 (m, 3H, C*H*$_2$, O*H*), 3.34 – 3.30 (m, 2H, C*H*$_2$), 3.02 – 2.94 (m, 1H, C*H*), 2.89 – 2.82 (m, 1H, C*H*). See Figure S23. **^{13}C NMR (100 MHz, CDCl$_3$):** δ/ppm = 177.3, 174.5, 145.1, 143.6, 141.8, 141.0, 139.4, 138.6, 138.6, 138.3, 138.2, 137.0, 136.2, 135.6, 132.9, 131.4, 129.8, 129.7, 128.0, 127.9, 127.6, 127.2, 127.1, 126.1, 124.8, 124.3, 122.4, 121.2, 121.1, 117.6, 115.1, 110.7, 93.1, 85.1, 64.6, 59.3, 51.8, 48.9, 48.1, 41.6, 39.0. See Figure S24. **HR-MS (MALDI, DCTB, pos.):** *m/z* for $C_{55}H_{36}N_4O_4$ = 816.27199 ([M$^+$] found), 816.27366 (calculated). See Figure S25.

3.8 5-(8-(4-(9H-Carbazol-9-yl)phenyl)-10-((4-(acetoxymethyl)phenyl)ethynyl)anthracen-1-yl)isophthalonitrile *N*-(2-acetoxyethyl)maleimide 9,10-Diels-Alder adduct **6b**

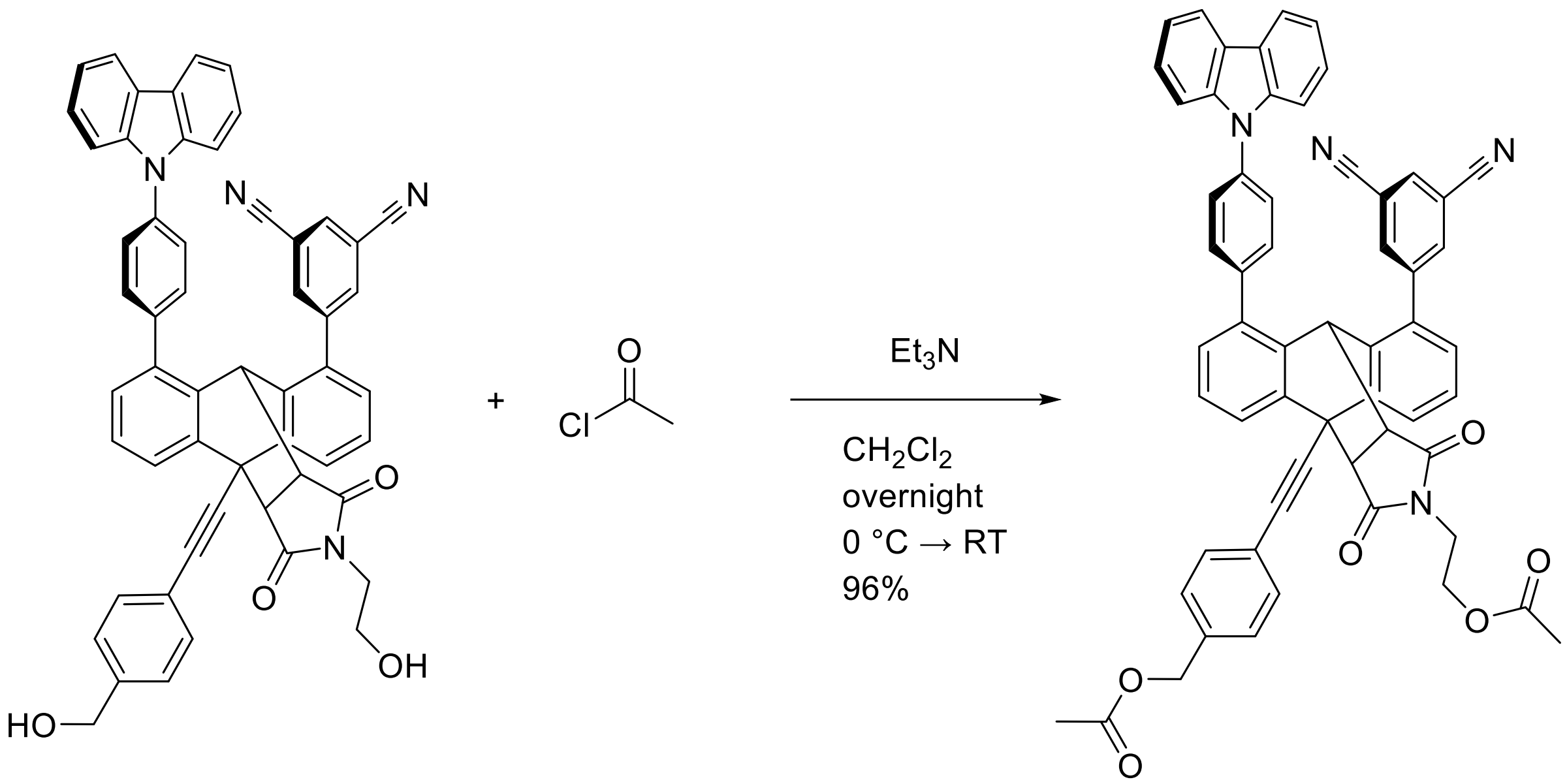

6a **6b**

Under inert atmosphere, compound **6a** (50 mg, 0.06 mmol, 1.00 equiv.) was dissolved in dry CH_2Cl_2 in a Schlenk tube and cooled to 0 °C. Triethylamine (0.03 mL, 0.18 mmol, 3.00 equiv.) and acetyl chloride (0.01 mL, 0.18 mmol, 3.00 equiv.) were added sequentially, and the reaction mixture was allowed to warm to room temperature and stirred overnight. The crude mixture was washed with water, and the organic phase was dried over $MgSO_4$ and concentrated under reduced pressure. Purification by column chromatography (SiO_2, CH_2Cl_2:EtOAc = 9:1) afforded the product **6b** as a white solid (53 mg, 0.06 mmol, 96%). **^{1}H NMR (400 MHz, $CDCl_3$):** δ/ppm = 8.20 (dt, *J* = 7.8, 1.0 Hz, 2H, Ph-*H*), 8.09 – 7.92 (m, 3H, Ph-*H*), 7.88 – 7.81 (m, 2H, Ph-*H*), 7.79 (t, *J* = 1.5 Hz, 1H, Ph-*H*), 7.62 – 7.52 (m, 2H, Ph-*H*), 7.51 – 7.33 (m, 10H, Ph-*H*), 7.30 (dd, *J* = 7.7, 1.2 Hz, 1H, Ph-*H*), 7.22 (d, *J* = 7.7 Hz, 2H, Ph-*H*), 7.12 (dd, *J* = 7.7, 1.2 Hz, 1H, Ph-*H*), 5.29 – 5.03 (m, 2H, C*H*$_2$), 3.77 – 3.61 (m, 2H, C*H*), 3.59 – 3.35 (m, 4H, C*H*$_2$), 2.16 (s, 3H, C*H*$_3$), 1.94 (s, 3H, C*H*$_3$). See Figure S26. **^{13}C NMR (101 MHz, $CDCl_3$):** δ/ppm = 175.8, 173.3, 170.6, 170.3, 142.2, 141.6, 140.4, 139.3, 137.5, 137.2, 136.8, 136.7, 136.3, 135.4, 134.1, 133.8, 132.2, 129.9, 128.9, 128.5, 128.1, 127.4, 126.9, 126.0, 125.9, 125.5, 123.4, 123.4, 122.2, 120.3, 120.2, 116.1, 114.2, 114.0, 109.4, 91.8, 83.9, 65.6, 60.7, 50.3, 47.5, 46.9, 37.5, 37.4, 20.8, 20.7, 14.0. See Figure S27. **FD-MS:** *m*/*z* for $C_{59}H_{40}N_4O_6$ = 900.29348 (found), 900.29479 (calculated). See Figure S28.

3.9 4-((4-(4-(9H-Carbazol-9-yl)phenyl)-5-(3,5-dicyanophenyl)-anthracen-9-yl)ethynyl)benzyl 2-bromo-2-methylpropa-noate *N*-[2-(*α*-isobutyr-yloxy)ethyl)maleimide 9,10-Diels-Alder adduct **6c**

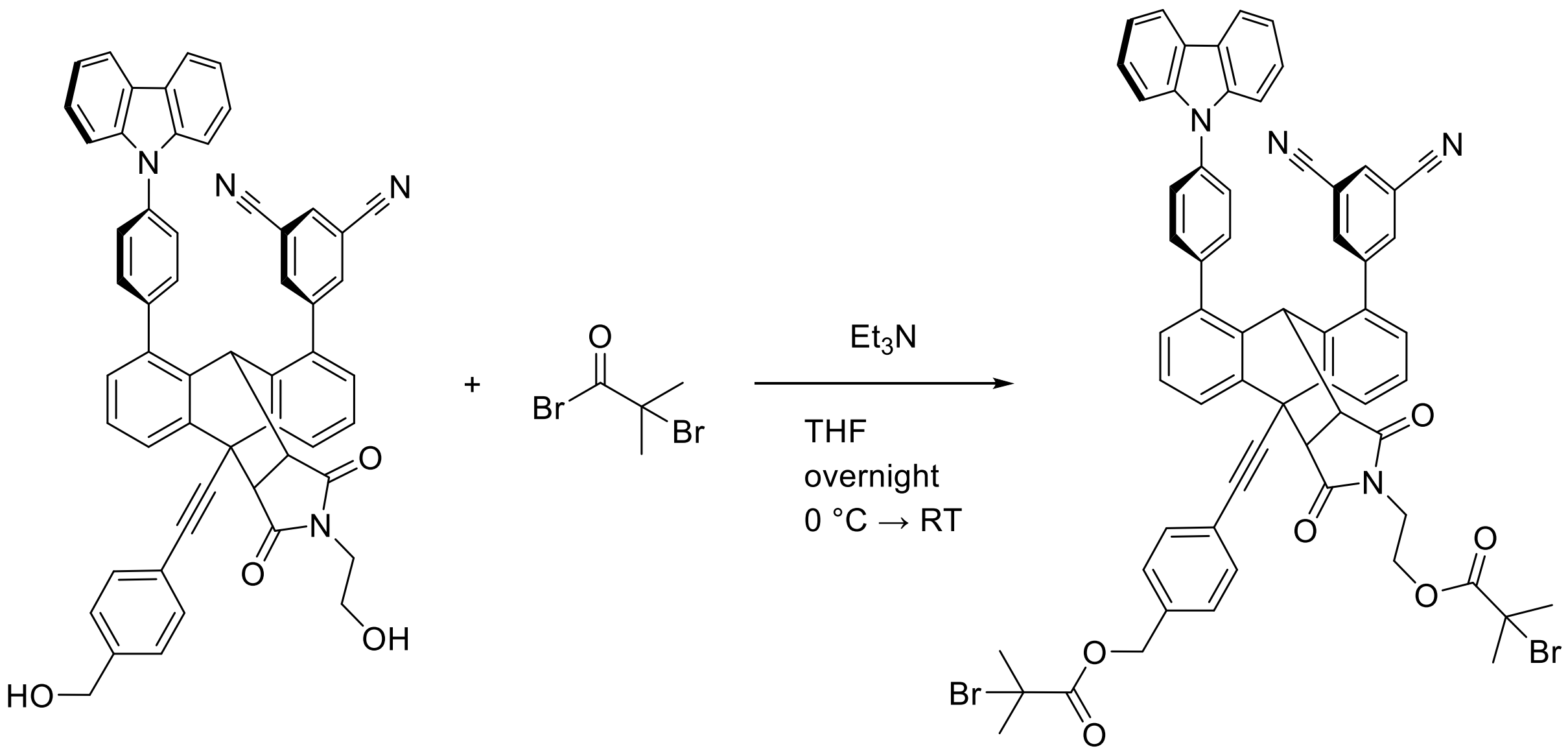

6a **6c**

Under inert atmosphere, **6** (0.686 mmol, 560 mg, 1.00 equiv.) was dissolved in dry THF (20 mL), and anhydrous triethylamine (5.48 mmol, 0.746 mL, 8.00 equiv.) was added. The solution was cooled to 0 °C and *α*-isobutyryl bromide (5.48 mmol, 0.674 mL, 8.00 equiv.) was added dropwise. The reaction was allowed to warm to room temperature and stirred overnight. Removal of the solvent *in vacuo* and Subsequent column chromatography (SiO_2, petroleum ether:THF = 3:1) afforded **6c** (0.413 mmol, 460 mg, 60%) as a colorless solid. **^{1}H NMR (400 MHz, $CDCl_3$):** *δ*/ppm = 8.20 (d, $^3J_{H\text{-}H}$ = 7.78 Hz, 2H, Ph-*H*), 7.98 (d, $^3J_{H\text{-}H}$ = 7.55 Hz, 1H, Ph-*H*), 7.95 (d, $^3J_{H\text{-}H}$ = 7.65 Hz, 1H, Ph-*H*), 7.84 (d, $^3J_{H\text{-}H}$ = 7.84 Hz, 2H, Ph-*H*), 7.79 (t, $^4J_{H\text{-}H}$ = 1.52 Hz, 1H, Ph-*H*), 7.58 (m, 2H, Ph-*H*), 7.51 (d, $^3J_{H\text{-}H}$ = 7.88 Hz, 2H, Ph-*H*), 7.41 (m, 9H, Ph-*H*), 7.31 (dd, $^3J_{H\text{-}H}$ = 7.78 Hz, $^4J_{H\text{-}H}$ = 1.14 Hz, 1H, Ph-*H*), 7.23 (d, $^3J_{H\text{-}H}$ = 7.61 Hz, 2H, Ph-*H*), 7.14 (dd, $^3J_{H\text{-}H}$ = 7.64 Hz, $^4J_{H\text{-}H}$ = 1.09 Hz, 1H, Ph-*H*), 5.30 (s, 2H, CH_2), 5.21 (d, $^3J_{H\text{-}H}$ = 3.41 Hz, 1H, C*H*), 3.93 (m, 1H, C*H*), 3.72 (m, 1H, C*H*), 3.51 (m, 4H, CH_2), 2.00 (s, 6H, CH_3), 1.89 (d, J = 1.82 Hz, 6H, CH_3). See Figure S29. **^{13}C NMR (101 MHz, $CDCl_3$):** *δ*/ppm = 176.04, 173.50, 171.59, 171.45, 142.55, 141.91, 140.78, 139.50, 137.83, 137.60, 137.56, 137.07, 136.54, 136.49, 135.72, 134.46, 134.18, 132.54, 130.21, 129.29, 128.94, 128.24, 127.85, 127.26, 126.36, 125.87, 123.80, 123.70, 122.70, 120.68, 120.53, 116.49, 114.30, 109.76, 92.17, 84.28, 67.24, 62.02, 55.71, 55.66, 50.72, 47.87, 47.31, 37.71, 37.36, 30.94, 30.84. See Figure S30. **HR-MS (MALDI, DCTB, pos.):** *m/z* for $C_{63}H_{46}Br_2N_4O_6$ = 1114.17443 ([M$^+$] found), 1114.17363 (calculated). See Figure S31.

3.10 5-(8-(4-(9H-Carbazol-9-yl)phenyl)-10-((4-(acryloyloxymethyl)phenyl)ethynyl)anthracen-1-yl)isophthalonitrile *N*-(2-acryloyloxyethyl)maleimide 9,10-Diels-Alder adduct **6d**

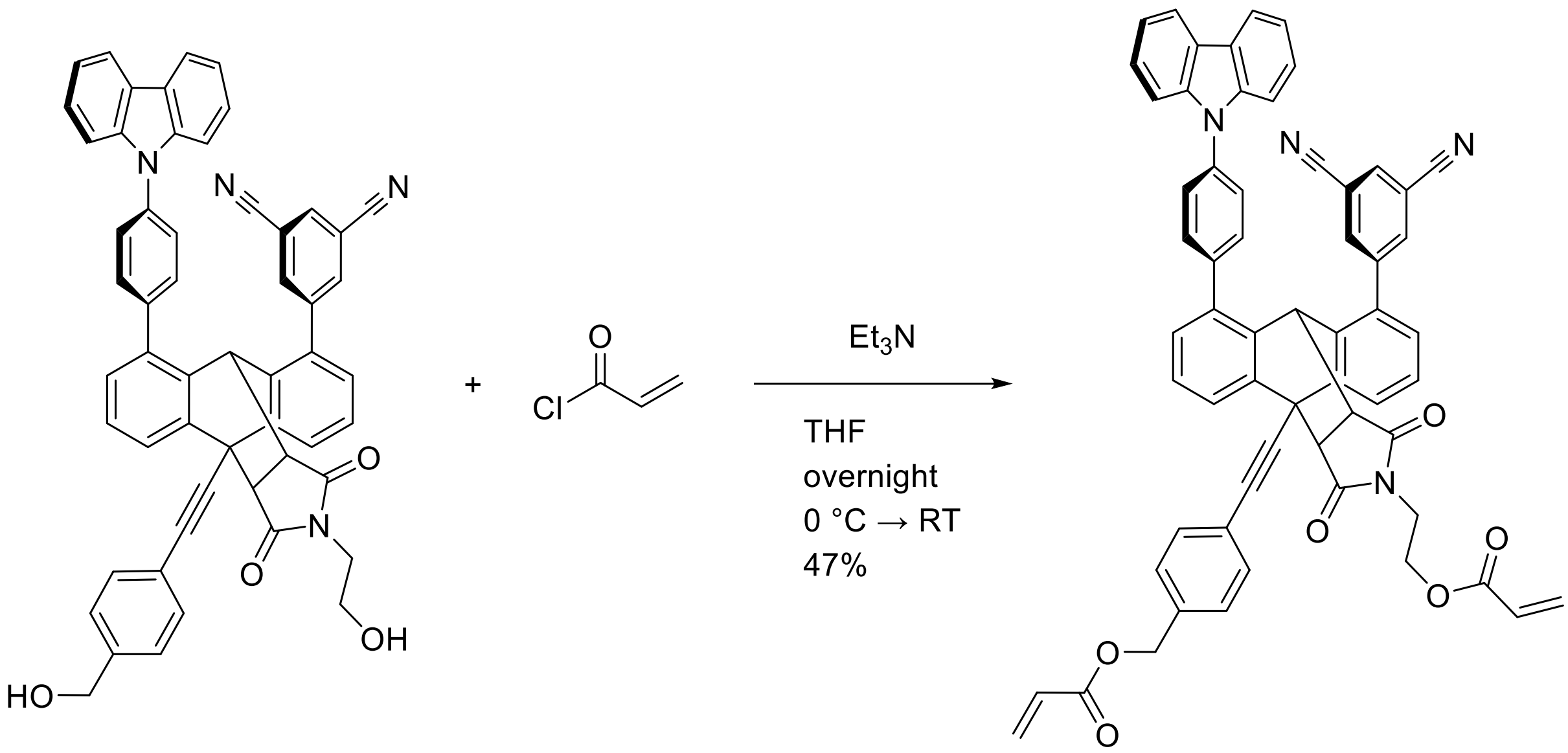

6a **6d**

Under inert atmosphere, compound **6a** (250 mg, 0.31 mmol, 1.00 equiv.) was dissolved in dry THF in a Schlenk tube and cooled to 0 °C. Triethylamine (0.17 mL, 1.22 mmol, 4.00 equiv.) and acryloyl chloride (0.10 mL, 1.22 mmol, 4.00 equiv.) were added whereby the HCl salt precipitated. The reaction mixture was allowed to warm to room temperature and stirred overnight. THF was removed *in vacuo*, the residual was dissolved in ethyl acetate and extracted with water and brine (3×100 mL H_2O, 2×100 mL brine). The organic phase was dried over $MgSO_4$ and concentrated under reduced pressure. Purification by column chromatography (SiO_2, CH_2Cl_2:EtOAc = 9:1) afforded the product **6d** as a white solid (134 mg, 0.15 mmol, 47%). **^{1}H NMR (400 MHz, $CDCl_3$):** δ/ppm = 8.20 (dt, *J* = 7.8, 1.1 Hz, 2H, Ph-*H*), 7.98 (dd, *J* = 7.6, 1.3 Hz, 2H, Ph-*H*), 7.92 (dd, *J* = 7.7, 1.2 Hz, 1H, Ph-*H*), 7.87 – 7.82 (m, 2H, Ph-*H*), 7.79 (t, *J* = 1.5 Hz, 1H, Ph-*H*), 7.63 – 7.54 (m, 2H, Ph-*H*), 7.52 – 7.48 (m, 2H, Ph-*H*), 7.47 – 7.28 (m, 9H, Ph-*H*), 7.22 (d, *J* = 7.8 Hz, 2H, Ph-*H*), 7.09 (dd, *J* = 7.7, 1.2 Hz, 1H, Ph-*H*), 6.50 (dd, *J* = 17.3, 1.5 Hz, 1H, CH=CH_2), 6.41 – 6.31 (m, 1H, CH=CH_2), 6.22 (dd, *J* = 17.3, 10.4 Hz, 1H, CH=CH_2), 6.01 – 5.88 (m, 2H, CH=CH_2), 5.77 (dd, *J* = 10.5, 1.4 Hz, 1H, CH=CH_2), 5.29 (s, 2H, CH_2-OH), 5.21 (d, *J* = 3.3 Hz, 1H, CH=CH_2), 3.73 (dh, *J* = 22.4, 5.5 Hz, 2H, C*H*), 3.60 (q, *J* = 4.8 Hz, 2H, CH_2), 3.51 – 3.41 (m, 2H, CH_2). See Figure S32. **^{13}C NMR (101 MHz, $CDCl_3$):** δ/ppm = 176.4, 173.8, 166.4, 166.0, 142.9, 142.3, 141.1, 139.8, 138.2, 137.9, 137.9, 137.4, 136.9, 136.5, 136.0, 134.7, 134.5, 132.8, 132.1, 131.8, 130.5, 129.6, 129.2, 128.8, 128.7, 128.3, 128.1, 127.6, 127.1, 126.7, 126.5, 126.1, 126.0, 124.1, 124.0, 122.9, 121.0, 120.8, 116.8, 114.6, 110.1, 92.5, 84.6, 66.3, 61.8, 51.0, 48.2, 47.5, 38.0, 38.0, 34.7, 30.8, 30.2. See Figure S33. **FD-MS:** *m/z* for $C_{61}H_{40}N_4O_6$ = 924.29446 (found), 924.29479 (calculated). See Figure S34.

3.11 5-(8-(4-(9H-Carbazol-9-yl)phenyl)-10-ethynylanthracen-1-yl)isophthalonitrile **7**

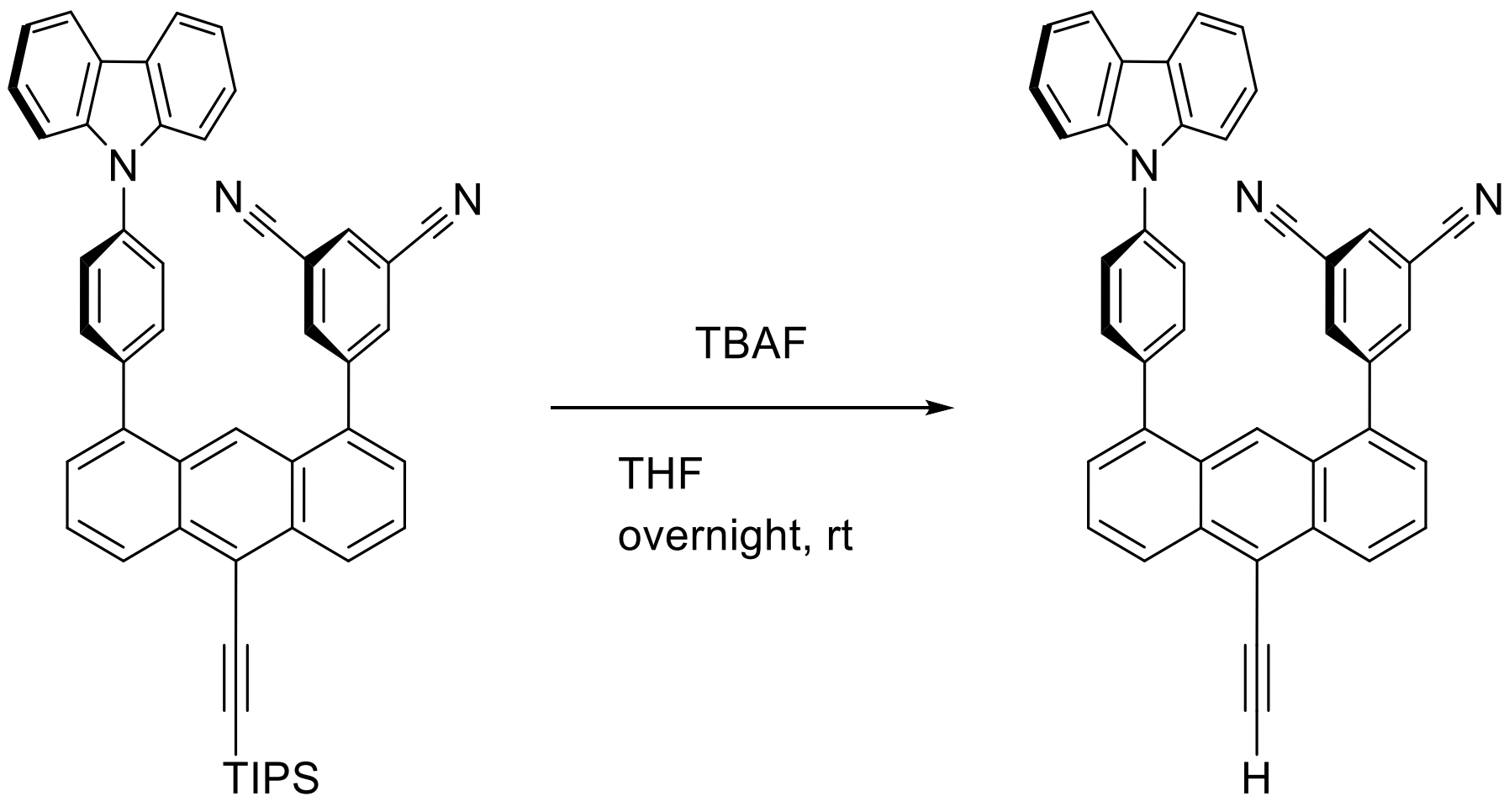

3 **7**

3 (0.344 mmol, 250 mg, 1.00 equiv.) was dissolved in THF (20 mL), and a solution of TBAF (1 M, 1 mL) was added. The mixture was stirred at room temperature overnight and then quenched with a saturated solution of NH_4Cl (2 mL). Extraction with CH_2Cl_2 (3×15 mL) and subsequent removal of the solvent *in vacuo* provided crude **7** (207 mg) as an orange solid, which was directly used without further purification in the next step. **HR-MS (MALDI, pos.):** *m/z* for $C_{42}H_{23}N_3$ = 569.18887 ([M^+] found), 569.18920 (calculated). See Figure S35.

3.12 4-((4-(4-(9H-Carbazol-9-yl)phenyl)-5-(3,5-dicyanophenyl)-anthracen-9-yl)ethynyl)benzyl acetate **8**

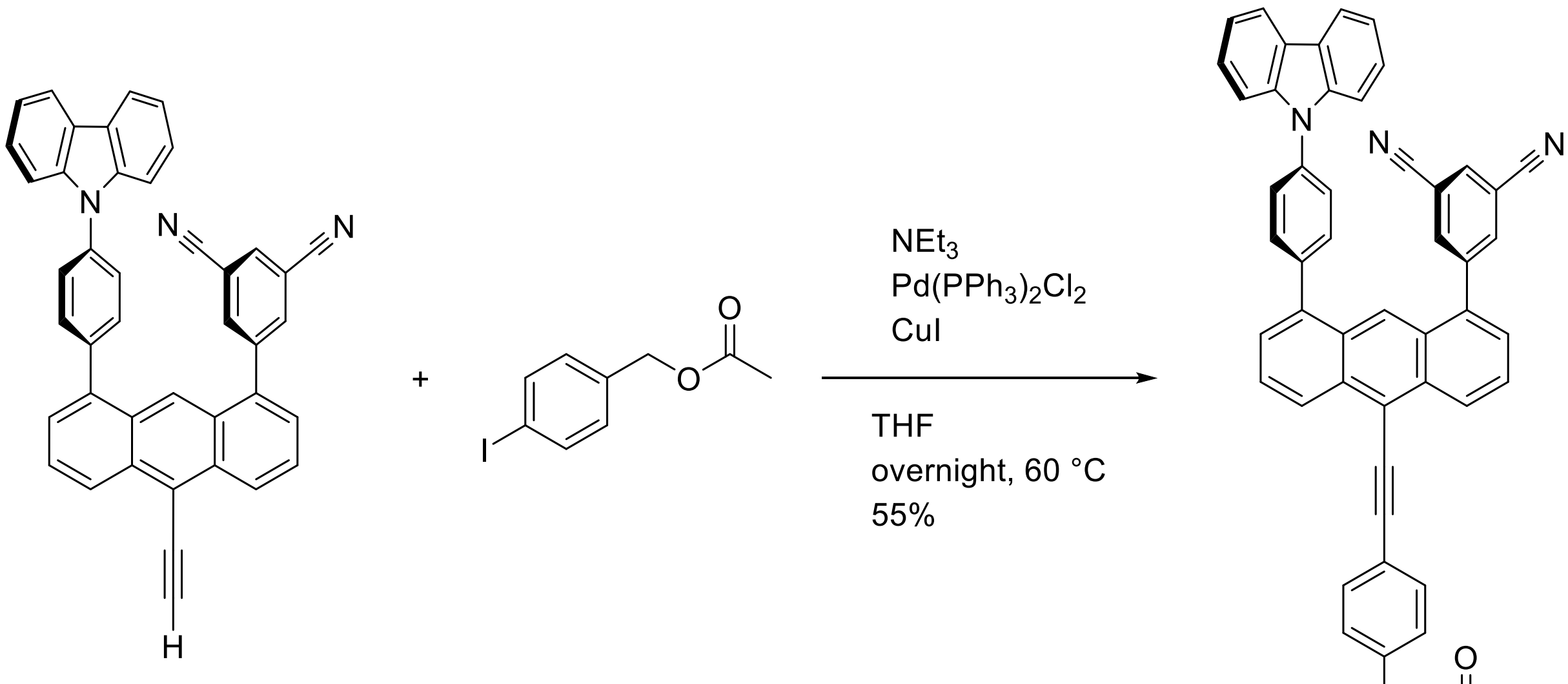

7 **8**

Under inert atmosphere, crude **7** (0.363 mmol, 207 mg, 1.00 equiv.), 4-iodobenzyl acetate (0.569 mmol, 157 mg, 1.56 equiv.), $Pd(PPh_3)_2Cl_2$ (18.2 µmol, 12.8 mg, 0.05 equiv.), and CuI (18.2 µmol, 3.46 mg, 0.05 equiv.) were dispersed in dry THF (25 mL), and the mixture was degassed. To this, anhydrous triethylamine (10 mL) was added, and the reaction was stirred at 60 °C overnight. Extraction with CH_2Cl_2 (3×25 mL), followed by column chromatography (SiO_2, petro-

leum ether:CH_2Cl_2 = 1:1) yielded **8** (0.189 mmol, 136 mg, 55% over two steps) as a yellow powder. **^{1}H-NMR (400 MHz, $CDCl_3$):** δ/ppm = 8.80 (m, 2H, Ph-*H*), 8.29 (s, 1H, Ph-*H*), 8.21 (d, $^3J_{H\text{-}H}$ = 7.74 Hz, 2H, Ph-*H*), 8.04 (d, $^4J_{H\text{-}H}$ = 1.55 Hz, 2H, Ph-*H*), 7.84 – 7.63 (m, 11H, Ph-*H*), 7.54 – 7.45 (m, 5H, Ph-H), 7.37 – 7.33 (m, 4H, Ph-*H*), 5.21 (s, 2H, CH_2), 2.17 (s, 3H, CH_3). See Figure S36. **^{13}C NMR (101 MHz, $CDCl_3$):** δ/ppm = 170.99, 143.51, 141.03, 140.18, 139.23, 137.59, 137.50, 136.95, 136.14, 133.88, 133.08, 132.60, 132.06, 131.50, 130.48, 128.77, 128.74, 128.57, 127.69, 127.50, 127.29, 127.27, 126.94, 126.34, 126.13, 123.72, 123.50, 123.26, 120.68, 120.36, 118.98, 116.55, 114.34, 109.71, 101.55, 86.58, 65.99, 21.19. See Figure S37. **HR-MS (MALDI, DCTB, pos.):** *m*/*z* for $C_{51}H_{31}N_3O_2$ = 717.24223 ([M^+] found), 717.24163 (calculated). See Figure S38.

4 Polymerizations

4.1 Linear Polymers

Prior to use, methyl acrylate was destabilized by passing through a short plug of neutral aluminum oxide. Copper wire was activated by immersion in concentrated HCl for 20 min, followed by washing with acetone and drying *in vacuo*. The activated wire was wrapped around a magnetic stir bar and stored under argon until use. Stock solutions of $CuBr_2$ in DMSO (3 mg per 3 mL) and Me_6TREN in DMSO (10 µL per 3 mL) were prepared prior to the polymerization.

Mechanophore **6c** (28 mg, 0.025 mmol, 1.00 equiv.), $CuBr_2$ (0.28 mg, 0.0013 mmol, 0.05 equiv.), Me_6TREN (1 µL, 0.0038 mmol, 0.15 equiv.), and destabilized methyl acrylate (2.266 mL, 25 mmol, 1,000 equiv.) were added to a Schlenk tube. DMSO (1.69 mL) was added such that the total volume ratio of methyl acrylate to DMSO was 1:1. The solution was degassed by three freeze-pump-thaw cycles. The copper wire-wrapped stir bar was then introduced into the Schlenk tube and fixed to the inner wall using an external magnet, and two additional freeze-pump-thaw cycles were performed. The polymerization was initiated by releasing the stir bar into the solution upon thawing. After 5.5 h, the reaction was quenched by exposure to air, and THF was added to reduce the viscosity of the mixture. The solution was passed through a plug of neutral aluminum oxide to remove the copper catalyst, and the solvent was removed under reduced pressure. The polymer was obtained after precipitation into cold MeOH (Y = 72%, M_n = 83.1 kDa, $Đ_M$ = 1.06).

4.2 Polymer Networks

4.2.1 PHA Networks

HA and tetraethylene glycol diacrylate (TEGDA) were passed through a short plug of neutral aluminum oxide prior to use to remove the stabilizer. Mechanophore crosslinker **6d** and the photoinitiator I2959 (0.2 mol%) were dissolved in THF. Destabilized hexyl acrylate and TEGDA were subsequently added, and the solution was degassed for 1 min by bubbling with Ar. The total solvent content was kept at 33 vol%, the total crosslinker percentage was 2 mol% of which 1 mol% consisted of mechanophore crosslinker. The degassed solution was transferred into a PTFE mold and irradiated at 365 nm for 25 min to initiate free radical photopolymerization. The resulting polymer network was removed from the mold and washed with Et_2O to remove unreacted monomers and residual solvent.

4.2.2 POEGMEA Networks

OEGMEA (M_n = 480 g mol^{-1}) and tetraethylene glycol diacrylate (TEGDA) were passed through a short plug of neutral aluminum oxide prior to use to remove the stabilizer. Mechanophore crosslinker **6d** and the photoinitiator I2959 (0.2 mol%) were dissolved in DMSO. Destabilized OEGMEA and TEGDA were subsequently added, and the solution was degassed for 1 min by bubbling with Ar. The total solvent content was kept at 33 vol%, the total crosslinker percentage was 2 mol% of which 1 mol% consisted of mechanophore crosslinker. The degassed solution was transferred into a PTFE mold and irradiated at 365 nm for 25 min to initiate free radical photopolymerization. The resulting polymer network was removed from the mold and washed with DI water to remove unreacted monomers and residual solvent.

5 Supporting Analytical Data

5.1 Dynamic Scanning Calorimetry

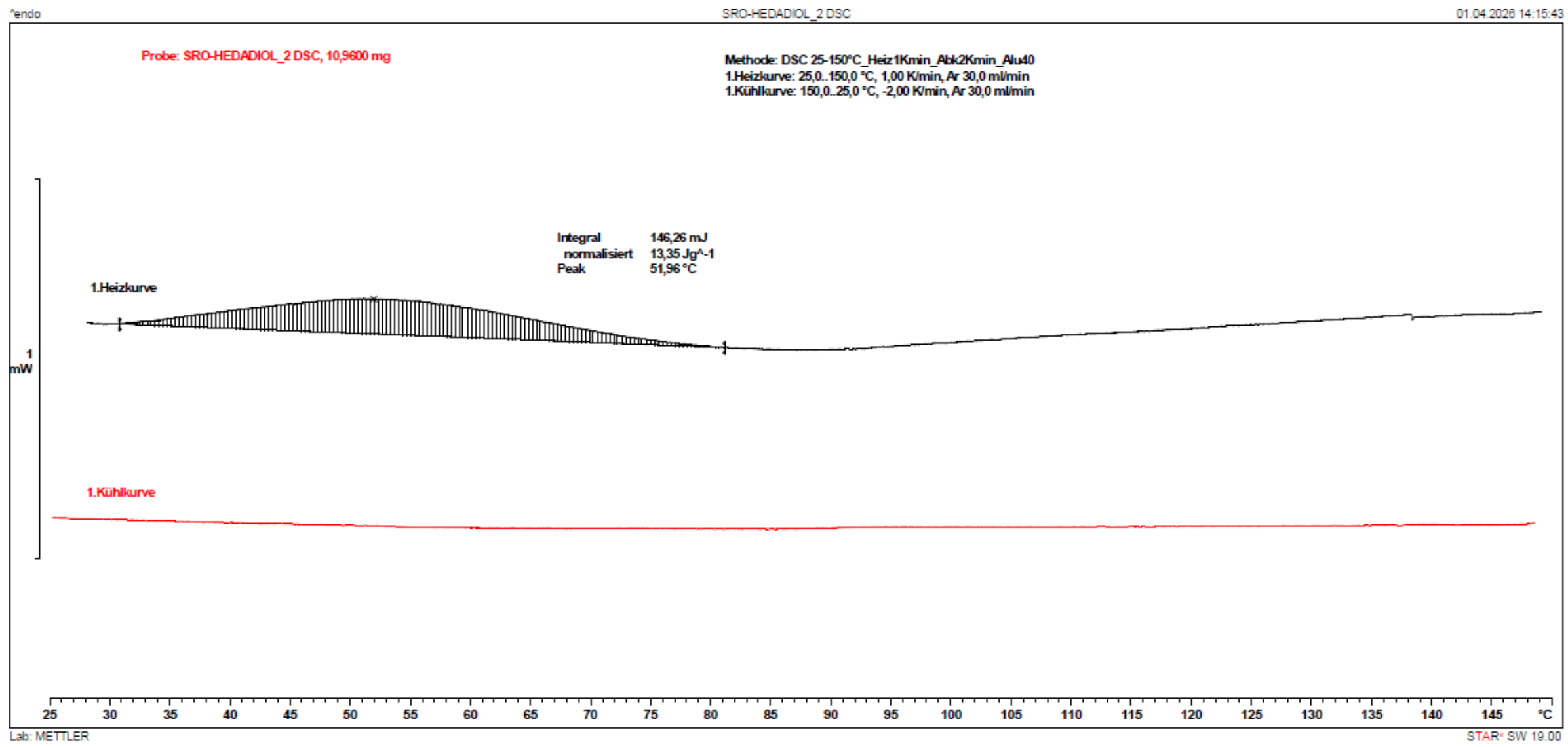

Figure S1. DSC trace of **6a** (heating rate: 1.00 K min^{-1}).

5.2 Variable-Temperature Nuclear Magnetic Resonance Spectroscopy

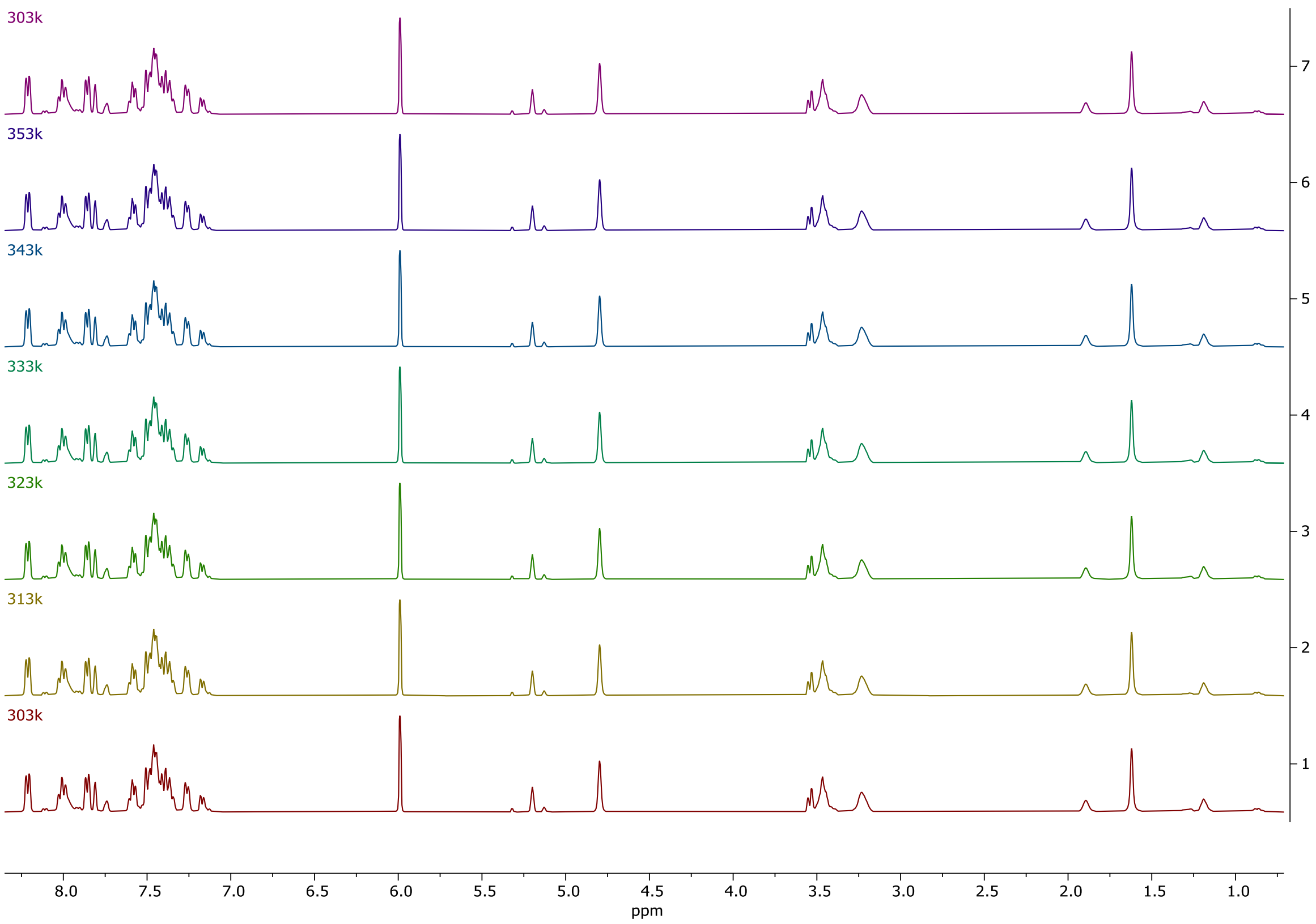

Figure S2. ^{1}H NMR spectra of compound **6a** in tetrachloroethane-*d*2 recorded during a variable-temperature experiment. Spectra were acquired starting at 303 K and measured in 10 K increments up to 353 K, followed by a final spectrum at 303 K after cooling. The spectra do not indicate any structural changes during the heating process.

5.3 Heating Experiment with AIBN

To investigate the behavior of mechanophore **6a** in the presence of radicals and heat, **6a** (15 mg, 0.02 mmol, 1 equiv.) and AIBN (15 mg, 0.10 mmol, 5 equiv.) were dissolved in MeCN (3 mL) and heated to 60 °C for a total of 20 h. Samples were taken after 2, 4, 6, and 20 h and analyzed by UV-Vis and fluorescence spectroscopy in MeCN (Figure S3). The fluorescence spectra show a new emerging emission band with $\lambda_{em,max}$ = 436 and 462 nm.

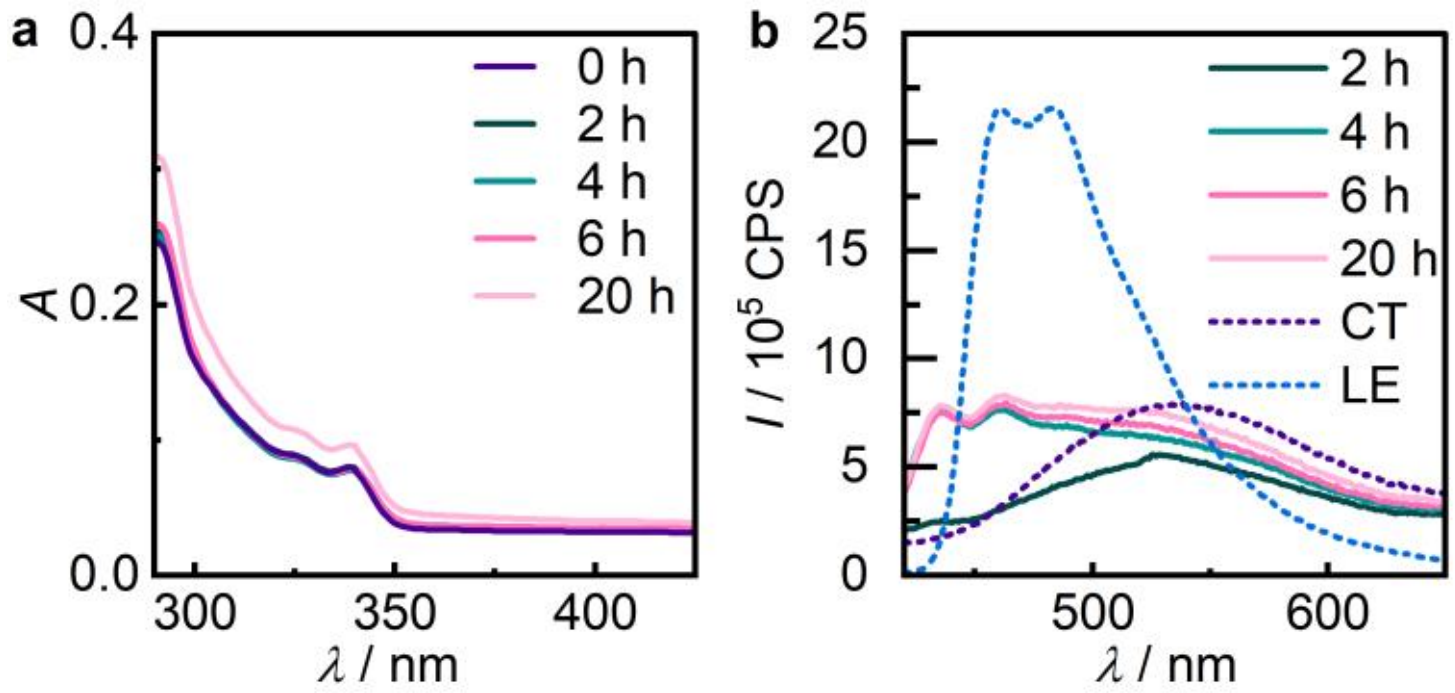

Figure S3. (a) UV Vis spectra of **6a** before, during and after the heating experiment with AIBN in MeCN. (b) Fluorescence spectra of heating experiment of **6a** with AIBN, recorded in MeCN. (λ_{exc} = 350 nm). For better comparison, CT (λ_{exc} = 350 nm). and LE ((λ_{exc} = 400 nm) emission spectra of the small molecules **6b** and **8**, respectively, in MeCN were additionally plotted.

5.4 Calculated Ground State Energies

Table S1. Ground state energies of **8** and **6b**, calculated by TD-DFT.

	**8** (MeCN)	**6b** (MeCN)	**6b** ($CHCl_3$)	**6b** (Toluene)
T_1	1.54 eV	3.25 eV	3.24 eV	3.24 eV
S_1	2.82 eV	3.64 eV	3.59 eV	3.57 eV
S_2	3.60 eV	3.93 eV	3.92 eV	3.91 eV

5.5 Lifetime Measurements

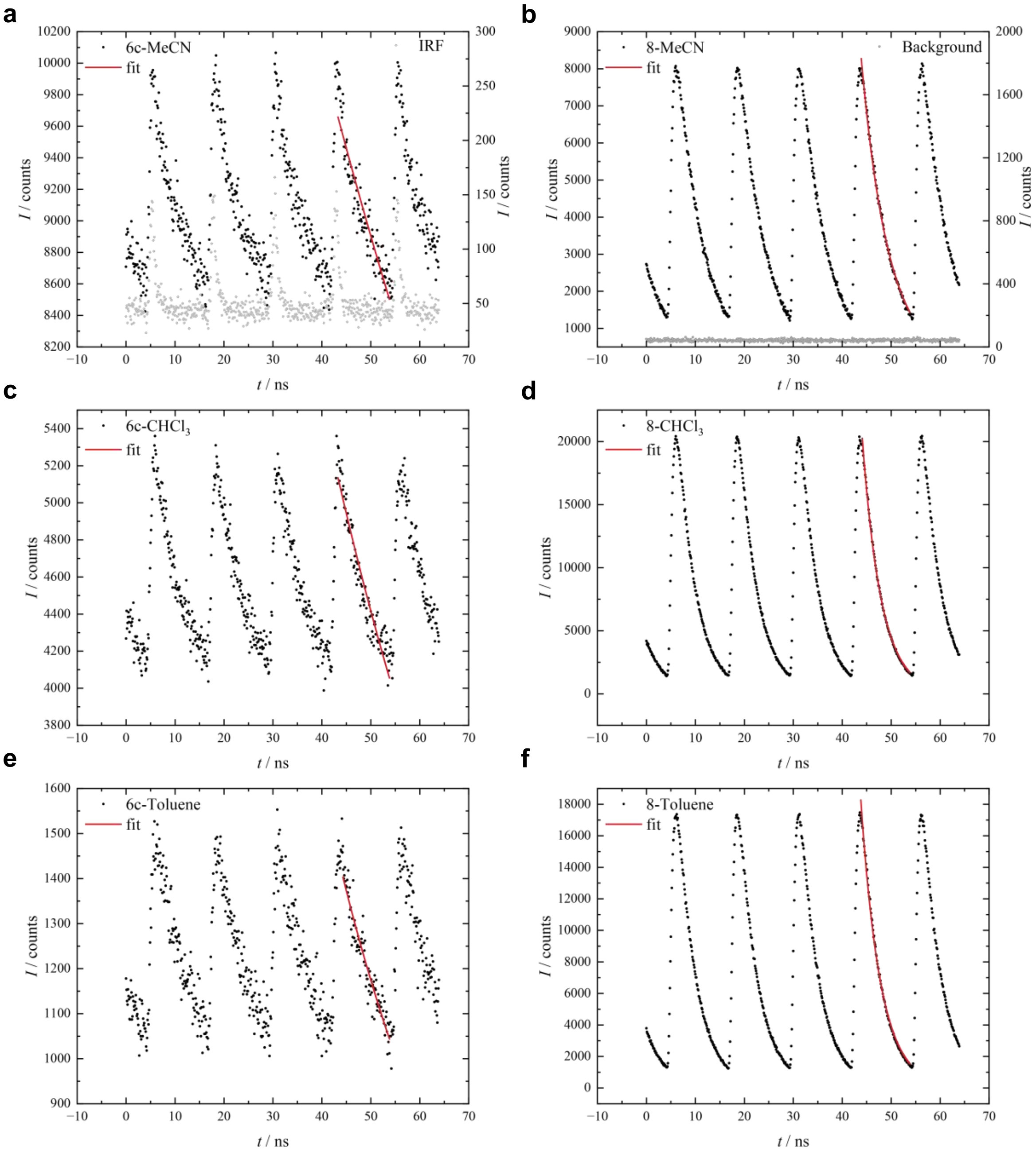

Figure S4. Fluorescence decay data comparing the deactivated **6c** (a,c,e) and activated **8** (b,d,f) model compounds. From the top down the data is shown in (a,b) MeCN, (c,d) $CHCl_3$, and (e,f) toluene respectively. Photons were counted in a 64 ns window, which included 5 pulses of the laser. As an example for all decay experiments the instrument response function (IRF) is shown in (a) and (b) shows the background counts (both in gray color).

5.6 Control Sonication of 6a

To verify that small molecule control compound **6a** cannot be activated under sonication, control experiments were performed. 20 mg of **6a** were dissolved in 20 mL of THF, and the solution was degassed prior to sonication. Ultrasonication was carried out for 4 h using a pulsed sequence of 1 s on/1 s off at 30% maximum amplitude. UV-Vis absorption and fluorescence spectra were recorded before and after sonication to assess any structural or photophysical changes.

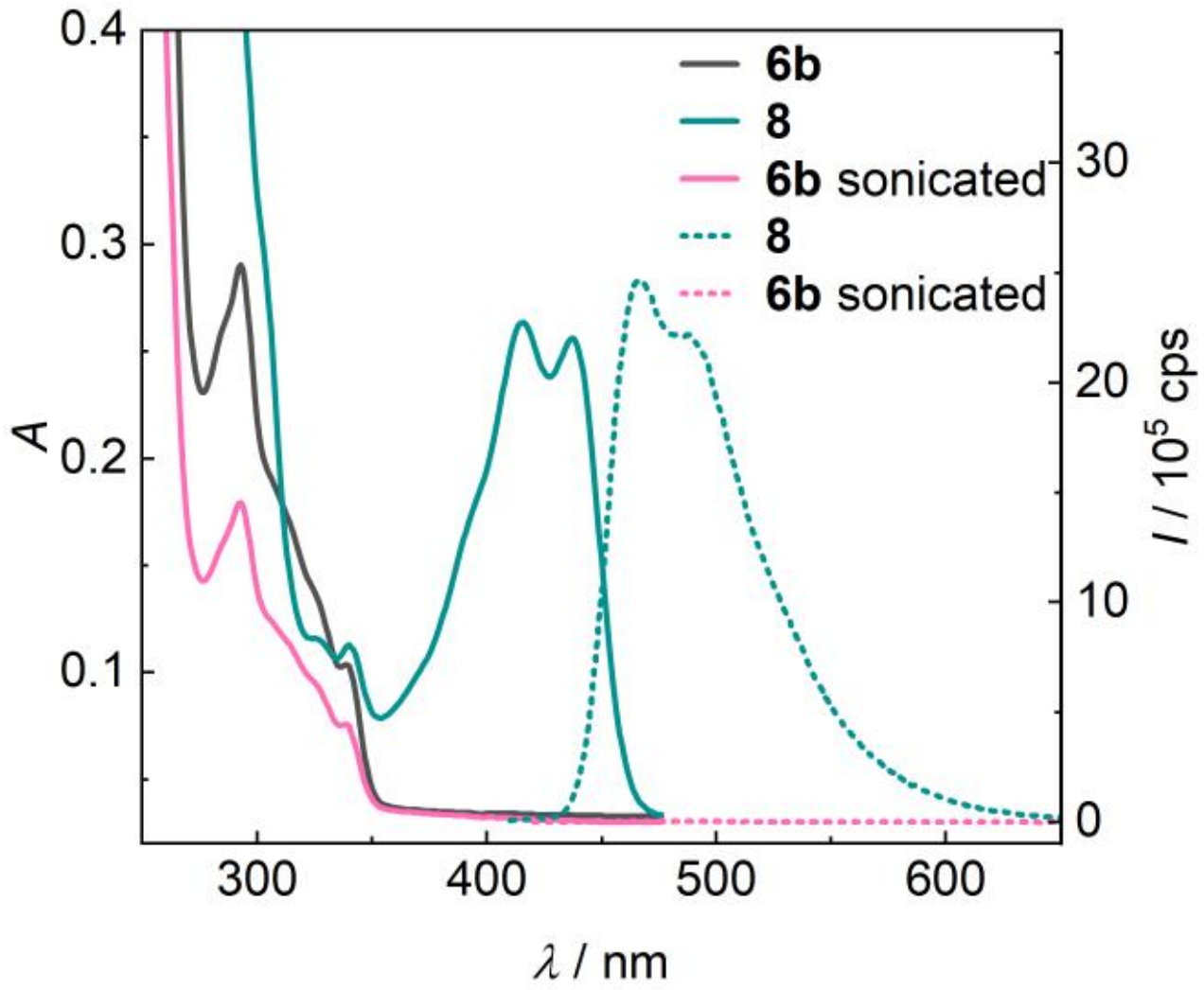

Figure S5. UV-Vis and emission spectra of **8** and **6b** before and after sonication (λ_{exc} = 400 nm). All spectra were recorded in $CHCl_3$.

5.7 Control Polymer Networks

To confirm that the fluorescence signal observed in networks containing mechanophore **6d** originates exclusively from the mechanophore and not from scattering artifacts or other false-positive contributions, control networks were prepared using solely TEDGA as the crosslinker (2 mol%) and examined by DinoLite fluorescence microscopy under identical imaging conditions.

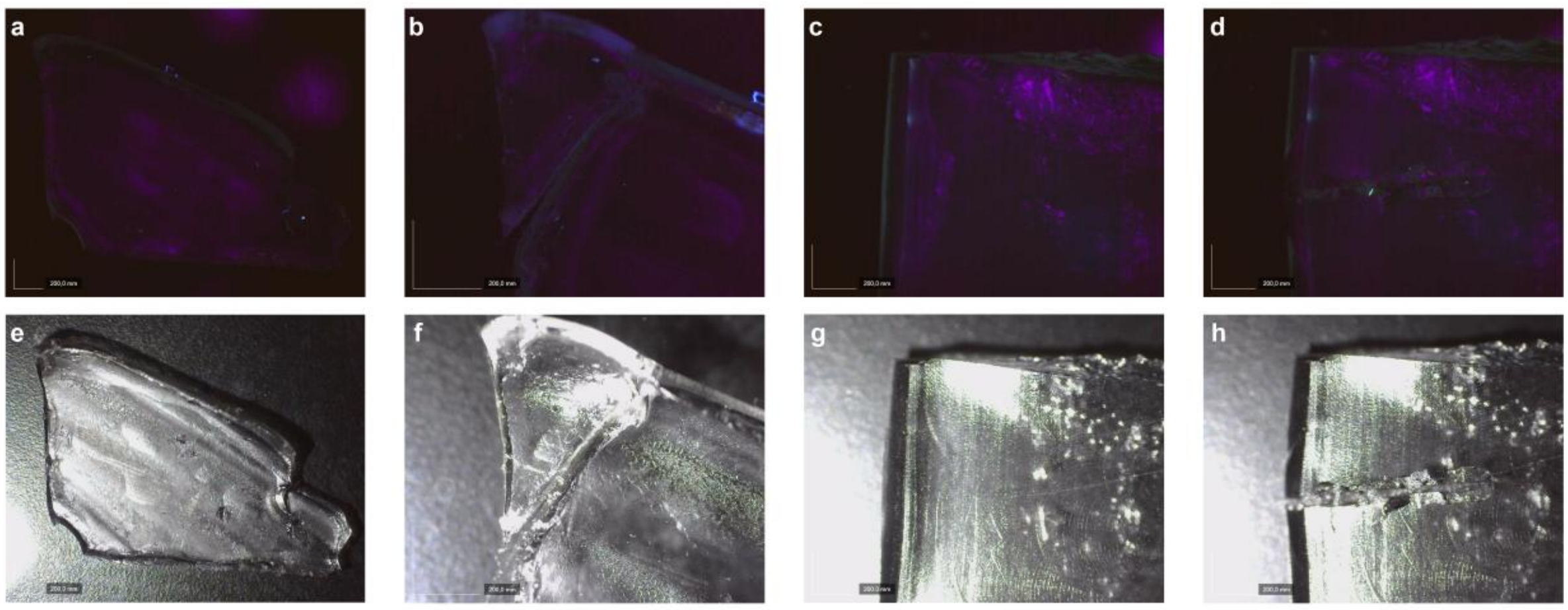

Figure S6. (a) Fluorescence micrograph (λ_{exc} = 400 nm) of pristine hexyl acrylate network with 2 mol% TEGDA crosslinker. (b) Fluorescence micrograph (λ_{exc} = 400 nm) of cut hexyl acrylate network with 2 mol% TEGDA crosslinker. (c) Fluorescence micrograph (λ_{exc} = 400 nm) of pristine POEGMEA network with 2 mol% TEGDA crosslinker. (d) Fluorescence micrograph (λ_{exc} = 400 nm) of cut POEGMEA network with 2 mol% TEGDA crosslinker. (e) Brightfield picture of pristine hexyl acrylate network with 2 mol% TEGDA crosslinker. (f) Brightfield picture of cut hexyl acrylate network with 2 mol% TEGDA crosslinker. (g) Brightfield picture of pristine POEGMEA network with 2 mol% TEGDA crosslinker. (h) Brightfield picture of cut POEGMEA network with 2 mol% TEGDA crosslinker.

5.8 Mechanochemical Selectivity

To determine the mechanochemical selectivity, ^{1}H NMR and GPC samples before and after 4 h of sonication were analyzed. The fraction of total scission was determined from GPC (Figure S7) following equation:

$$\text{fraction of total scission} = \frac{M_{\text{n,end}}^{-1} - M_{\text{n,start}}^{-1}}{M_{\text{n,start}}^{-1}} \times 100\% = 82\%$$

From the ^{1}H NMR before and after sonication (Figure S8), the fraction of cleaved mechanophore was obtained by analyzing the integrals of the 9H proton of the Diels-Alder adduct (5.16 ppm) and the respective activated anthracene (8.28 ppm):

$$\text{fraction of cleaved mechanophore} = \frac{\int H_{\text{9H,active}}}{\int H_{\text{9H,Diels-Alder}} + \int H_{\text{9H,active}}} \times 100\% = 58\%$$

The selectivity of the mechanophore scission can then be obtained from both the fraction of cleaved mechanophore and the fraction of total scission by following equation:

$$\text{selective scission} = \frac{\text{fraction of cleaved mechanophore}}{\text{fraction of total scission}} \times 100\% = 71\%$$

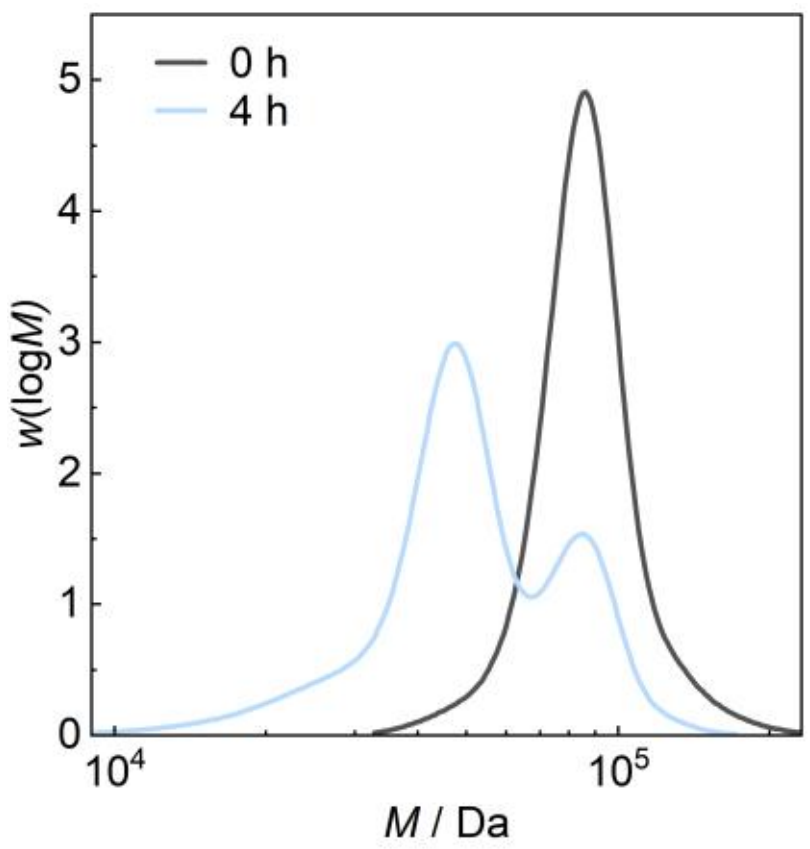

Figure S7. GPC MMDs (refractive index detector) of PMA-functionalized OFP before and after 4 h of sonication.

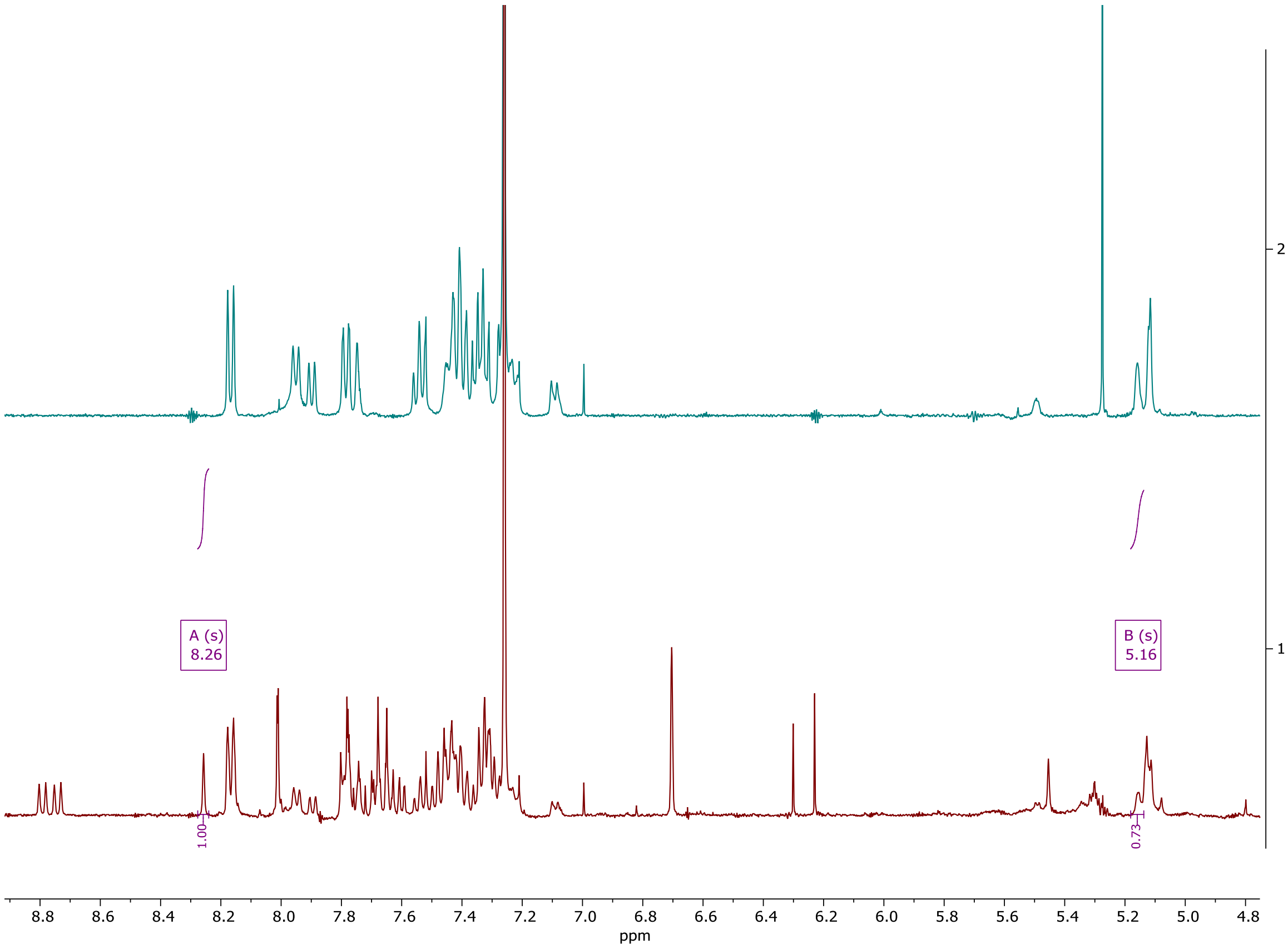

Figure S8. Stacked ^{1}H NMR spectra of PMA-functionalized OFP before sonication (top) and after 4 h of sonication (bottom) in $CDCl_3$.

5.9 Nuclear Magnetic Resonance Spectroscopy and Mass Spectrometry

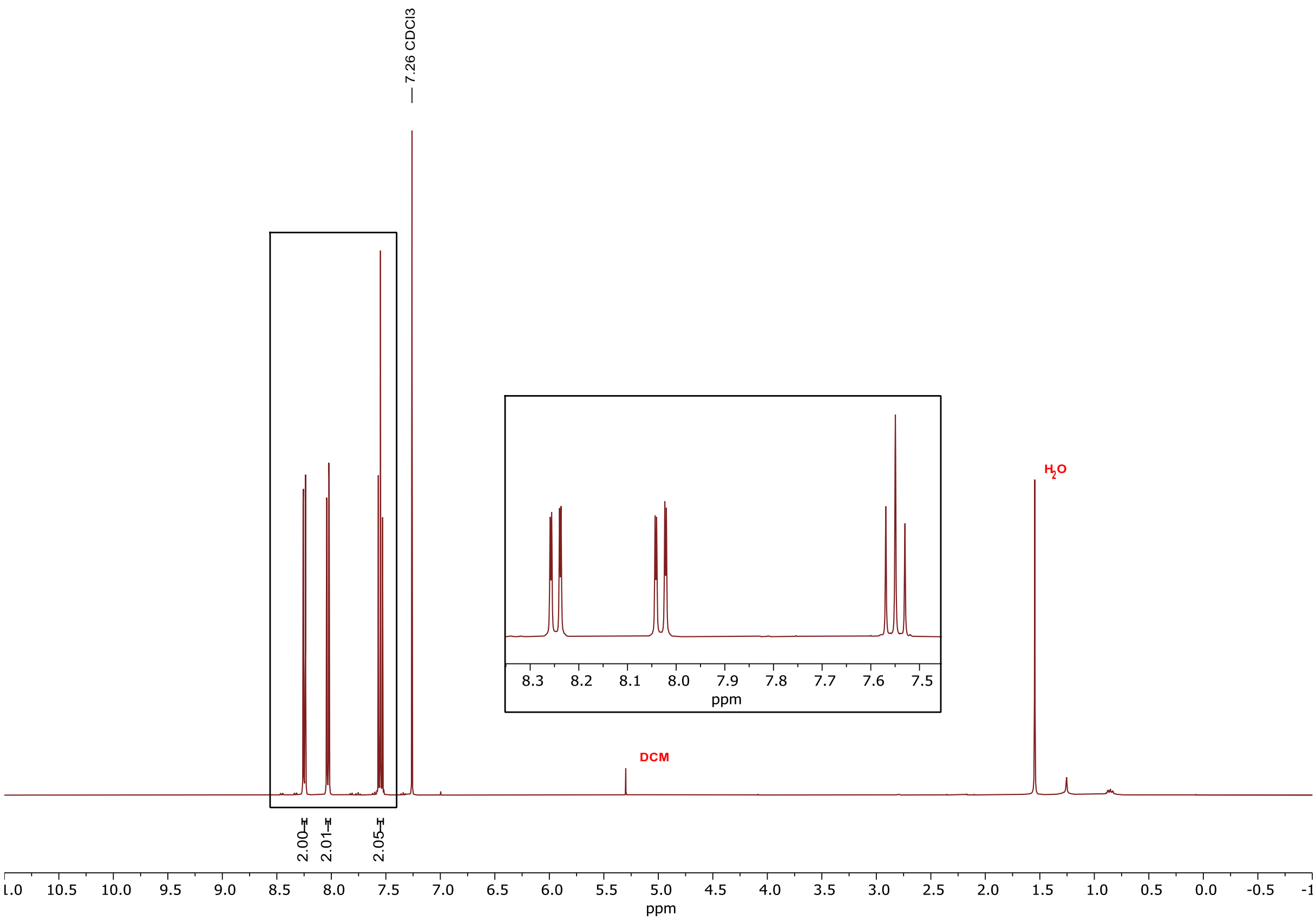

Figure S9. ^{1}H NMR spectrum of 1,8-Dibromo-9,10-anthraquinone in $CDCl_3$.

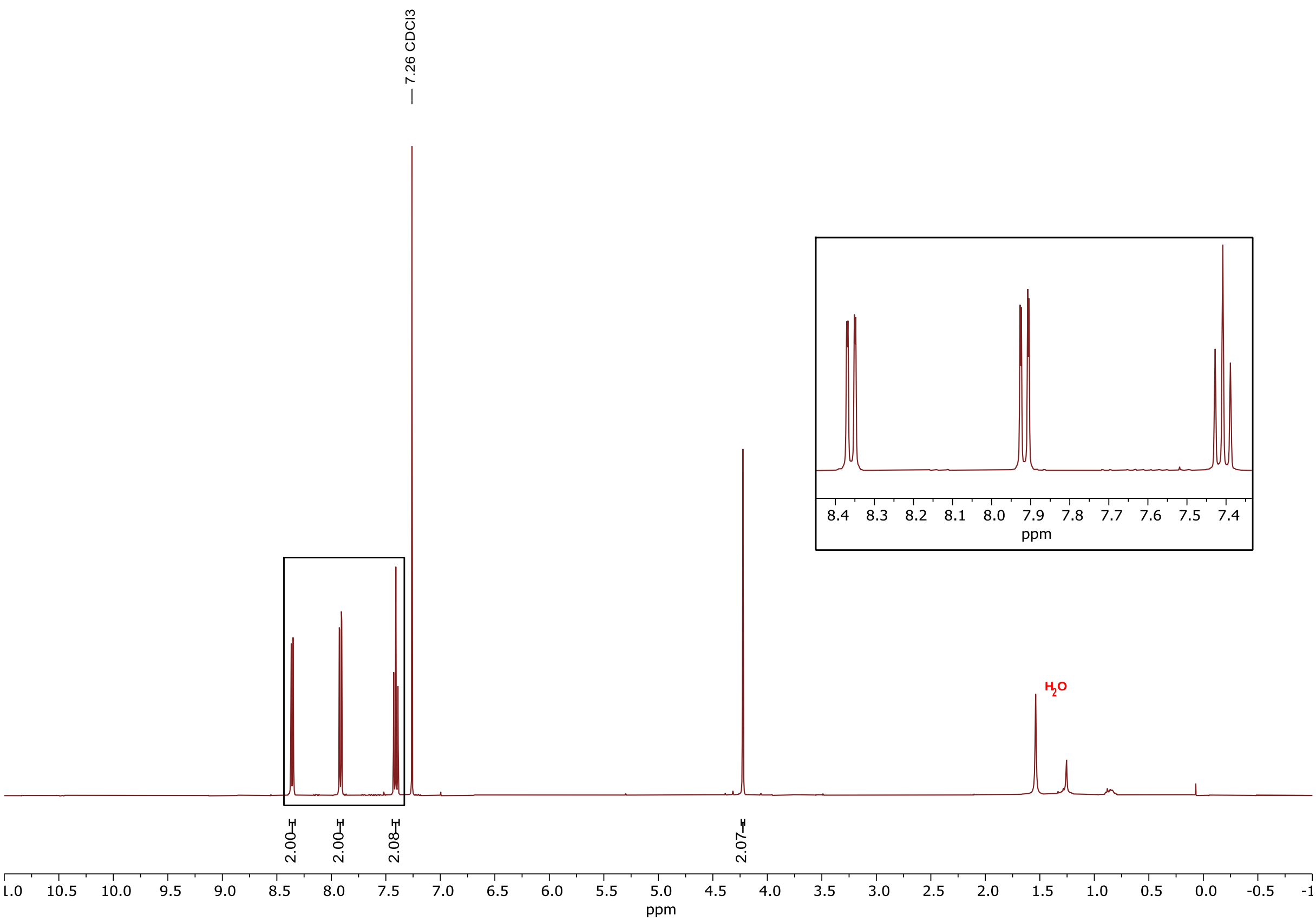

Figure S10. ^{1}H NMR spectrum of **1** in $CDCl_3$.

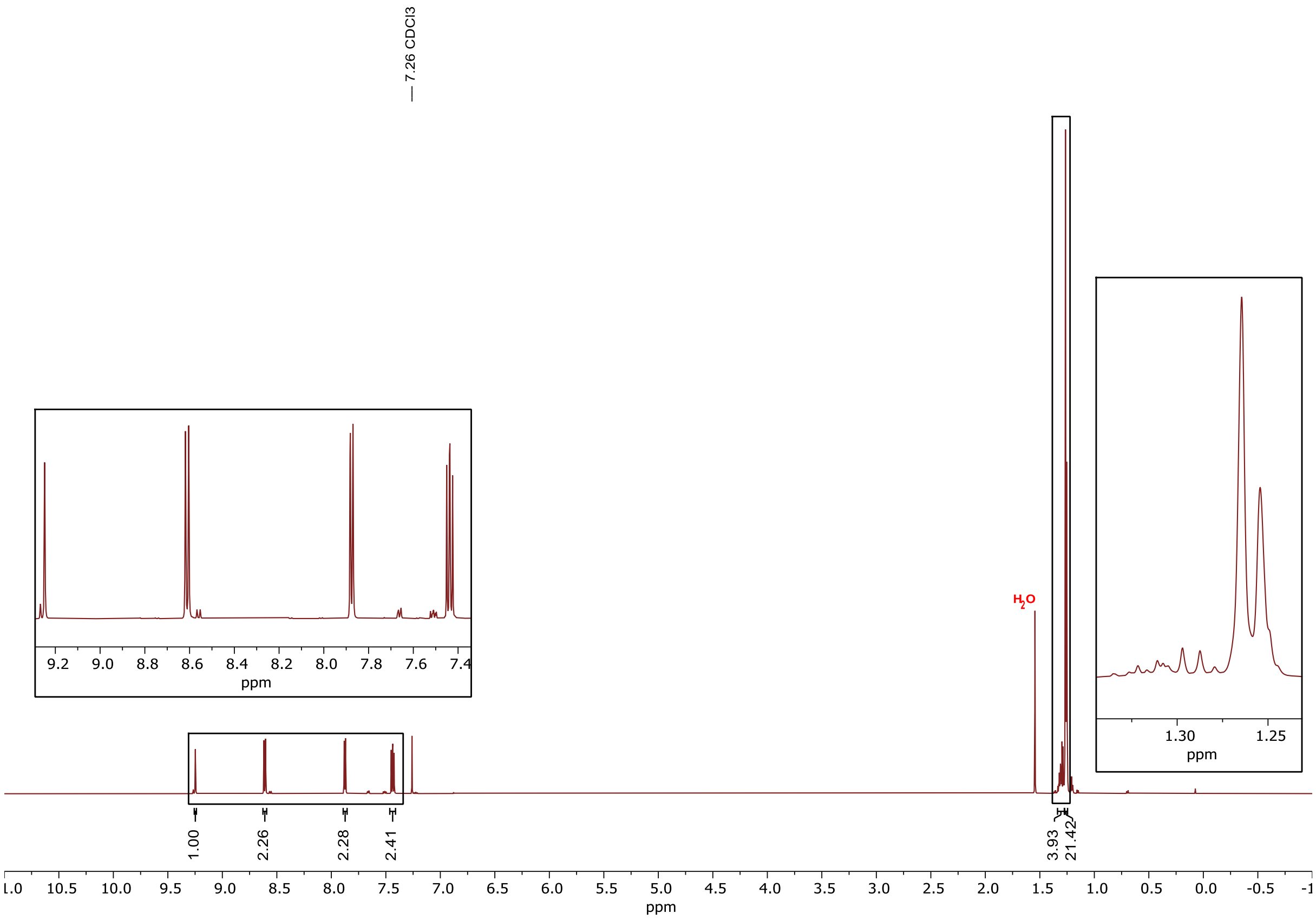

Figure S11. ^{1}H NMR spectrum of **2** in $CDCl_3$.

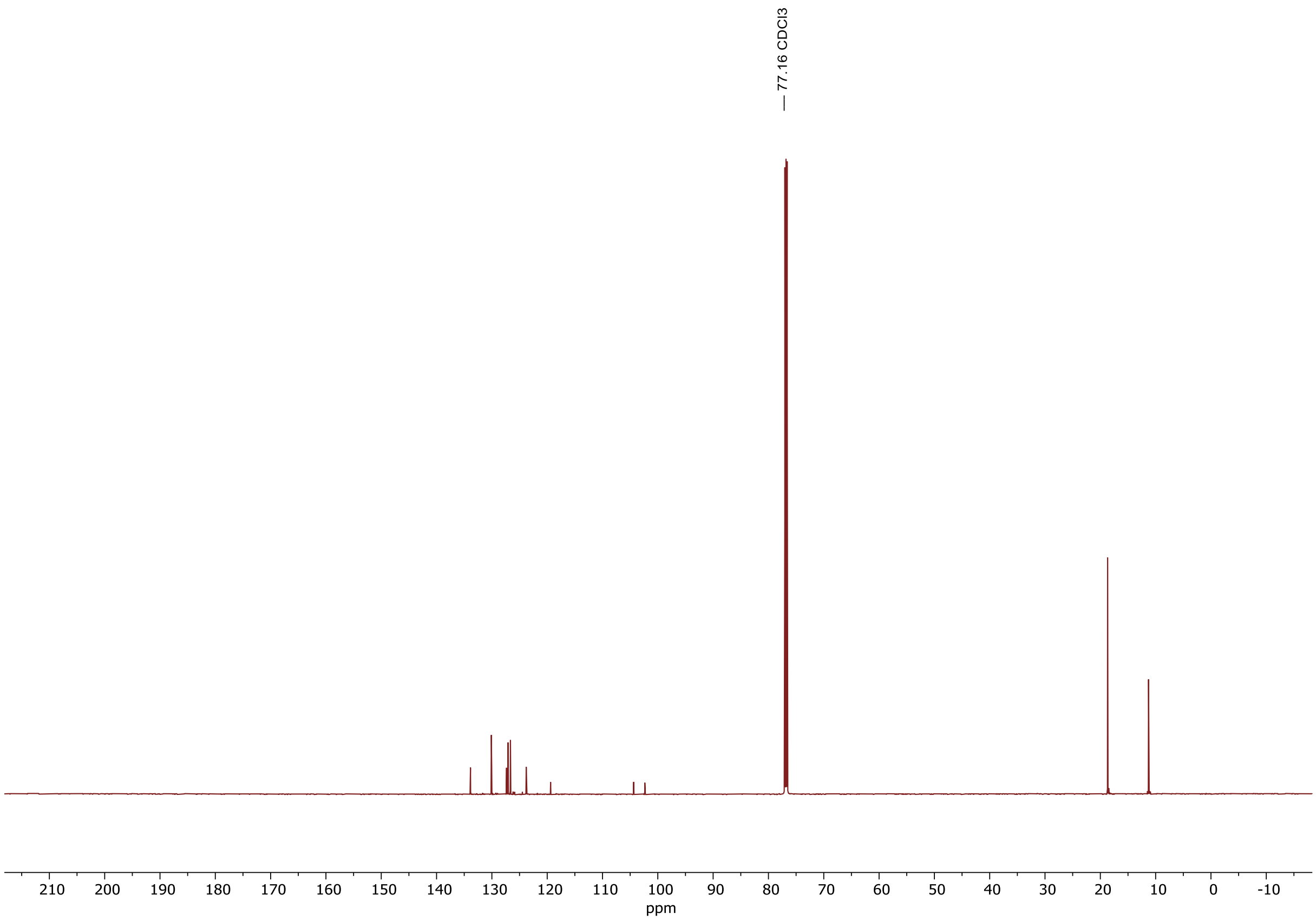

Figure S12. ^{13}C NMR spectrum of **2** in $CDCl_3$.

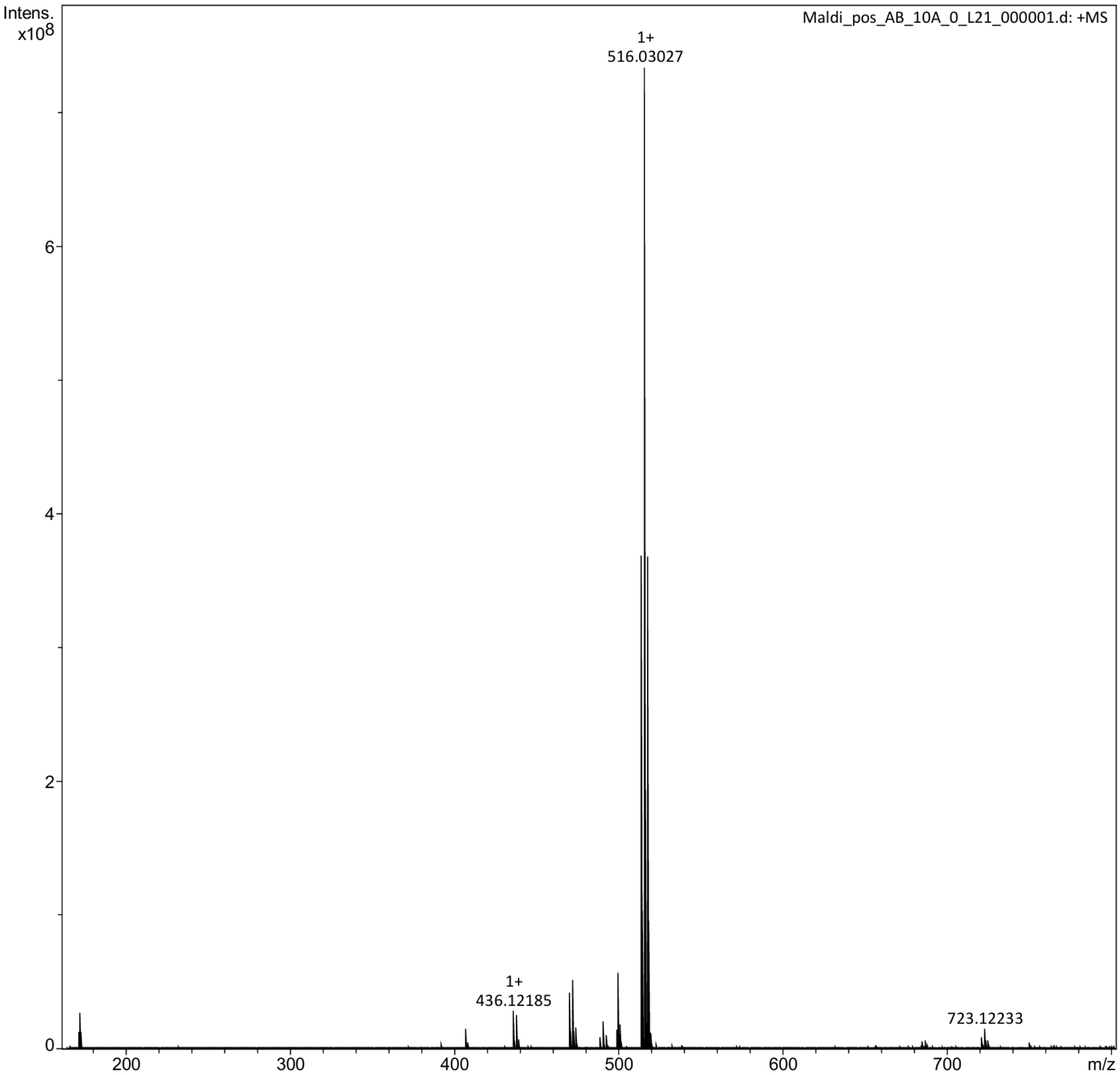

Figure S13. MALDI mass spectrum of substance **2** in DCTB.

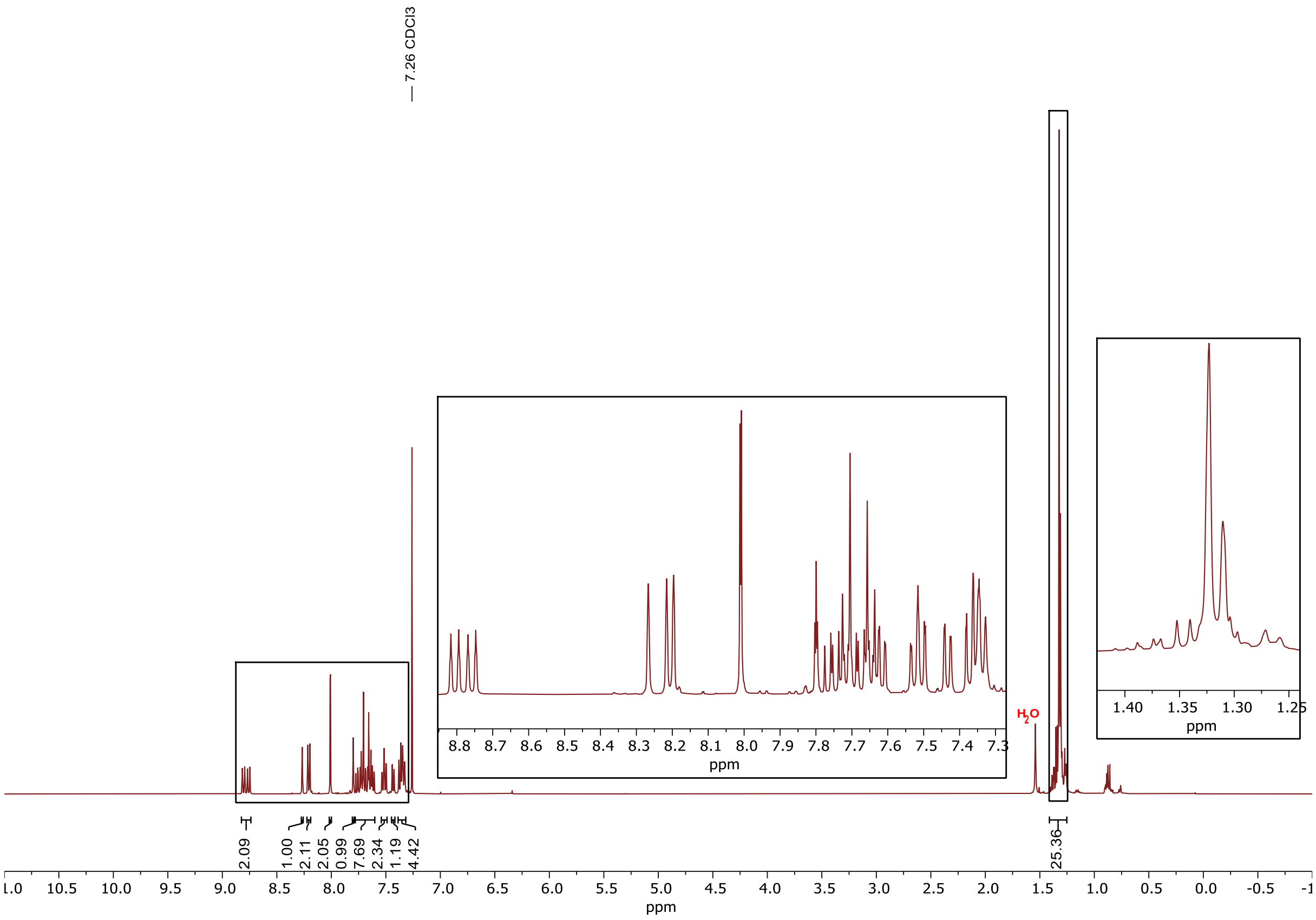

Figure S14. ^{1}H NMR spectrum of **3** in $CDCl_3$.

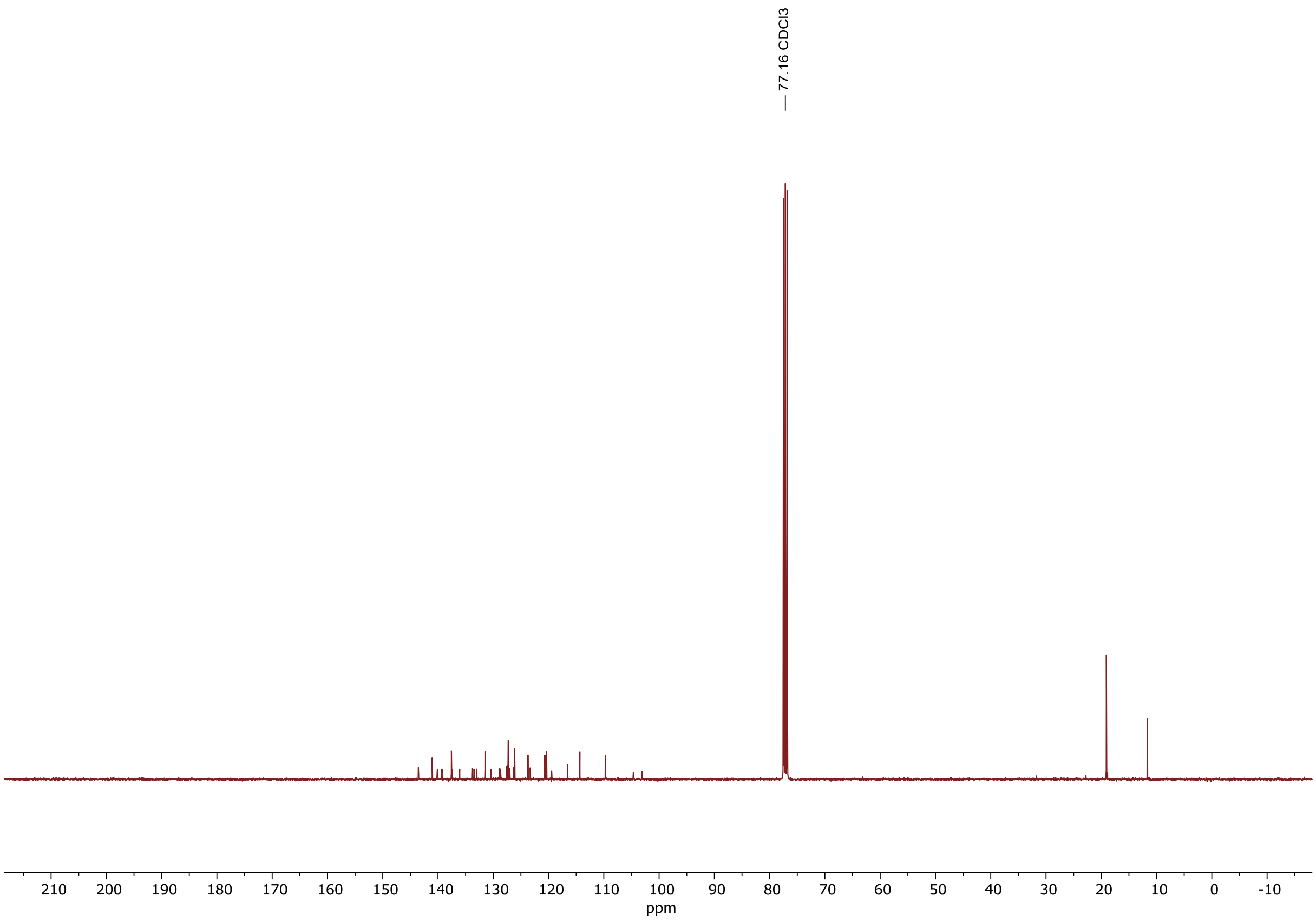

Figure S15. ^{13}C NMR spectrum of **3** in $CDCl_3$.

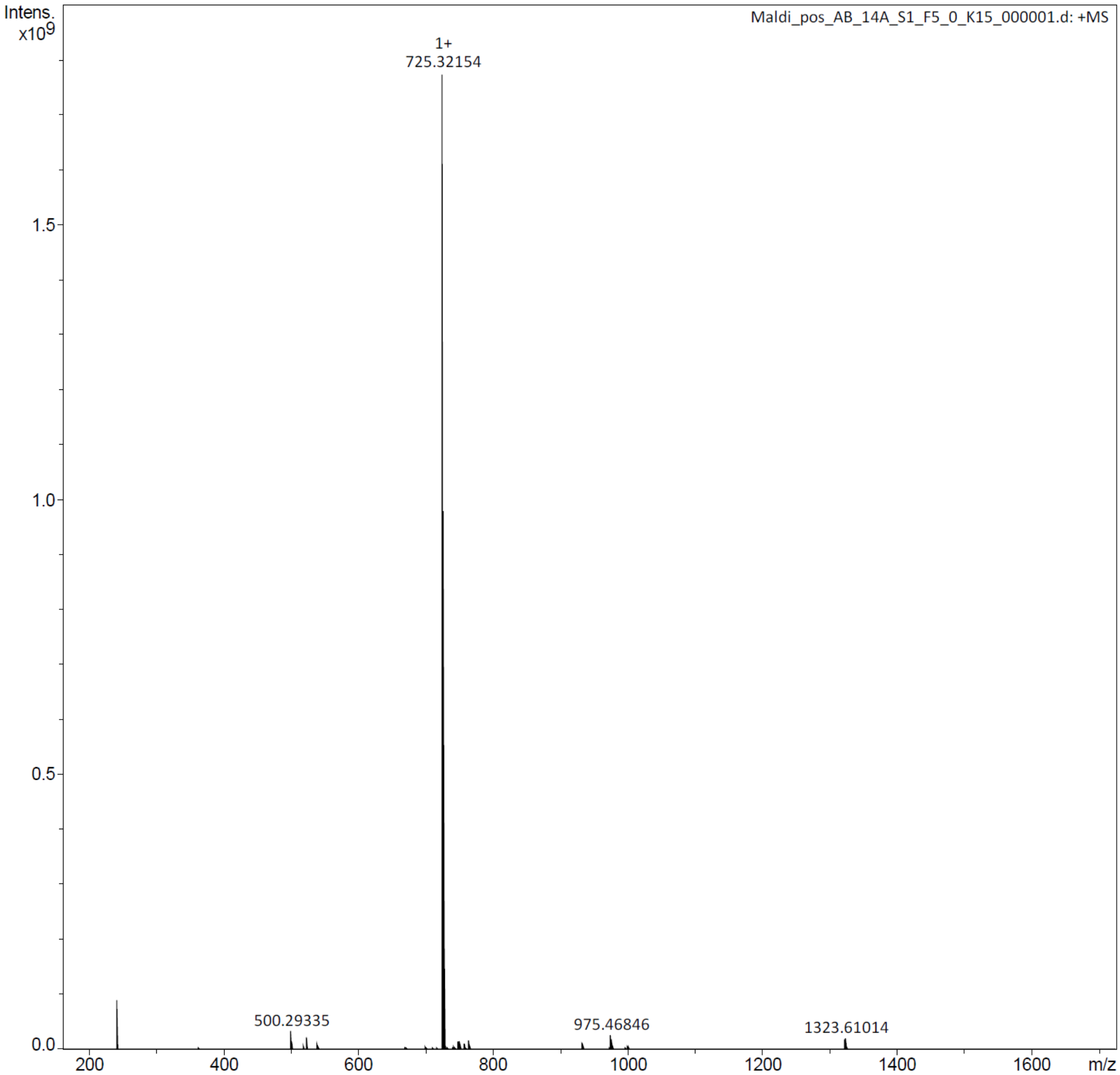

Figure S16. MALDI mass spectrum of substance **3** in DCTB.

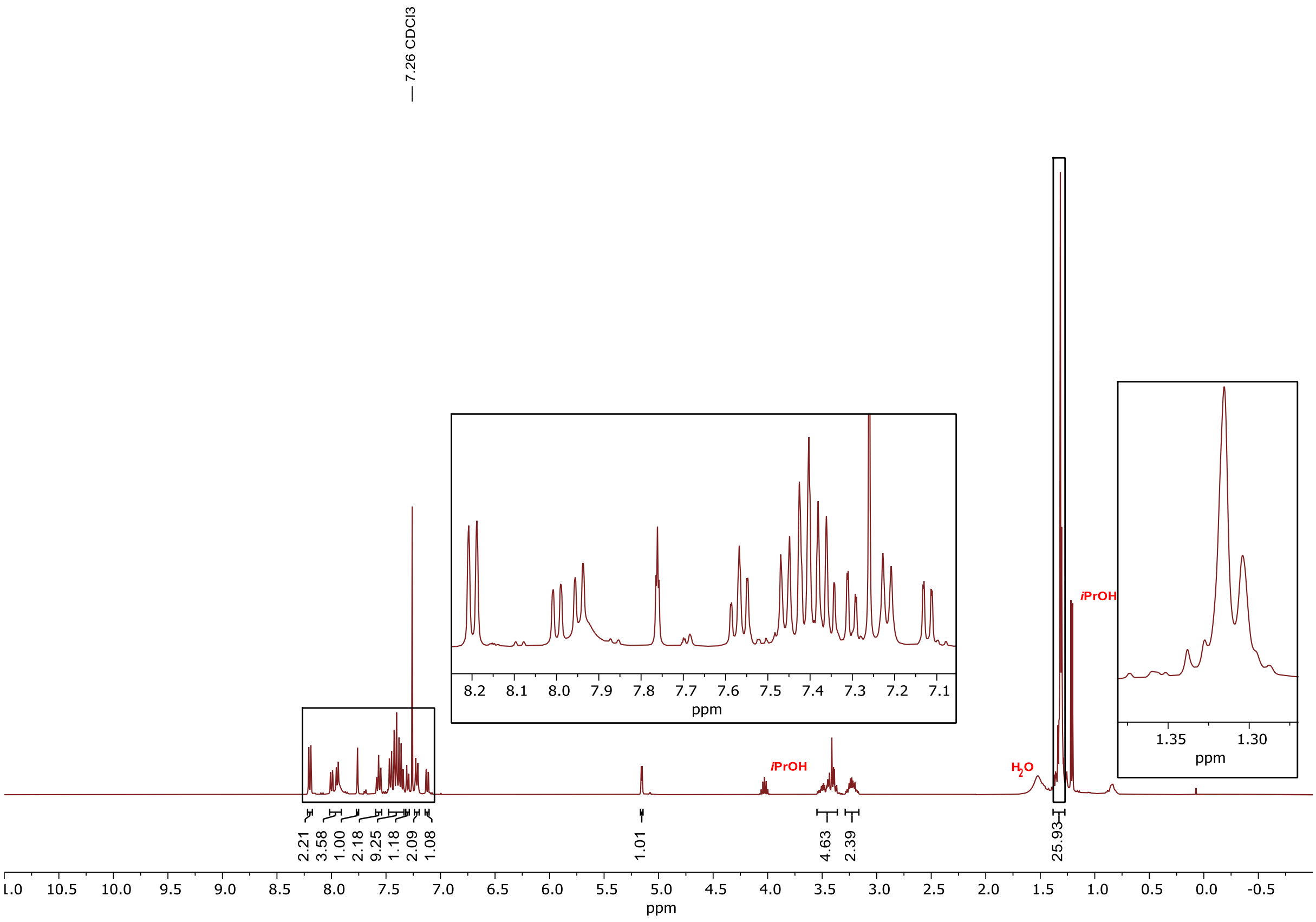

Figure S17. ^{1}H NMR spectrum of **4** in $CDCl_3$.

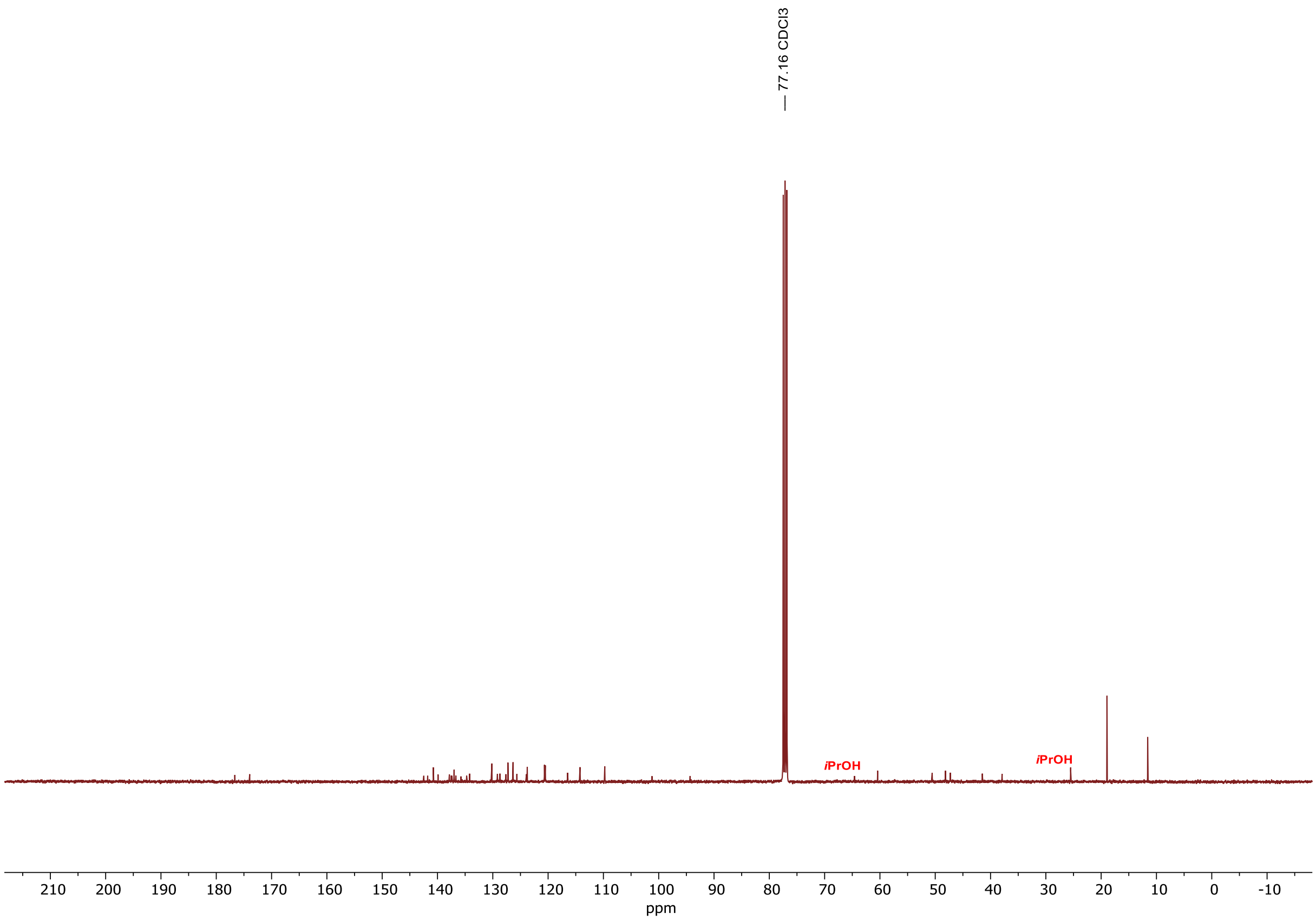

Figure S18. ^{13}C NMR spectrum of **4** in $CDCl_3$.

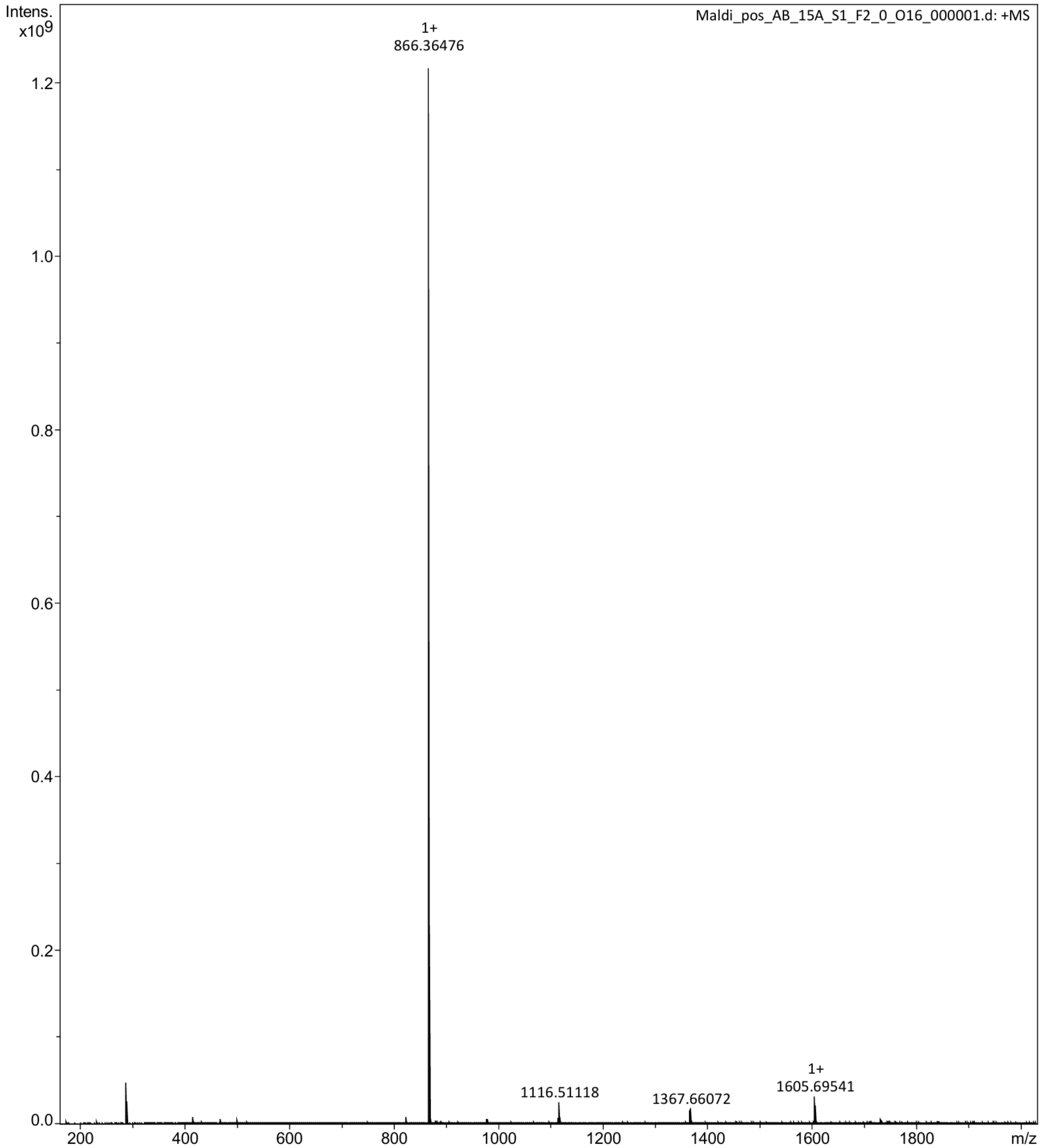

Figure S19. MALDI mass spectrum of substance **4** in DCTB.

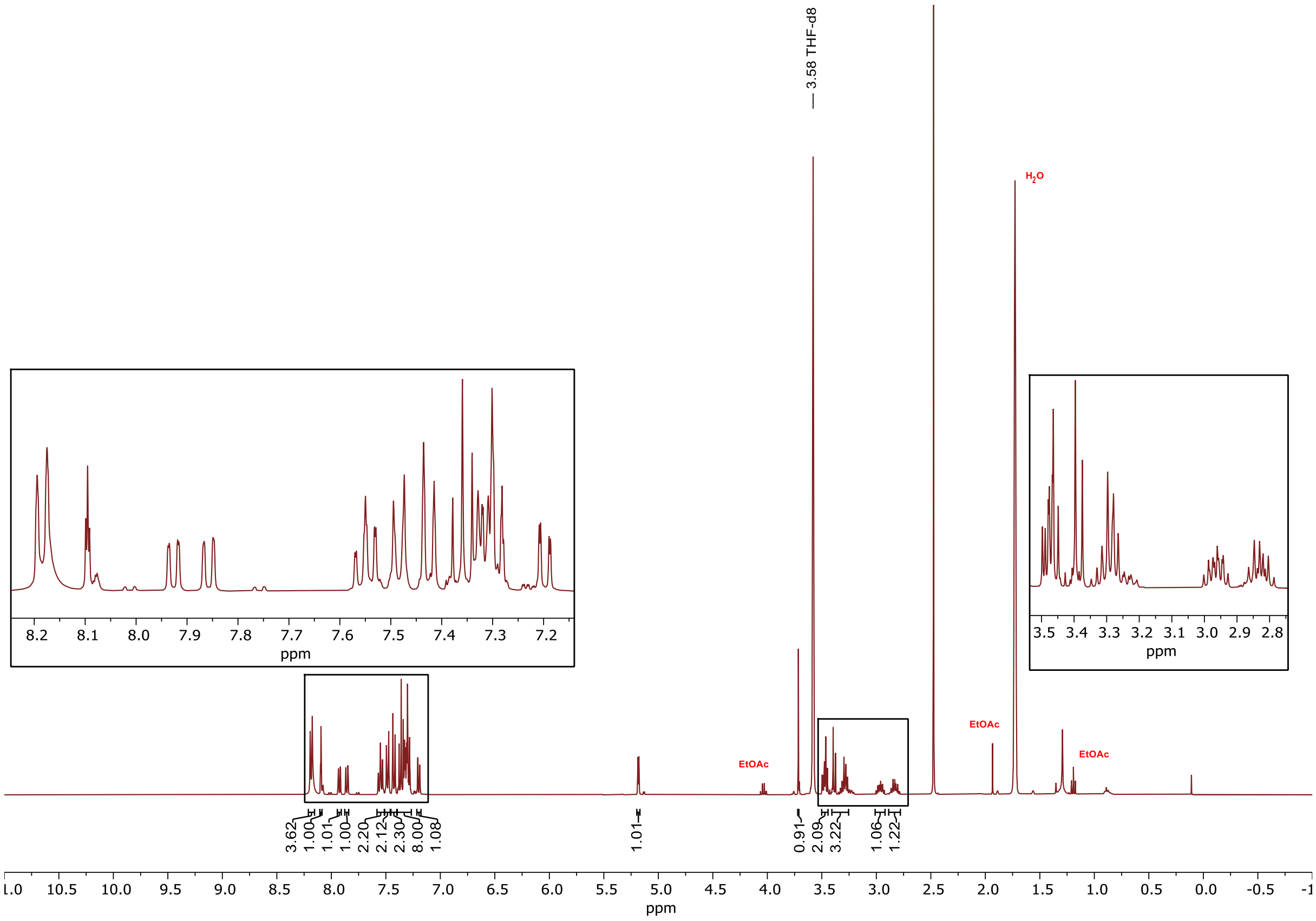

Figure S20. ^{1}H NMR spectrum of **5** in THF-d_8.

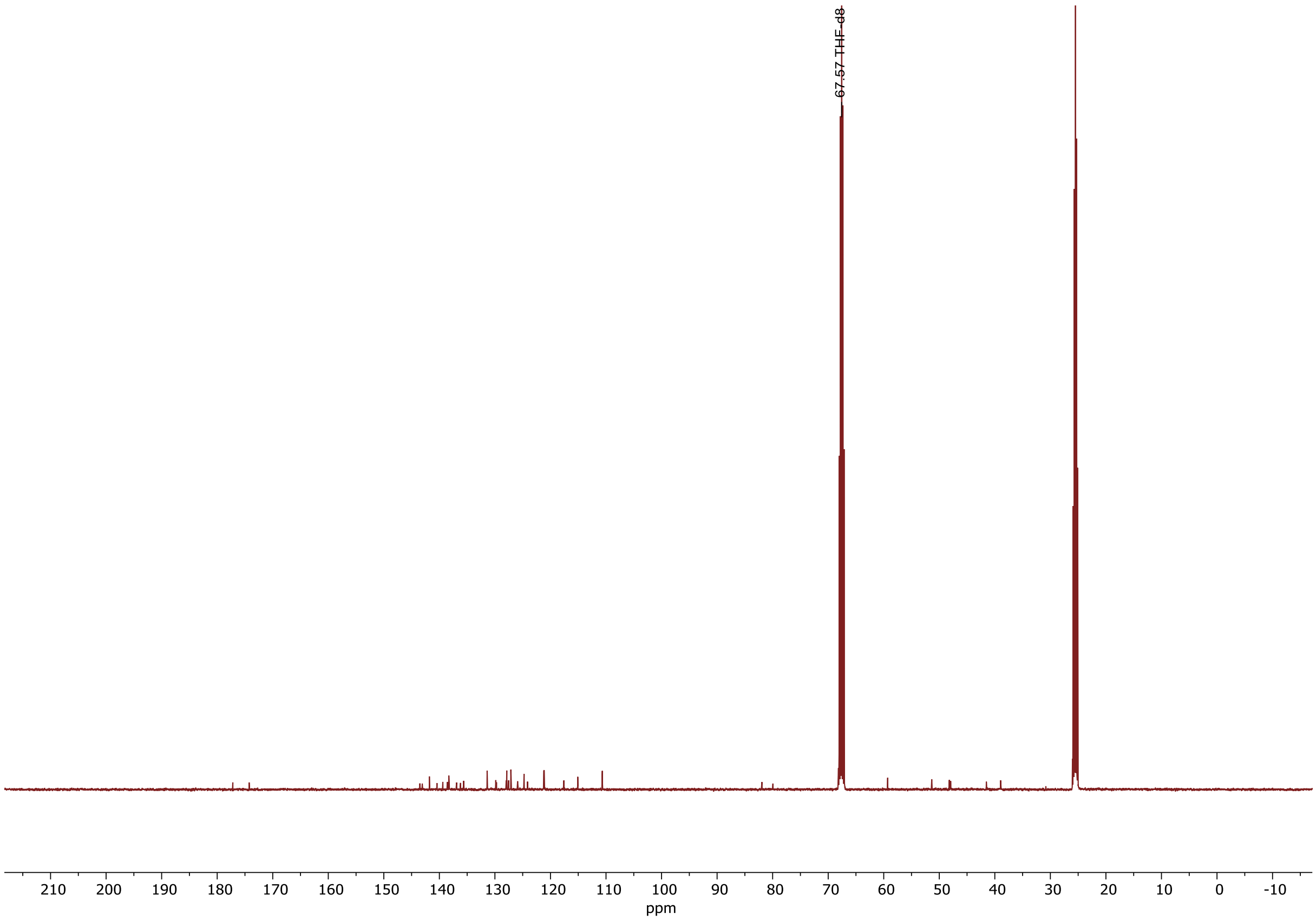

Figure S21. ^{13}C NMR spectrum of **5** in THF-d_8.

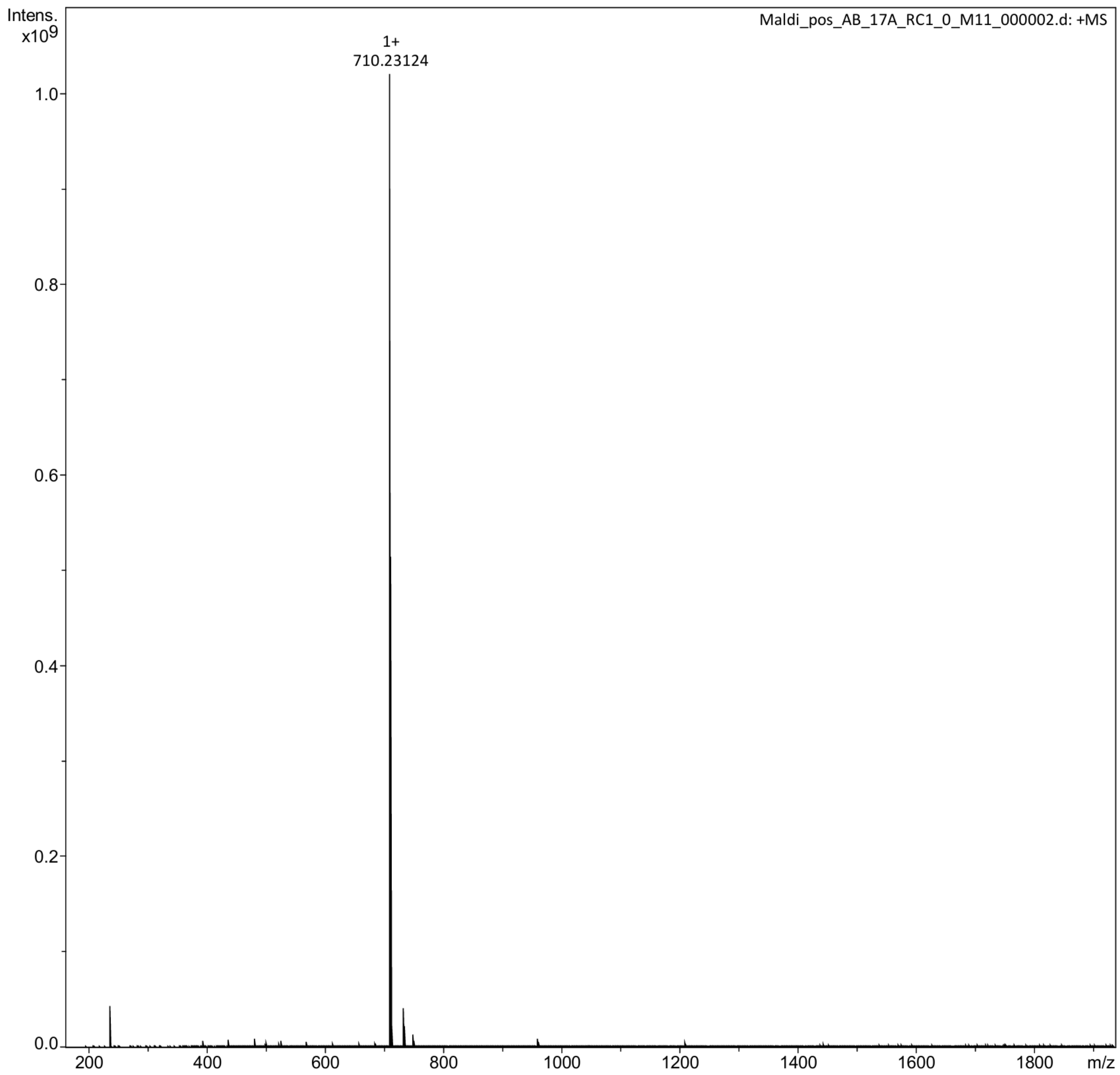

Figure S22. MALDI mass spectrum of substance **5** in DCTB.

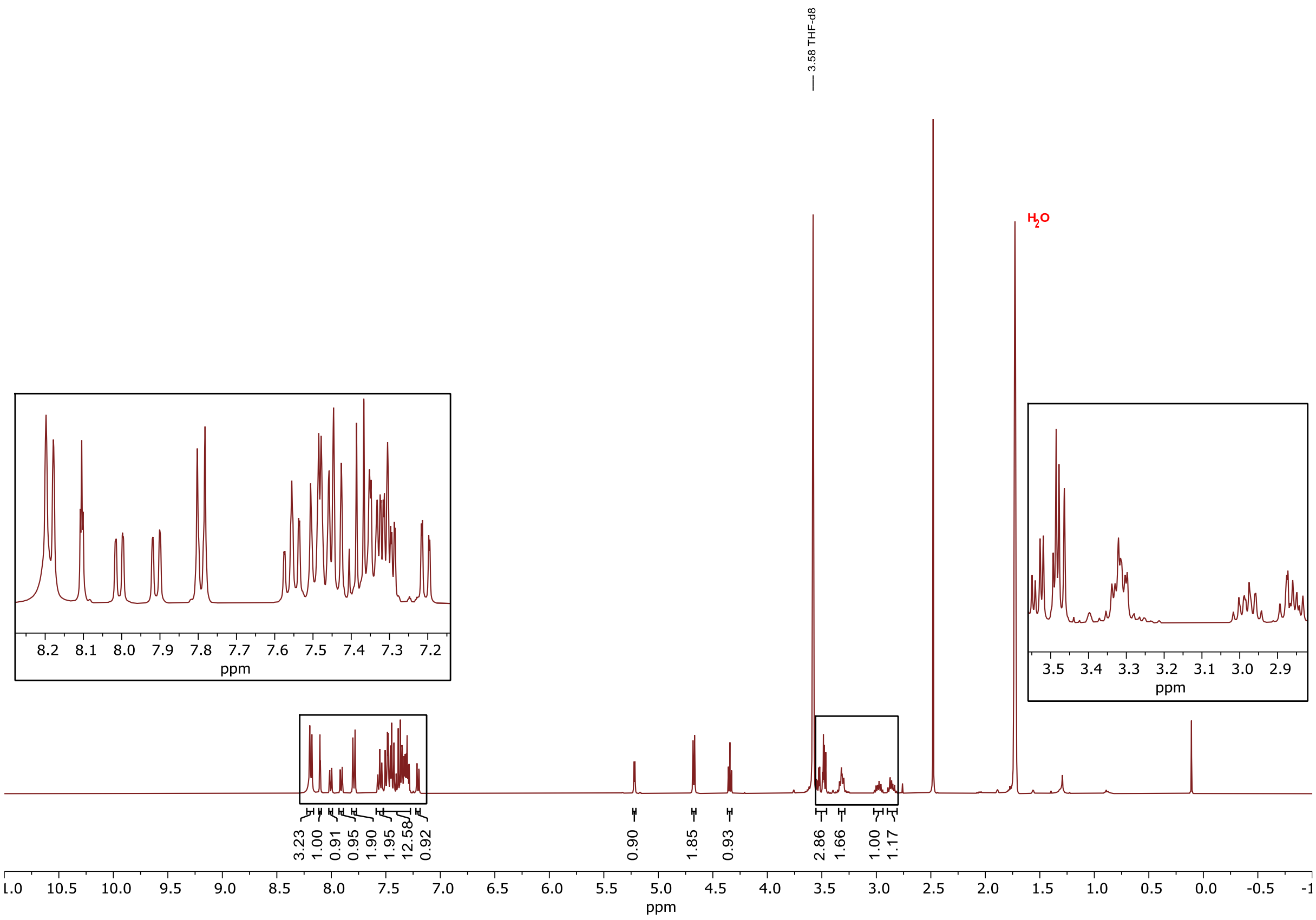

Figure S23. ^{1}H NMR spectrum of **6a** in THF-d_8.

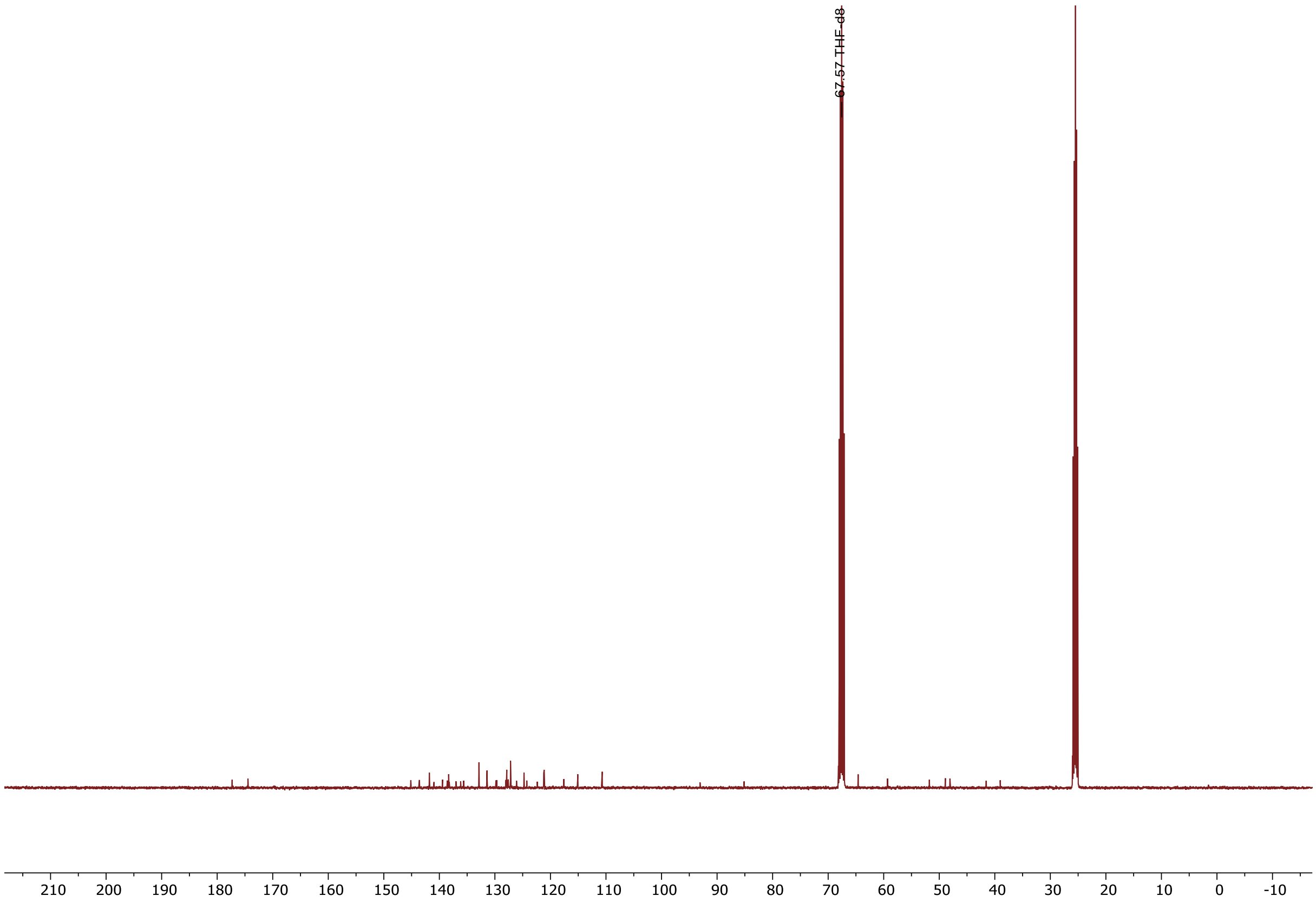

Figure S24. ^{13}C NMR spectrum of **6a** in THF-d_8.

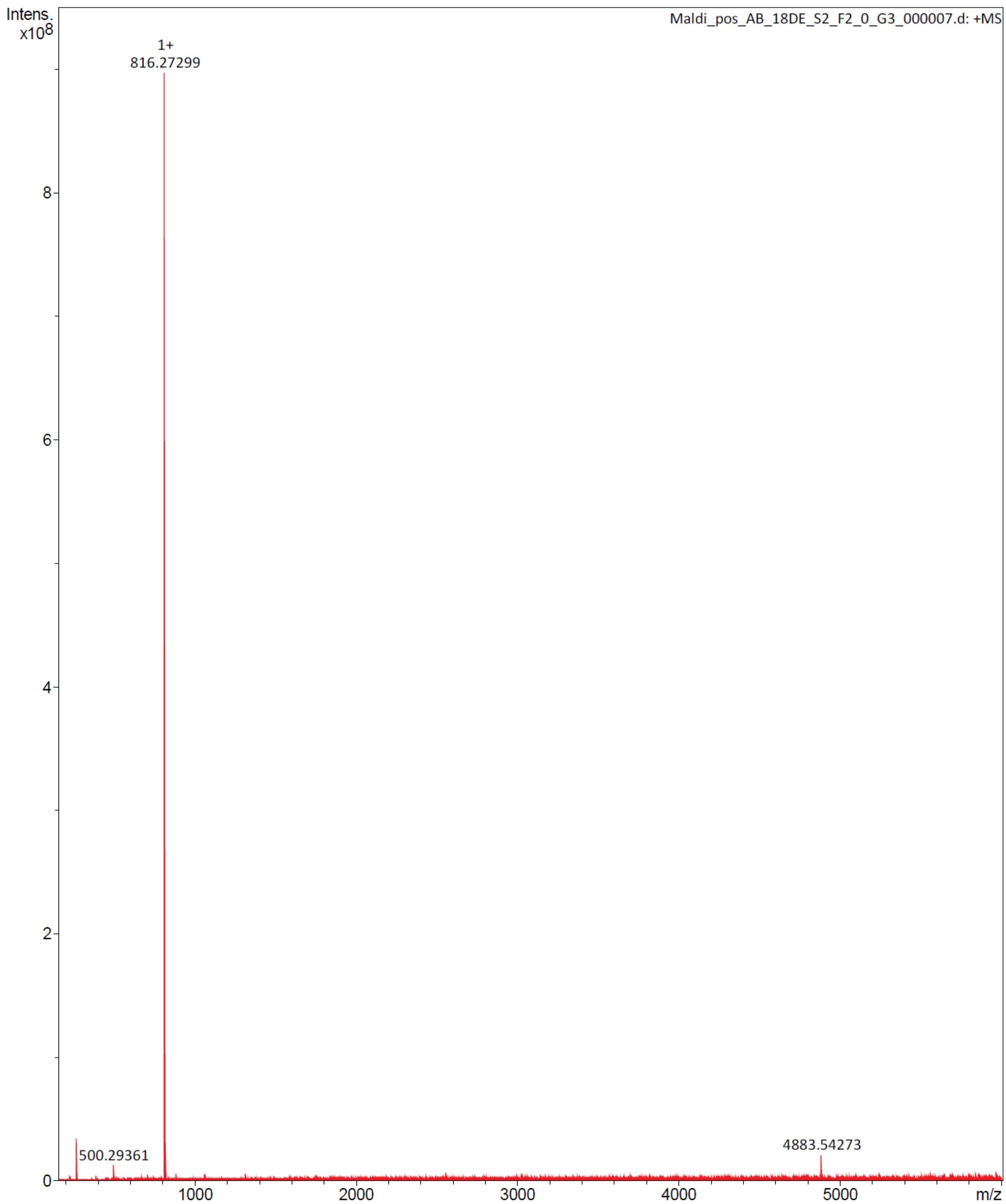

Figure S25. MALDI mass spectrum of substance **6a** in DCTB.

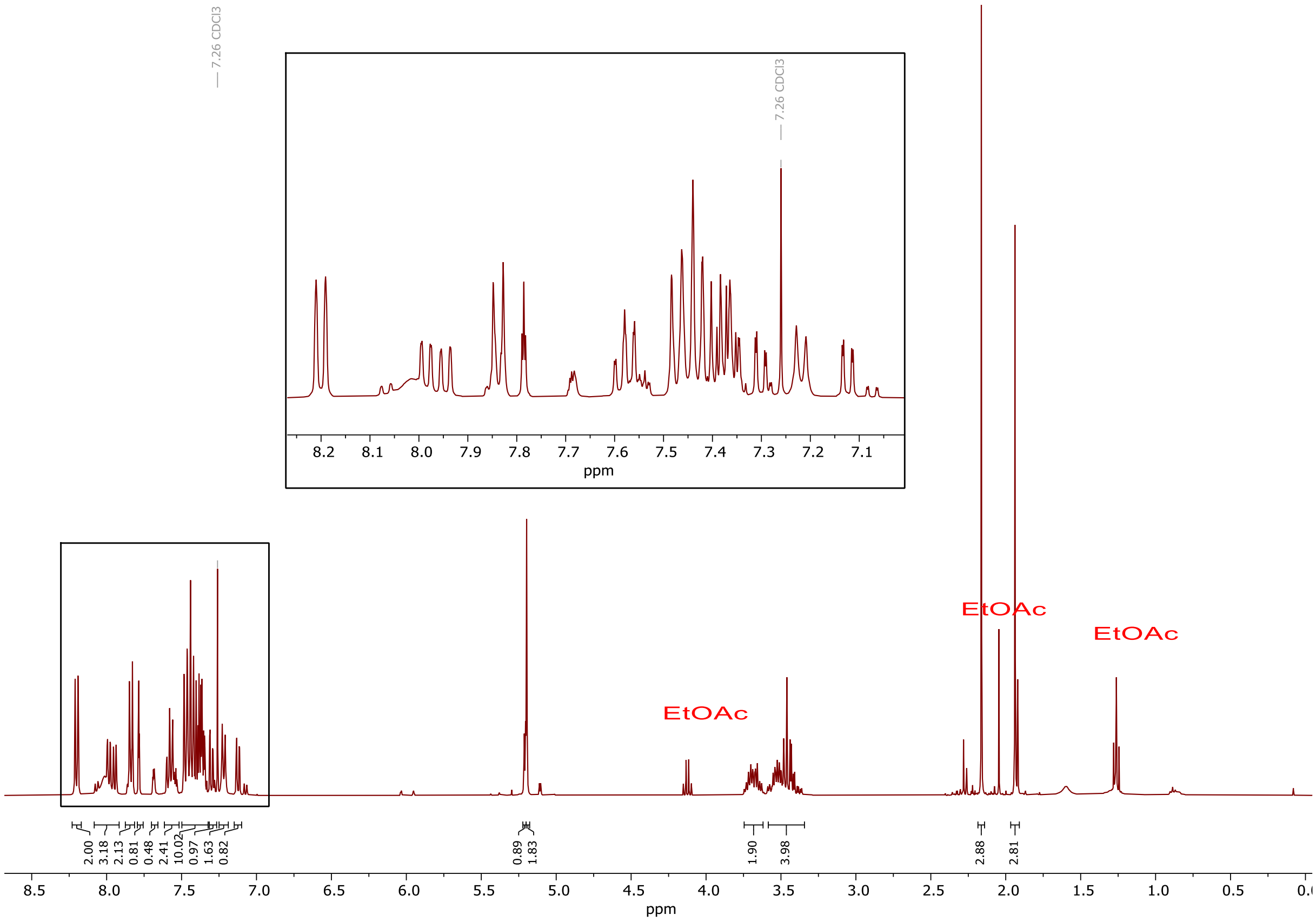

Figure S26. ^{1}H NMR spectrum of **6b** in $CDCl_3$.

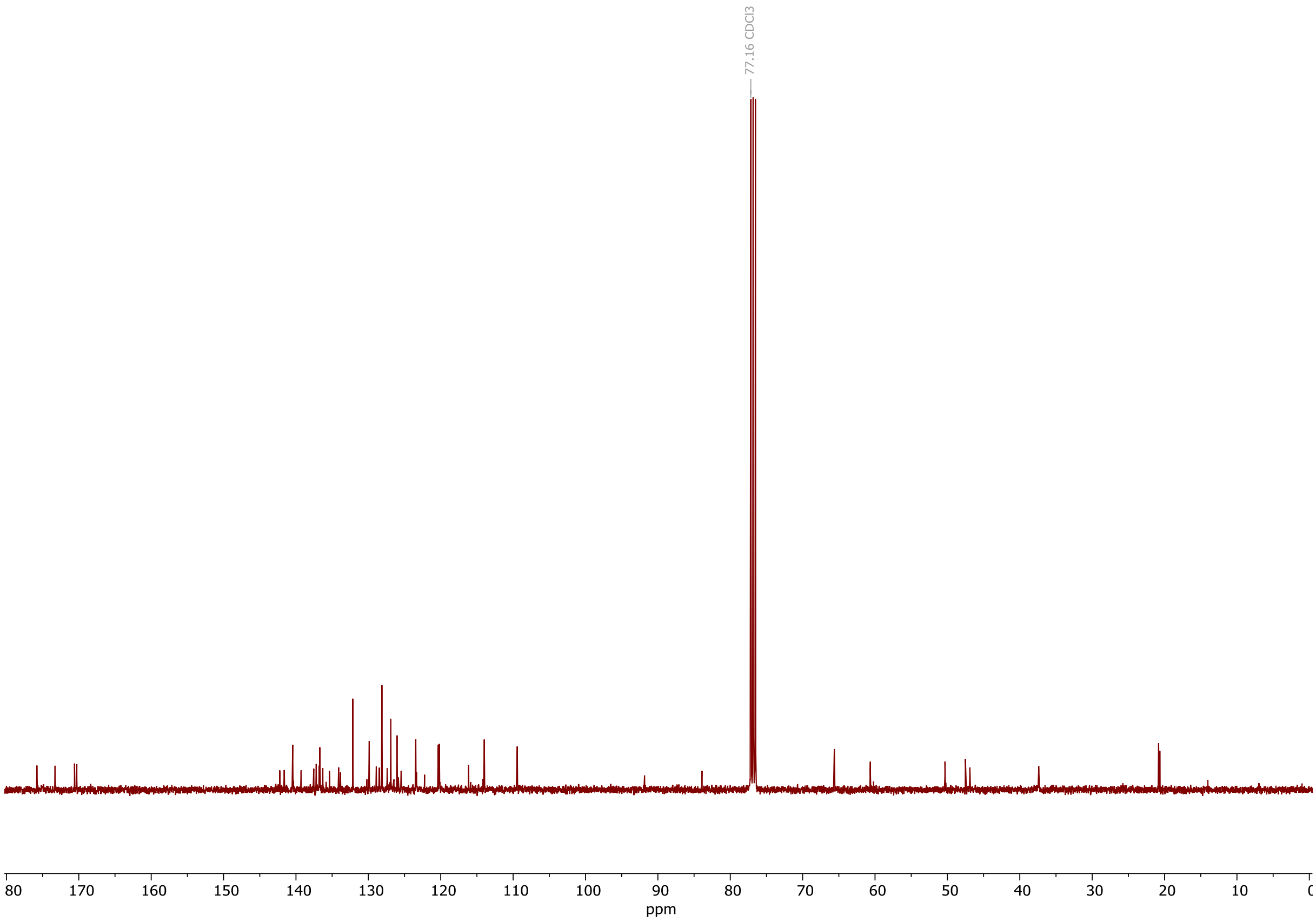

Figure S27. ^{13}C NMR spectrum of **6b** in $CDCl_3$.

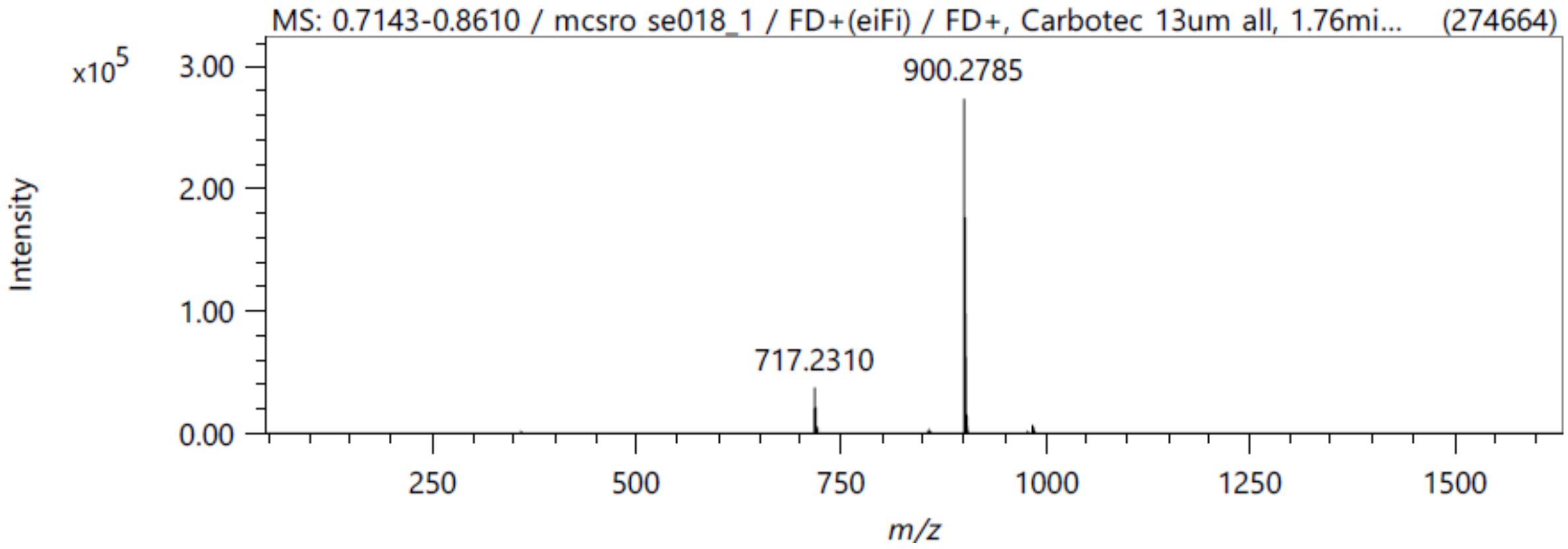

Figure S28. FD-MS spectrum of **6b**. The peak at 717.2310 originates from retro Diels-Alder reaction during the measurement.

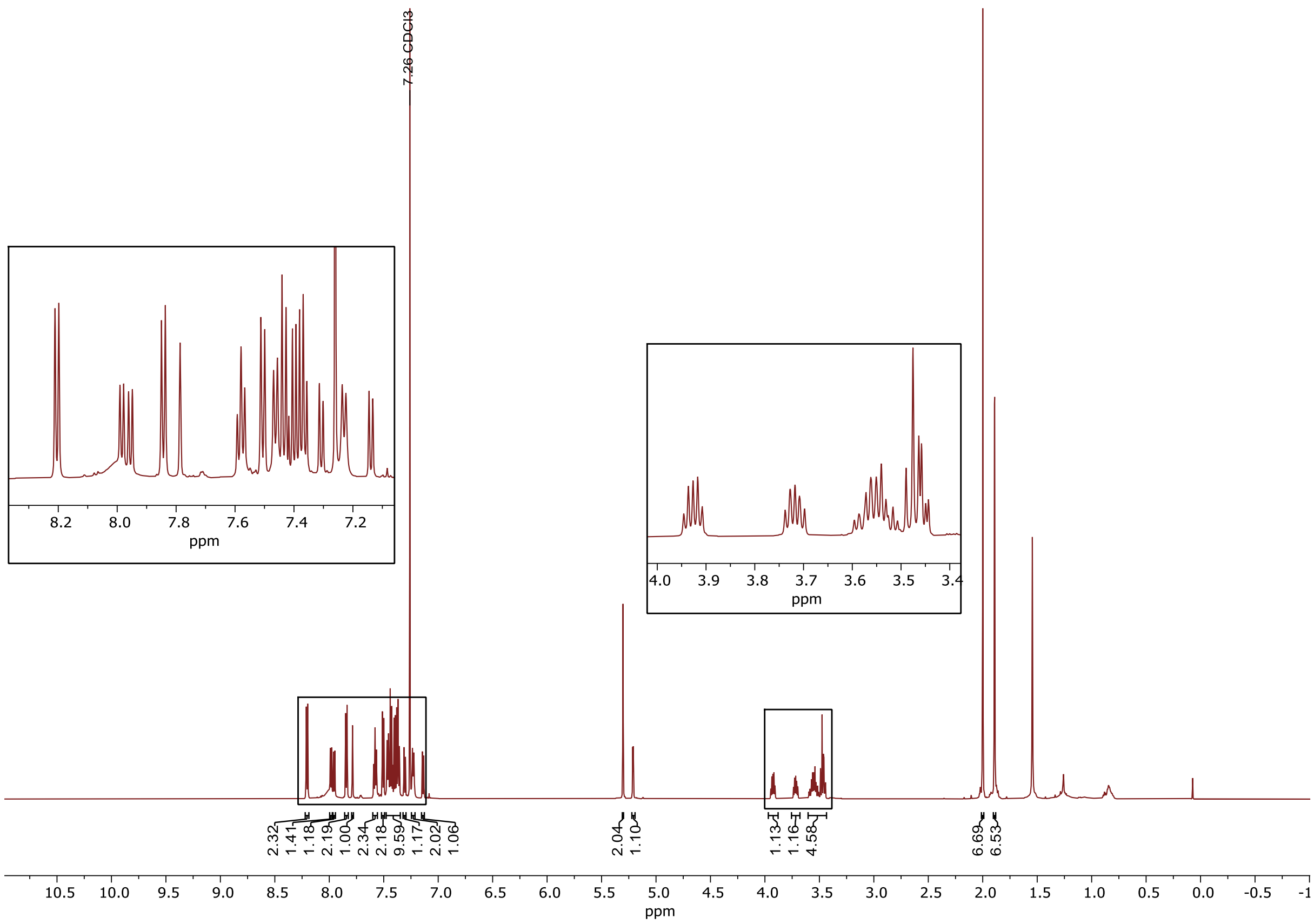

Figure S29. ^{1}H NMR spectrum of **6c** in $CDCl_3$.

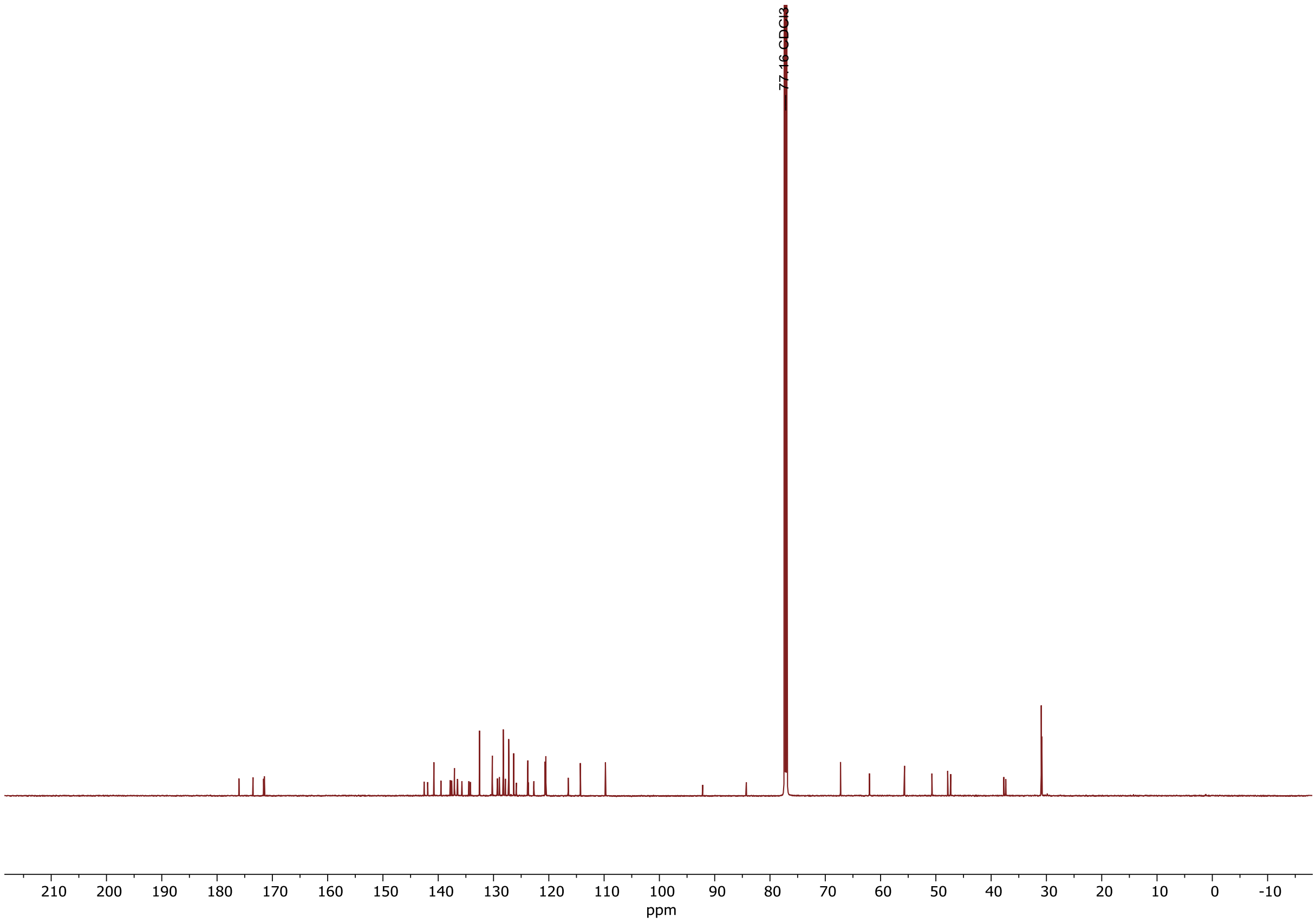

Figure S30. ^{13}C NMR spectrum of **6c** in $CDCl_3$.

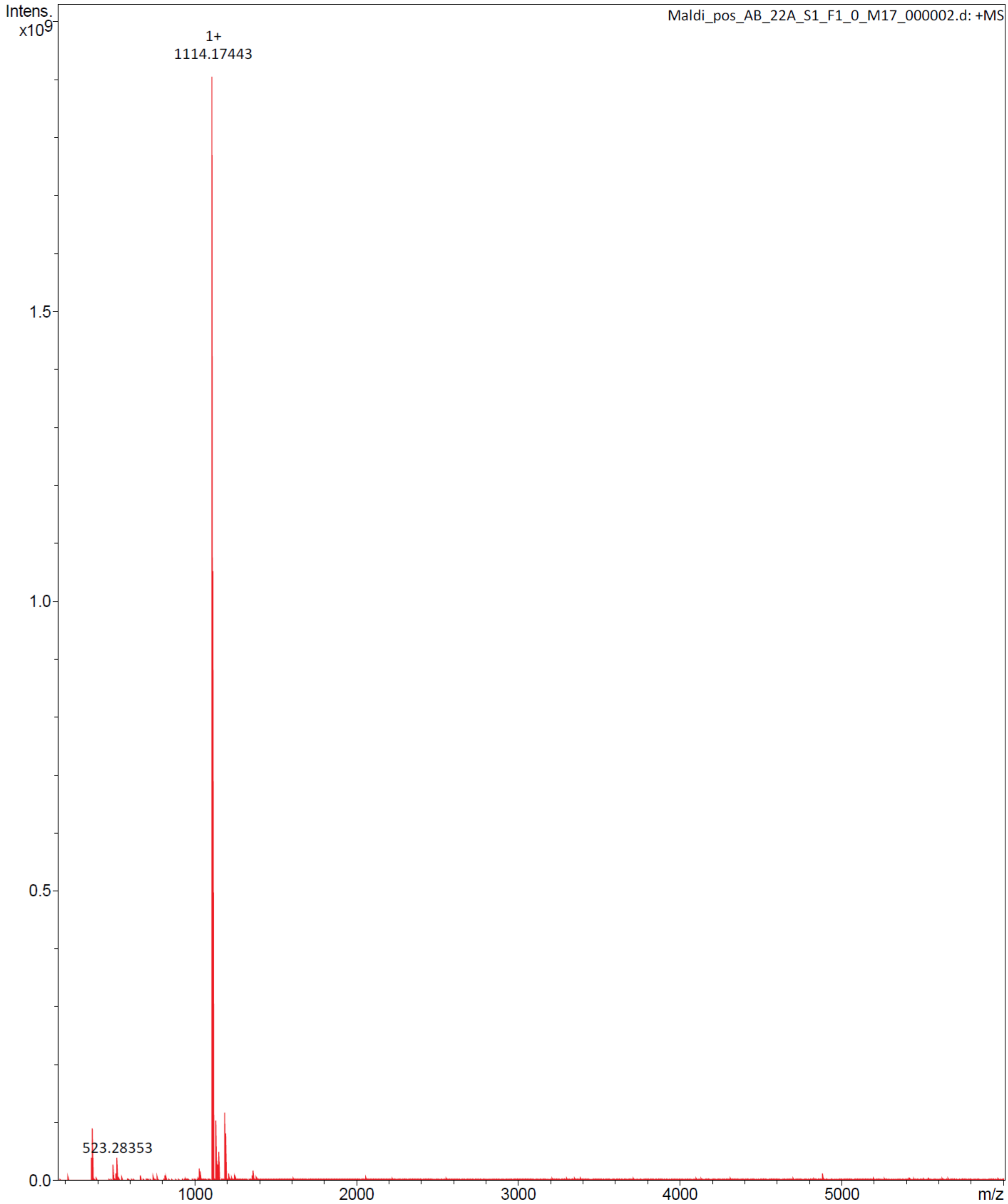

Figure S31. MALDI mass spectrum of substance **6c** in DCTB.

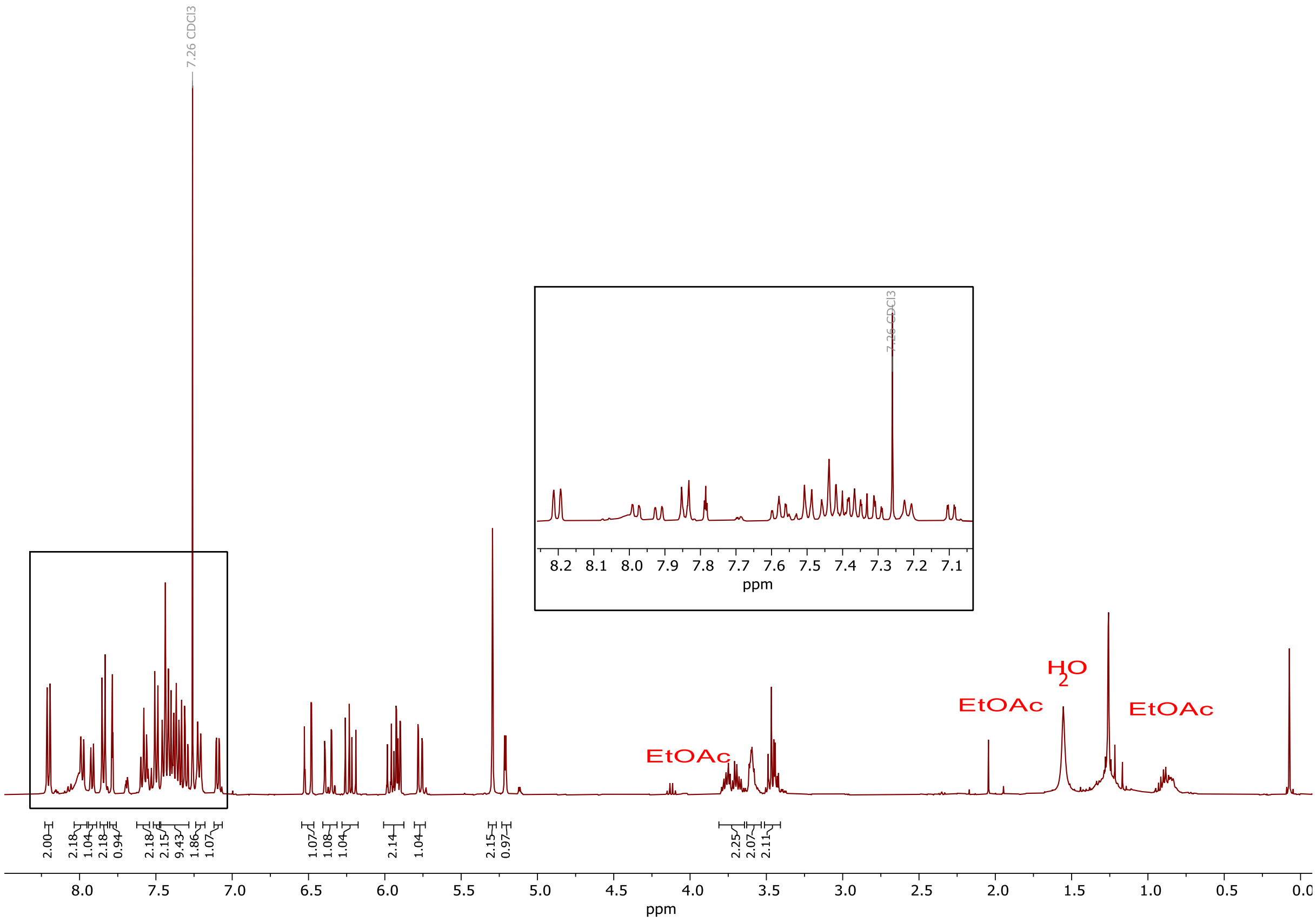

Figure S32. ^{1}H NMR spectrum of **6d** in $CDCl_3$.

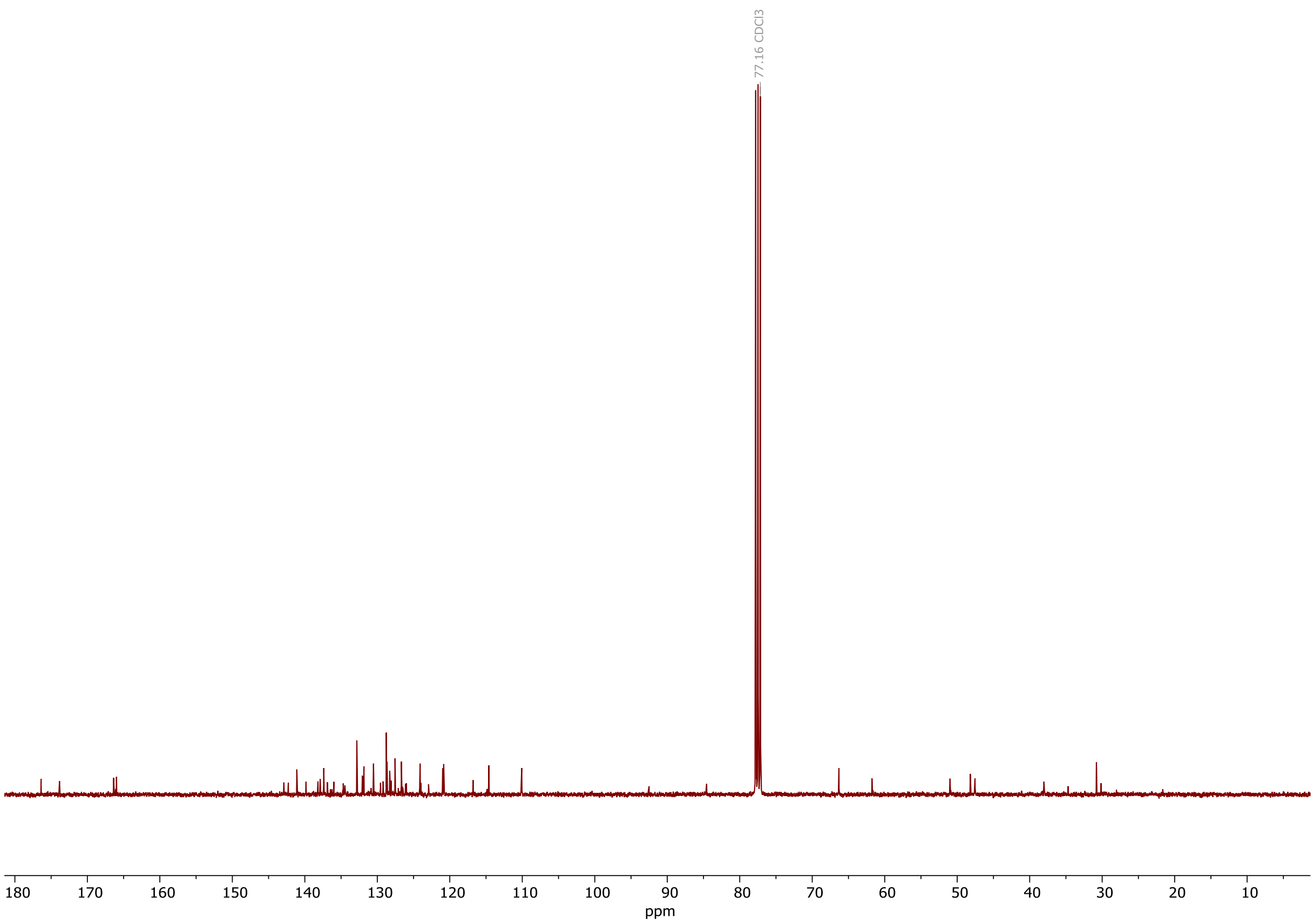

Figure S33. ^{13}C NMR spectrum of **6d** in $CDCl_3$.

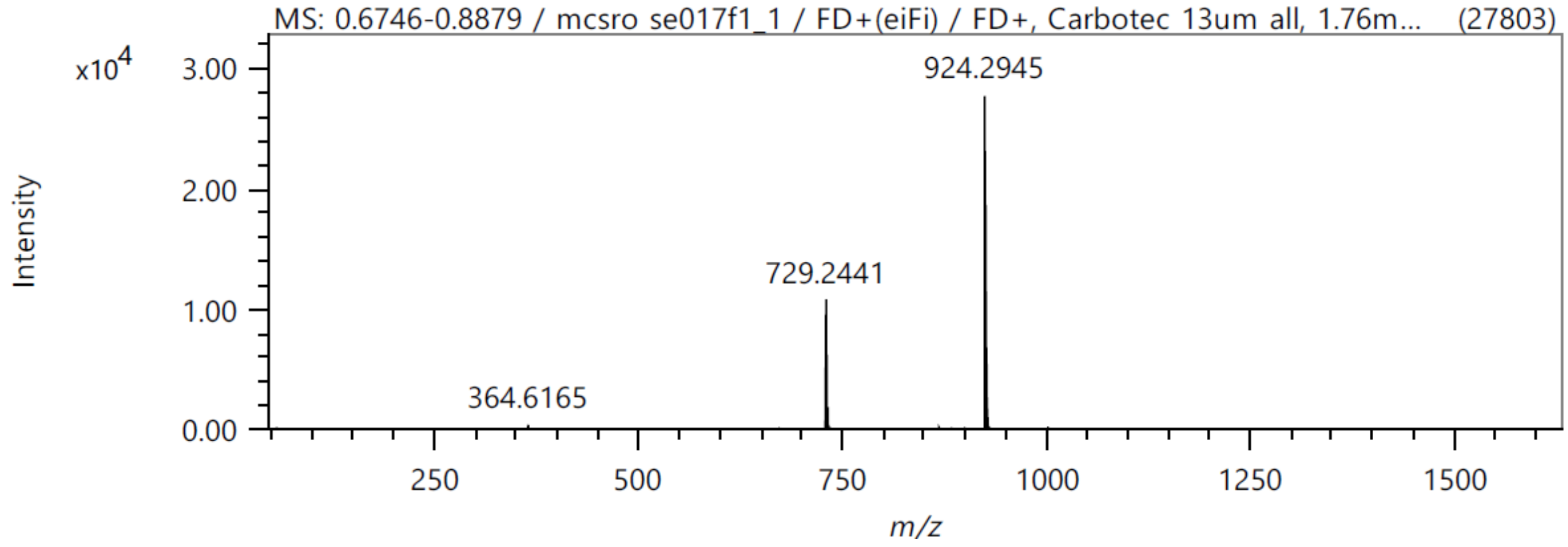

Figure S34. FD-MS spectrum of **6d**. The peak at 729.2441 originates from retro Diels-Alder reaction during the measurement.

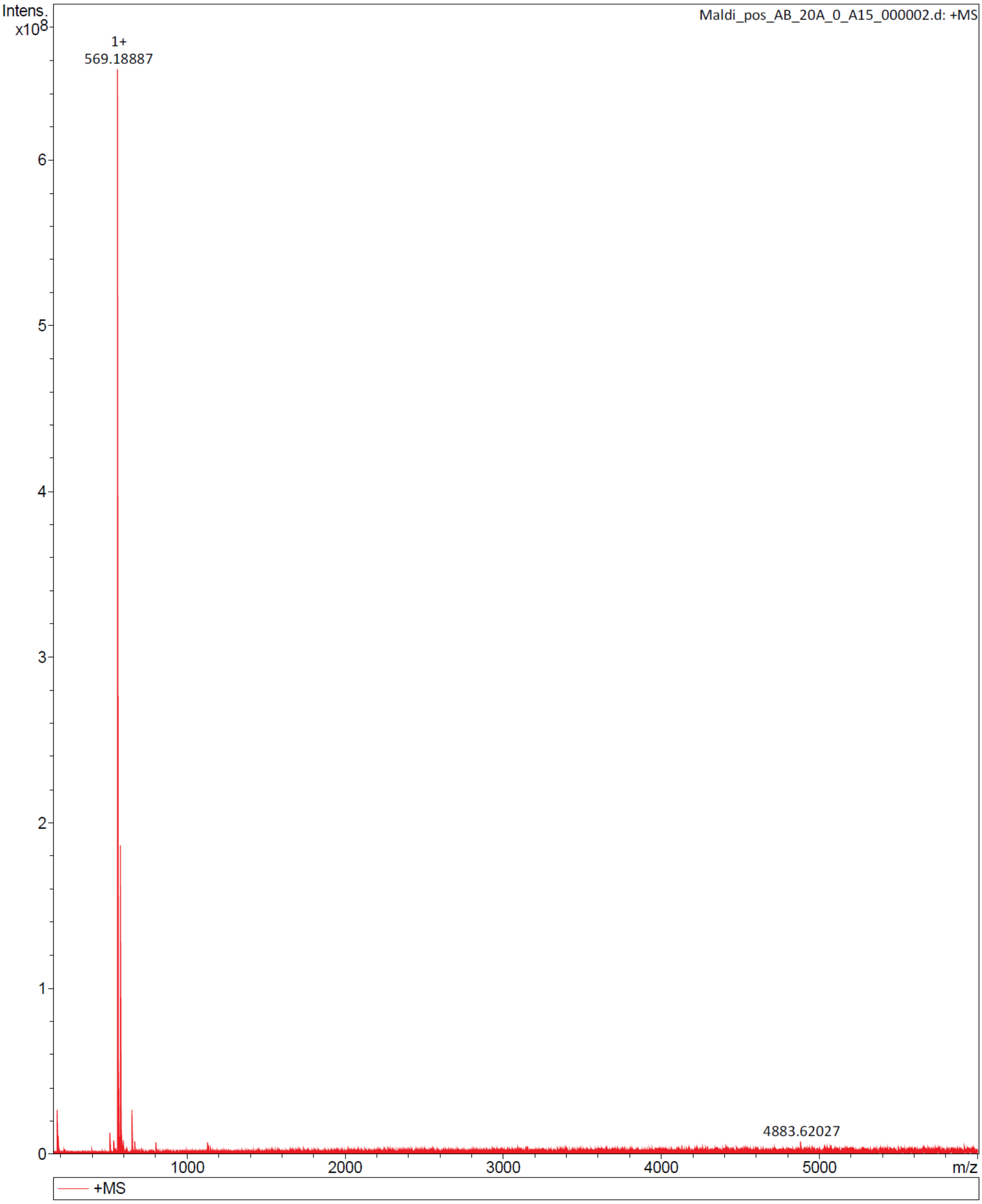

Figure S35. MALDI mass spectrum of substance **7**.

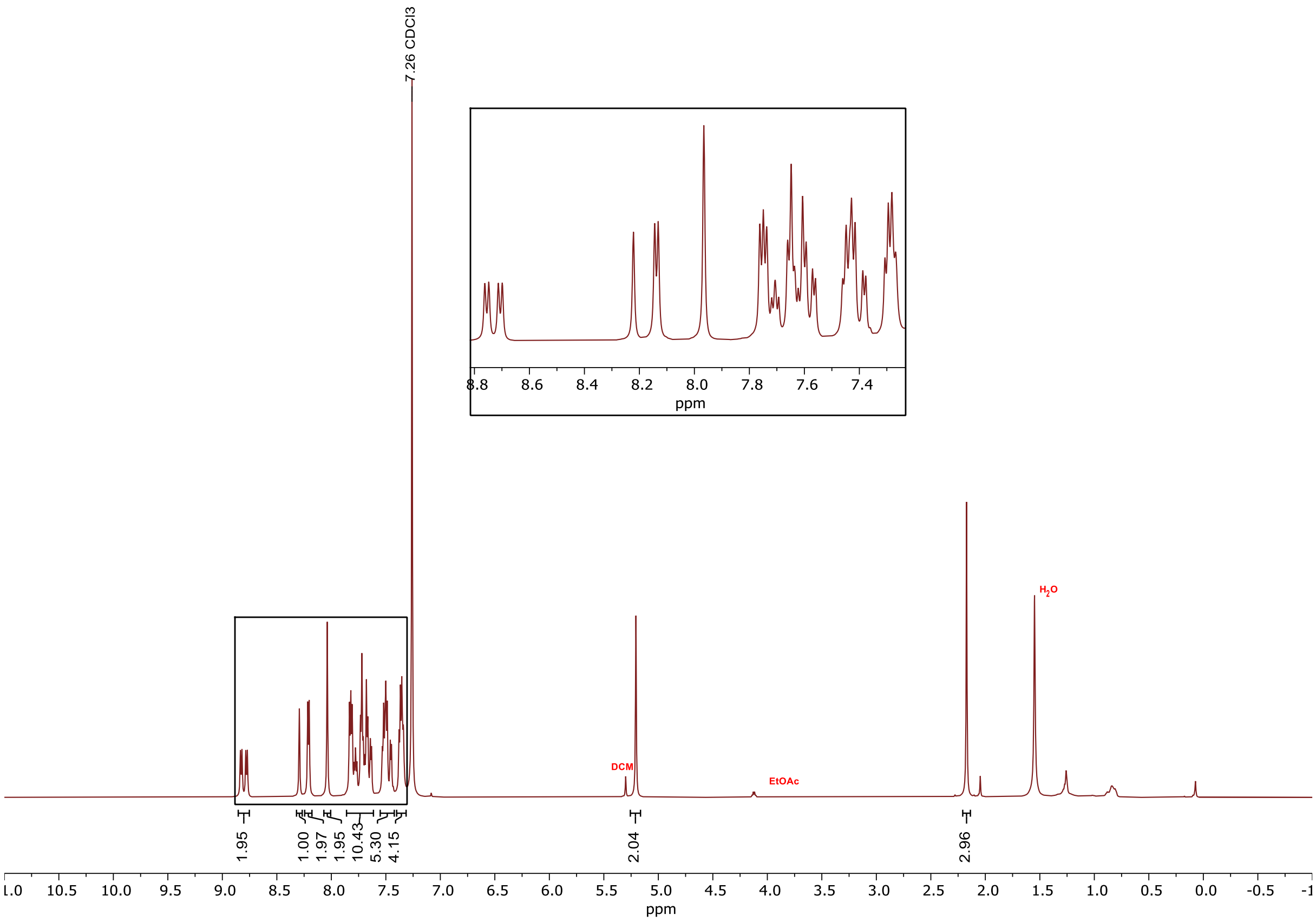

Figure S36. ^{1}H NMR spectrum of **8** in $CDCl_3$.

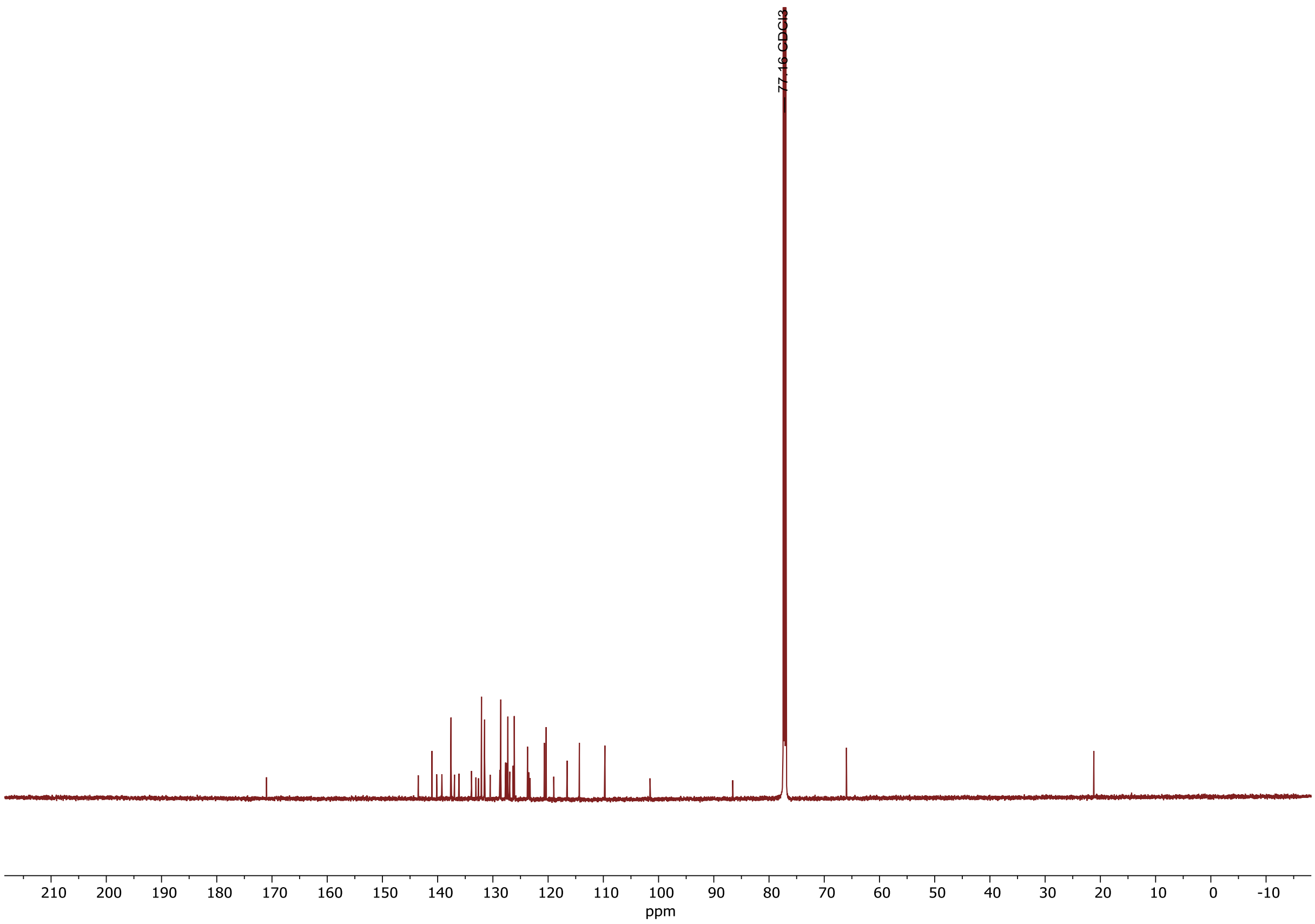

Figure S37. ^{13}C NMR spectrum of **8** in $CDCl_3$.

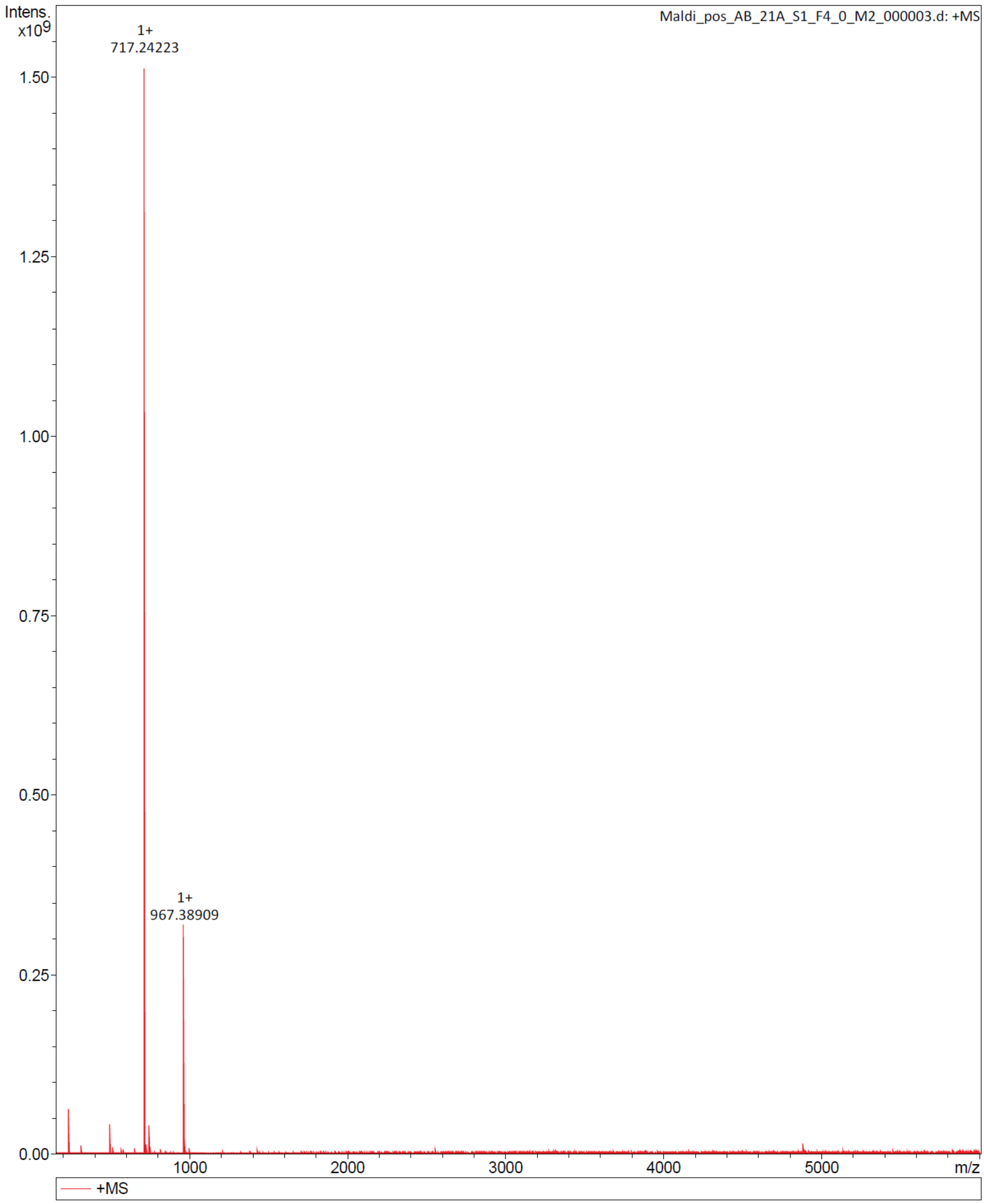

Figure S38. MALDI mass spectrum of substance **8** in DCTB.

6 References

[1] V. Greussing, D. Werner, D. Wielend, C. V. Irimia, E. Leeb, M. Ciganek, J. Krajčovič, M. Irimia-Vladu, E. Portenkirchner, “Evaluation of the Interphase-Related Cycling Stability of Thin-Film Amino- and Hydroxy-Substituted Anthraquinone Electrodes for Sodium-Ion Batteries” *ACS Appl. Energy Mater.* **2026**, *9*, 1638–1646.

[2] X. Wang, H. Sun, Y. Cheng, W. Yang, X. Wang, L. Xiong, Y. Li, J. Wang, C. Gan, C. Lin, H. Xu, “Ethyl acetate as an acetyl source and solvent for acetylation of alcohols by KOH” *RSC Adv.* **2024**, *14*, 12574–12579.

[3] H. L. Lee, K. H. Lee, J. Y. Lee, W. P. Hong, “Management of thermally activated delayed fluorescence using a secondary electron accepting unit in thermally activated delayed fluorescent emitters” *J. Mater. Chem. C* **2019**, *7*, 6465–6474.

[4] M. J. Frisch and G. W. Trucks and H. B. Schlegel and G. E. Scuseria and M. A. Robb and J. R. Cheeseman and G. Scalmani and V. Barone and G. A. Petersson and H. Nakatsuji and X. Li and M. Caricato and A. V. Marenich and J. Bloino and B. G. Janesko and R. Gomperts and B. Mennucci and H. P. Hratchian and J. V. Ortiz and A. F. Izmaylov and J. L. Sonnenberg and D. Williams-Young and F. Ding and F. Lipparini and F. Egidi and J. Goings and B. Peng and A. Petrone and T. Henderson and D. Ranasinghe and V. G. Zakrzewski and J. Gao and N. Rega and G. Zheng and W. Liang and M. Hada and M. Ehara and K. Toyota and R. Fukuda and J. Hasegawa and M. Ishida and T. Nakajima and Y. Honda and O. Kitao and H. Nakai and T. Vreven and K. Throssell and Montgomery, {Jr.}, J. A. and J. E. Peralta and F. Ogliaro and M. J. Bearpark and J. J. Heyd and E. N. Brothers and K. N. Kudin and V. N. Staroverov and T. A. Keith and R. Kobayashi and J. Normand and K. Raghavachari and A. P. Rendell and J. C. Burant and S. S. Iyengar and J. Tomasi and M. Cossi and J. M. Millam and M. Klene and C. Adamo and R. Cammi and J. W. Ochterski and R. L. Martin and K. Morokuma and O. Farkas and J. B. Foresman and D. J. Fox **2016**.

[5] A. L. Schleper, K. Goushi, C. Bannwarth, B. Haehnle, P. J. Welscher, C. Adachi, A. J. C. Kuehne, “Hot exciplexes in U-shaped TADF molecules with emission from locally excited states” *Nat Commun* **2021**, *12*, 6179.

[6] M. Pérez-Trujillo, I. Maestre, C. Jaime, A. Alvarez-Larena, J. F. Piniella, A. Virgili, “Enantioselective preparation and structural and conformational analysis of the chiral solvating agent α,α'-bis(trifluoromethyl)-1,8-anthracenedimethanol” *Tetrahedron: Asymmetry* **2005**, *16*, 3084–3093.
